# Immune dysfunction signatures predict outcomes and define checkpoint blockade–unresponsive microenvironments in acute myeloid leukemia

**DOI:** 10.1172/JCI159579

**Published:** 2022-11-01

**Authors:** Sergio Rutella, Jayakumar Vadakekolathu, Francesco Mazziotta, Stephen Reeder, Tung-On Yau, Rupkatha Mukhopadhyay, Benjamin Dickins, Heidi Altmann, Michael Kramer, Hanna A. Knaus, Bruce R. Blazar, Vedran Radojcic, Joshua F. Zeidner, Andrea Arruda, Bofei Wang, Hussein A. Abbas, Mark D. Minden, Sarah K. Tasian, Martin Bornhäuser, Ivana Gojo, Leo Luznik

**Affiliations:** 1 John van Geest Cancer Research Centre, Nottingham Trent University, Nottingham, United Kingdom.; 2 Department of Oncology and Sidney Kimmel Comprehensive Cancer Center, The Johns Hopkins University School of Medicine, Baltimore, Maryland, USA.; 3 Department of Medicine, Universitätsklinikum Carl Gustav Carus, Technische Universität (TU) Dresden, Dresden, Germany.; 4 Department of Medicine, Medical University of Vienna, Vienna, Austria.; 5 Masonic Cancer Center and Department of Pediatrics, Division of Blood & Marrow Transplant and Cellular Therapy, University of Minnesota, Minneapolis, Minnesota, USA.; 6 Division of Hematology and Hematologic Malignancies, Department of Internal Medicine, University of Utah, Salt Lake City, Utah, USA.; 7 Huntsman Cancer Institute, University of Utah, Salt Lake City, Utah, USA.; 8 Division of Hematology, Department of Medicine, University of North Carolina School of Medicine, Chapel Hill, North Carolina, USA.; 9 Division of Medical Oncology and Hematology, Princess Margaret Cancer Centre, Toronto, Canada.; 10 Department of Leukemia, Division of Cancer Medicine and; 11 Department of Genomic Medicine, The University of Texas MD Anderson Cancer Center, Houston, Texas, USA.; 12 Department of Pediatrics, Division of Oncology and Centre for Childhood Cancer Research, Children’s Hospital of Philadelphia and University of Pennsylvania School of Medicine, Philadelphia, Pennsylvania, USA.; 13 National Center for Tumor Diseases and German Cancer Consortium, Partner Site Dresden, Dresden, Germany.; 14 German Cancer Research Centre, Heidelberg, Germany.

**Keywords:** Hematology, Cancer immunotherapy, Cellular senescence, Leukemias

## Abstract

**Background:**

Immune exhaustion and senescence are dominant dysfunctional states of effector T cells and major hurdles for the success of cancer immunotherapy. In the current study, we characterized how acute myeloid leukemia (AML) promotes the generation of senescent-like CD8^+^ T cells and whether they have prognostic relevance.

**METHODS:**

We analyzed NanoString, bulk RNA-Seq and single-cell RNA-Seq data from independent clinical cohorts comprising 1,896 patients treated with chemotherapy and/or immune checkpoint blockade (ICB).

**Results:**

We show that senescent-like bone marrow CD8^+^ T cells were impaired in killing autologous AML blasts and that their proportion negatively correlated with overall survival (OS). We defined what we believe to be new immune effector dysfunction (IED) signatures using 2 gene expression profiling platforms and reported that IED scores correlated with adverse-risk molecular lesions, stemness, and poor outcomes; these scores were a more powerful predictor of OS than 2017-ELN risk or leukemia stem cell (LSC17) scores. IED expression signatures also identified an ICB-unresponsive tumor microenvironment and predicted significantly shorter OS.

**Conclusion:**

The IED scores provided improved AML-risk stratification and could facilitate the delivery of personalized immunotherapies to patients who are most likely to benefit.

**TRIAL REGISTRATION:**

ClinicalTrials.gov; NCT02845297.

**FUNDING:**

John and Lucille van Geest Foundation, Nottingham Trent University’s Health & Wellbeing Strategic Research Theme, NIH/NCI P01CA225618, Genentech-imCORE ML40354, Qatar National Research Fund (NPRP8-2297-3-494).

## Graphical abstract

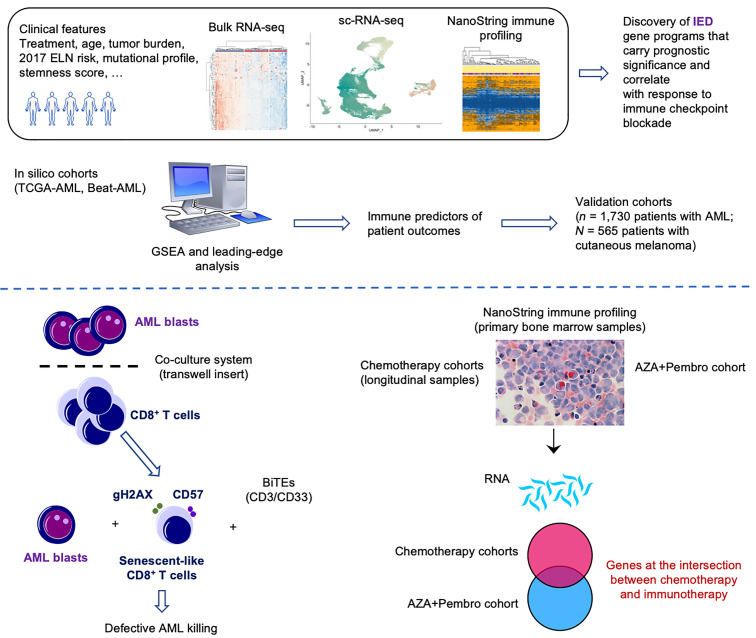

## Introduction

Acute myeloid leukemia (AML) is a molecularly and clinically heterogeneous disease (1). We recently identified BM microenvironmental transcriptomic profiles that stratify patients with newly diagnosed AML into immune-infiltrated and immune-depleted subtypes and that refine the accuracy of overall survival (OS) prediction beyond that afforded by current prognosticators (2). Several aspects of T cell derangement affect AML response to standard-of-care chemotherapy, molecularly targeted therapies, and immunotherapies (2–7). In this respect, IFN-γ–related RNA profiles in baseline BM samples predict response of chemotherapy-refractory AML to CD123×CD3-bispecific molecules (2, 8).

The degree of cytotoxic CD8^+^ T cell infiltration has been shown to correlate inversely with OS in select tumor types, including AML, because of the establishment of highly dysfunctional T cell states (2, 9). Phenotypic and transcriptomic analyses have shown that CD8^+^ T cells from patients with AML exhibit features of exhaustion and senescence. These studies have identified a gene signature that diverges between responders and nonresponders to chemotherapy, with the former exhibiting upregulation of costimulatory pathways and downregulation of apoptotic and coinhibatory T cell signaling pathways (10).

Exhaustion and senescence are dominant dysfunctional states of effector T cells that are increasingly recognized as major hurdles for the success of cancer immunotherapy (11, 12). Senescence and exhaustion share properties, but they may be functionally dissimilar (13). Exhausted T cells express inhibitory receptors, including *PDCD1* (encoding PD-1), *CTLA4*, *HAVCR2* (encoding TIM3), *CD160,* and *2B4* (encoding CD244), and display an impaired ability to secrete effector cytokines and to exert cytotoxic functions. Senescent T cells downregulate costimulatory molecules CD27 and CD28, express senescence-associated surface markers B3GAT1 and KLRG1, as well as MAPK p38 and γ-H2AX intracellular molecules, remain metabolically active and continue to secrete proinflammatory cytokines (14, 15), but their cytotoxic antitumor activity is unclear. While more is known about the role of T cell exhaustion in immunotherapy responses, the contribution of T cell senescence to anticancer immunity is less understood (13).

**Figure 1:**
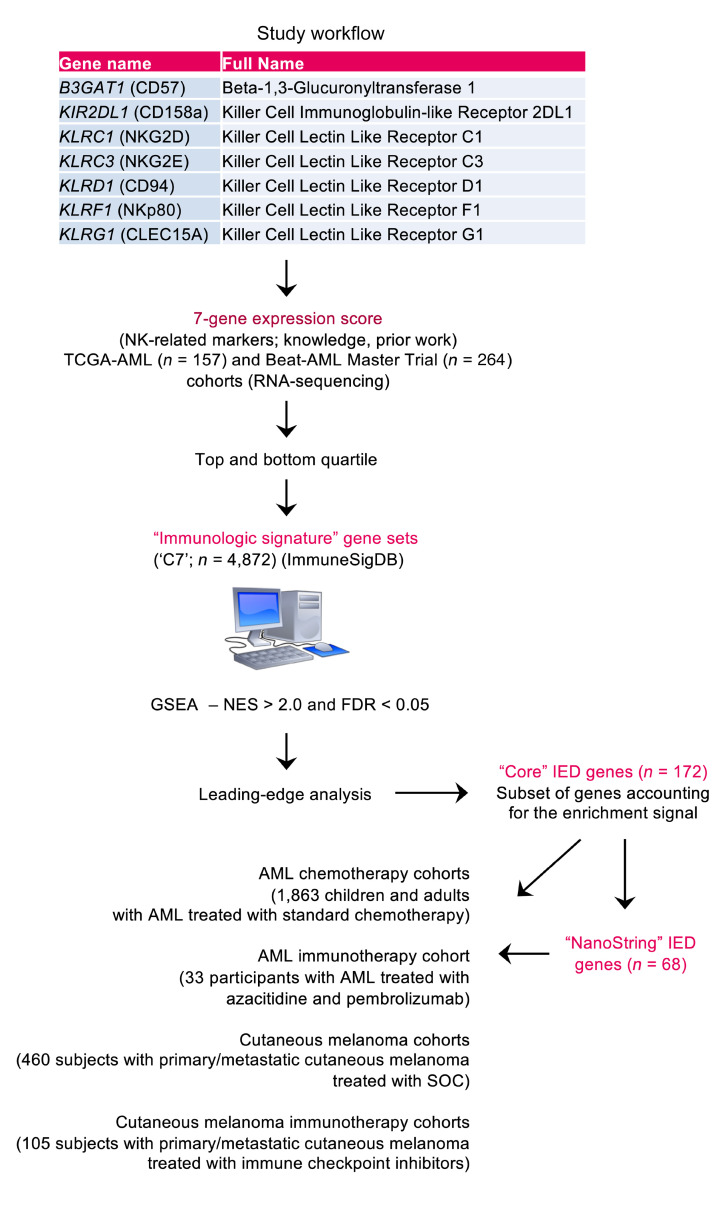
Study workflow. Immune Signature Data Base (100); IED, immune effector dysfunction; NES, normalized enrichment score; FDR, false discovery rate; SOC, standard of care.

In the current study, we characterized how leukemia promotes the generation of senescent-like CD8^+^ T cells and their prognostic relevance in patients with AML. We hypothesized that elucidation of an immune senescence transcriptional signature in the BM of newly diagnosed AML could both identify individuals who are more likely to respond to immunotherapy and predict outcomes. We generated RNA expression data sets from patients treated with conventional cytotoxic chemotherapy or with the hypomethylating agent azacitidine (AZA) in combination with immune checkpoint blockade (ICB) with pembrolizumab (a monoclonal antibody targeting PD-1) (designated as AZA+Pembro). We integrated these with publicly available gene expression data from multiple cohorts of children and adults with AML to validate our RNA metric of immune effector dysfunction (IED), and we analyzed BM samples collected longitudinally at the time of AML onset and response assessment (Figure 1 and Supplemental Figure 1; supplemental material available online with this article; https://doi.org/10.1172/JCI159579DS1). The derived gene signatures of IED correlated with molecular features of leukemia stemness and with distinct clinical characteristics. IED gene sets served as a reliable biomarker to stratify OS after standard-of-care therapy and ICB, both in AML and in melanoma (a paradigm for successful immunotherapy actualization) (16, 17).

## Results

### Functional and transcriptional signature of T cell senescence in AML.

**Figure 2:**
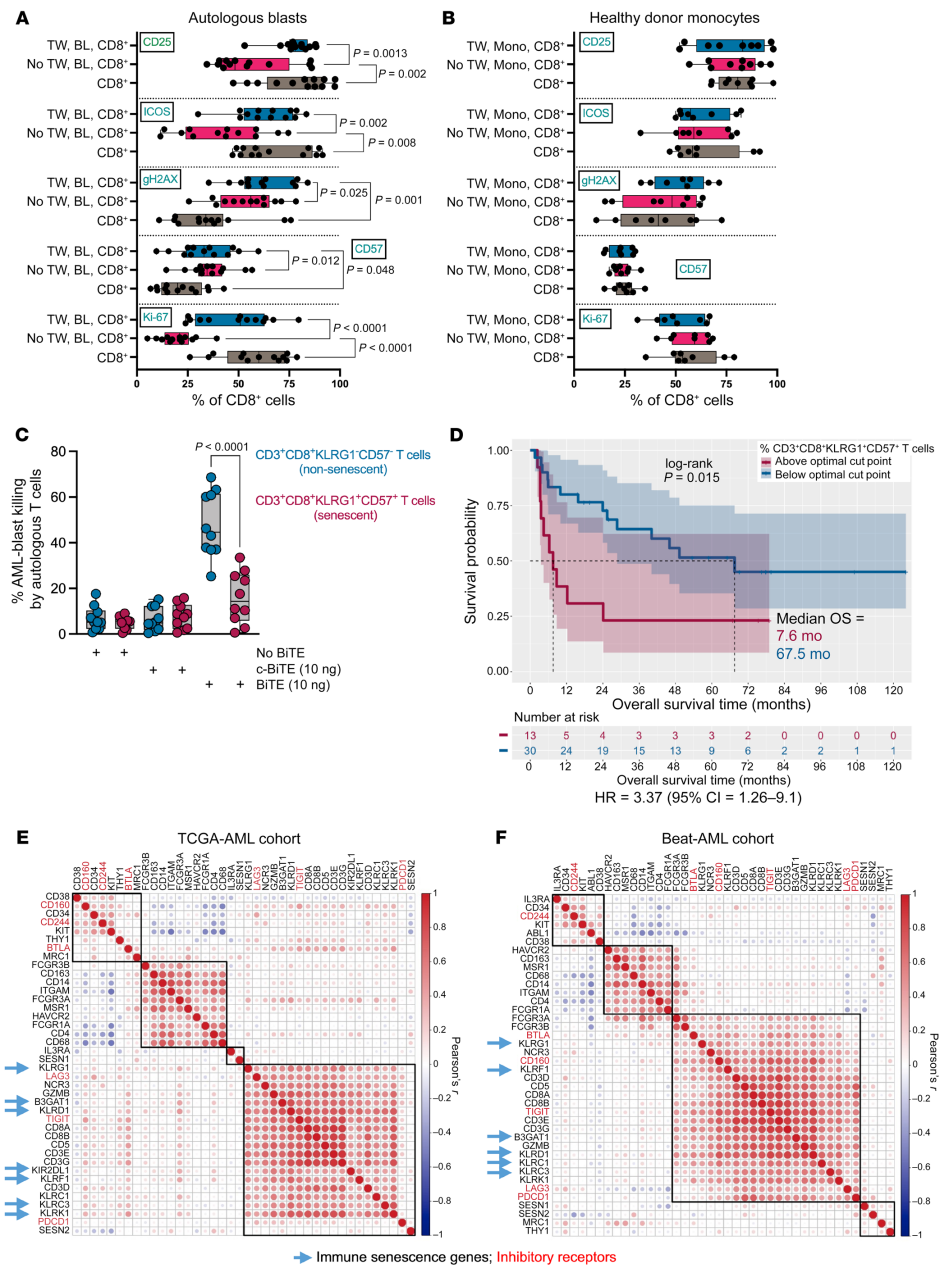
Markers of T cell senescence correlate with impaired T cell killing and poor clinical outcomes. (**A**) Flow-sorted AML blasts were cocultured with autologous, patient-derived CD8^+^ T cells (*n* = 13 patients) for 5 days. Data were compared using the Kruskal-Wallis test. TW = Transwell insert; BL = AML blasts; Mono = monocytes. (**B**) Flow-sorted healthy-donor monocytes were cocultured with patient-derived CD8^+^ T cells (*n* = 9 patients) for 5 days. (**C**) In vitro killing of primary CD33^+^ CD34^+^ AML blasts (*n* = 10 patients) after 48-hour culture with autologous, flow-sorted T cells in the presence of anti-CD33/CD3 and control bispecific T cell engager (BiTE) antibody constructs (effector/target ratio = 1:5). T cell cytotoxicity was determined by flow cytometry, as detailed in the Supplemental Methods. (**D**) Kaplan-Meier estimates of OS in patients (JHU1 cohort, *n* = 43 patients) with senescent T cells above and below the optimal cut point, which was computed using the maxstat package in R. Survival curves were compared using a log-rank test. Median OS is indicated (color-coded by the optimal cut point of the proportion of CD3^+^CD8^+^CD57^+^KLRG1^+^ T cells). (**E** and **F**) Correlograms showing coexpression of NK and T cell markers in (**E**) TCGA-AML and (**F**) Beat-AML cases. The correlation matrix was reordered using the hclust function. Rectangles were drawn based on the results of hierarchical clustering (Euclidean distance, complete linkage). Inhibitory receptors (*CD244*, *BTLA*, *CD160*, *TIGIT*, *LAG3*, and *PDCD1*) are highlighted in red. NK cell, T cell, monocyte-macrophage (*CD14*, *CD68*, and *CD163*), and AML-associated markers (*CD34*, *IL3RA*, *KIT*, and *THY1*) were selected by integrating knowledge from multiple publications (10, 25, 101).

AML blasts are known to be an extrinsic modifier of T cell responses (18–21). Initially, we aimed to experimentally evaluate whether AML blasts affect T cell proliferation, activation, and expression of phenotypic markers of senescence through direct contact or by secreting soluble mediators. Flow cytometry–sorted BM T cells and AML blasts from newly diagnosed patients were cocultured, either in direct contact or separated by Transwell inserts, and stimulated as previously described (10). We found that AML blasts induced expression of 2 well-characterized senescence markers, CD57 and γ-H2AX, on AML CD8^+^ T cells in both experimental conditions. Consistent with previous observations (10), direct contact of AML blasts with T cells resulted in decreased expression of activation/proliferation markers CD25, ICOS, and Ki-67 (Figure 2A). However, when T cells were separated from AML blasts by a Transwell insert, the expression of activation markers (CD25 and ICOS) and Ki-67 equaled that of CD8^+^ T cells stimulated in the absence of AML blasts. These findings suggest that interactions between leukemia blasts and T cells occurring in the local milieu impair T cell activation through direct contact and that induction of senescence markers occurs primarily through bystander modulation. These effects seem to be AML blast–specific, since coculture with monocytes from healthy donors did not affect any of the markers examined (Figure 2B).

Given the high frequency of senescent-like T cells in the BM of patients with AML (10), we next investigated in vitro cytotoxicity of flow-sorted, BM-derived senescent-like (CD3^+^CD8^+^CD57^+^KLRG1^+^) and nonsenescent (CD3^+^CD8^+^CD57^–^KLRG1^–^) T cells against autologous AML blasts using an anti–CD3/CD33 bispecific T cell engaging (BiTE) antibody construct (22, 23). As shown in Figure 2C, senescent-like T cells were significantly impaired in their ability to lyse AML blasts compared with their nonsenescent counterparts. These findings could explain the inferior killing ability of the CD3/CD33 BiTE construct when using patient T cells versus those of healthy controls (24). Analysis of 43 patients with newly diagnosed AML (JHU1 cohort; Supplemental Table 1) also revealed that a higher proportion of senescent-like (CD3^+^CD8^+^CD57^+^KLRG1^+^) T cells in baseline BM samples was associated with significantly worse OS (*P* = 0.004) after treatment with standard chemotherapy (Figure 2D; optimal cut-point of CD3^+^CD8^+^CD57^+^KLRG1^+^ T cells = 31.9%). Senescent-like T cells measured at time of response assessment in 22 patients from the JHU1 cohort who achieved a CR also correlated with shorter OS (Supplemental Figure 2), suggesting that both preexisting senescent-like T cells and those accumulating after chemotherapy might contribute to poor clinical outcomes.

Transcriptional profiling of the tumor microenvironment (TME) has been used to identify immunological signatures, characterize biological processes, and develop predictors of protective immunity (25, 26). We therefore sought to derive gene expression signatures of T cell senescence in the AML BM microenvironment. We compiled a manually curated senescence-related gene set that encompassed *KLRG1*, *CD57* and other senescence markers (*KLRC1*, *KLRC3*, *KLRD1*, *KLRF1*, and *CD158A*) previously shown to be expressed by circulating CD8^+^ T cells from patients with AML (10) and to be upregulated on senescence-like T cells (27) and on dysfunctional chimeric antigen receptor (CAR) T cells (28). We used RNA-Seq data and related clinical information from the TCGA-AML and Beat-AML Master Trial (hereafter Beat-AML) cohorts (*n* = 157 and *n* = 264 unique patients, respectively) and correlated the expression of genes in the immunosenescence signature with markers of immune cells and leukemia blasts.

We found a positive correlation between immunosenescence genes and T cell markers — but not with markers of AML blasts (*CD34*, *CD38*, *IL3RA*, *KIT*), or with markers of accessory cells of the monocyte/macrophage lineage (*CD14*, *CD68*, *CD163*; Figure 2, E and F). The clustering of T cell exhaustion and senescence-associated genes is consistent with our previous flow cytometry studies (10), suggesting that T cells in the AML microenvironment exhibit features of both biological processes (13). Overall, the above findings indicate that cellular and transcriptional signatures of CD8^+^ T cell senescence are present in newly diagnosed AML patients, and that the abundance of senescent-like T cells may correlate with antileukemia responses and OS after induction chemotherapy.

### Identification of a BM IED signature in 2 discovery AML cohorts.

We hypothesized that probing ImmuneSigDB (https://immunespace.org/announcements/home/thread.view?rowId=50) gene sets within the BM microenvironment might reveal core biological processes involved in antitumor immune responses and in therapeutic outcomes. To this end, both TCGA-AML and Beat-AML cases were split into quartiles based on average expression levels of the 7 T cell senescence-associated genes. Gene set enrichment analysis (GSEA) was used to identify core gene sets accounting for the enrichment signal in immunosenescence^hi^ (highest quartile) versus immunosenescence^lo^ cases (lowest quartile). Among the 4,872 curated gene sets from the ImmuneSigDB, only gene sets with a FDR of less than 0.05 and a normalized enrichment score of more than 2.0 (*n* = 123 and *n* = 126 gene sets at the intersection of TCGA-AML and Beat-AML cases, respectively) were carried forward for leading-edge analysis. We reasoned that those genes contained in the leading edge would represent biologically related genes enriched for a phenotype of interest (29). This analysis identified 172 genes that are common to multiple significantly enriched ImmuneSigDB gene sets and that contribute most to the enrichment signal (Supplemental Figure 3A and Supplemental Table 2). The uniform manifold approximation and projection (UMAP) of single-cell RNA-Seq (scRNA-Seq) data from 8 patients in the Institute for Molecular Medicine Finland (FIMM) AML cohort (30) revealed that naive, central memory and effector memory CD4^+^ and CD8^+^ T cells, regulatory T cells, and NK cells were highly enriched in this signature (Supplemental Figure 3, B and C). These findings were further validated in an independent single-cell RNA-Seq cohort from van Galen et al. (31) (Supplemental Figure 4 and Supplemental Figure 5). When mapping gene expression to an integrated scRNA-Seq data set including BM NK cells from healthy controls (32) and from patients with FIMM AML, we found that the 172 genes were predominantly expressed by functionally matured and adaptive NK cells (Supplemental Figures 6 and 7; marker genes from Yang et al., ref. 32 are provided in Supplemental Table 3). However, abnormalities — most often, RNA upregulation and/or gene amplification — in the top 15 genes defining the mature NK cluster, but not the adaptive NK cluster, were associated with worse survival in the TCGA-AML cohort (Supplemental Figure 7).

**Figure 3:**
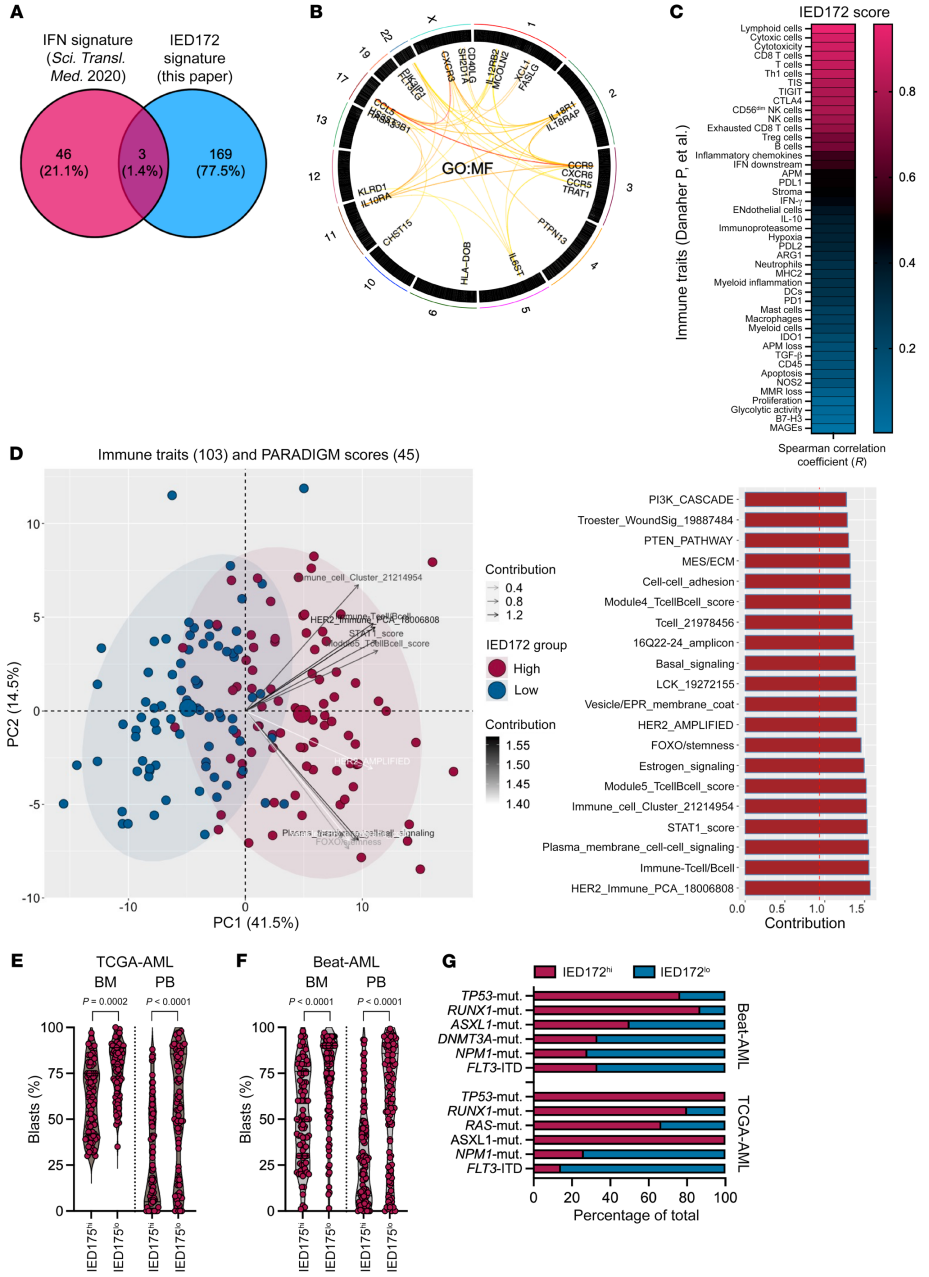
Signatures of immune effector dysfunction correlate with immune infiltration and with adverse-risk molecular features in the TCGA-AML and Beat-AML cohorts. (**A**) Overlap between the IED172 signature genes from this study and published signatures that predict chemotherapy refractoriness as well as response to bispecific T cell engagers (2). IFN, interferon; IED, Immune effector dysfunction. (**B**) Semantic similarity between the IED172 genes in the context of their chromosomal location (*XGR* [eXploring Genomic Relations] web tool [ref. 102]). The degree of similarity between genes is visualized by the color of the links, with light yellow representing a low degree of similarity and red representing more. The chromosomal locus of each gene is indicated by the numbers and colors along the outer rim of the diagram. GO:MF, gene ontology molecular functions. (**C**) Correlation between the IED172 score and previously published immune traits (*n* = 45) (2, 42) in TCGA-AML (*n* = 157 patients). Signature scores are available through the original publications. (**D**) Correlation between the IED172 score and previously published immune traits and PARADIGM scores (*n* = 68; downloaded from the UCSC Xena data portal [https://xenabrowser.net/datapages/]; refs. 40, 44). The principal component analysis (PCA) plot was generated using the ggfortify and ggplot2 R packages. The top contributors to the first and second PC (*n* = 20) are shown as a bar graph. The dotted reference line in the bar graph indicates the expected value if the contribution were uniform. Any feature above the reference line can be considered as important in contributing to the dimension. (**E**) IED172 scores and percentage of blasts at diagnosis in TCGA-AML cases. Data were compared using the Mann-Whitney *U* test for unpaired determinations. PB = peripheral blood. (**F**) IED172 scores and leukemia burden at diagnosis in Beat-AML cases (*n* = 264). Data were compared using the Mann-Whitney *U* test for unpaired determinations. (**G**) Stacked bar graph showing the proportion of IED172^hi^ and IED172^lo^ cases harboring mutations of *TP53*, *RUNX1*, *ASXL1*, *DNMT3A*, *NPM1*, and *FLT3*–internal tandem duplication (ITD). Mut, mutated.

Flow cytometric and bulk RNA-Seq studies have suggested that features of cellular senescence are manifested by T cells in all differentiation states (27, 33). The 172 genes showed broad transcriptional overlap among multiple effector subsets and were enriched in markers associated with T and NK cell recruitment (*CXCR3*, *CCR7*, *CXCR6*), dysfunction and/or exhaustion (*ID3*, *EOMES*, and *SLAMF6*) (28), and senescence (*SESN3*, *IFNG*, and *ETS1*) (27). We hereafter refer to this IED gene set as the IED172 signature. The IED172 genes were nonredundant with knowledge-based transcriptional signatures of T cell exhaustion, CAR T cell dysfunction (28), solid tumor response to ICB (Supplemental Figure 8A and Supplemental Table 4) (34, 35), and IFN-γ–related RNA profiles carrying prognostic significance in AML (Figure 3A) (2). The semantic similarity between IED172 genes in the context of their chromosomal location is shown in Figure 3B. No genes in the IED172 signature were on chromosome 7, the loss of which has been associated with failure to respond to PD-1 blockade (36). Furthermore, IED172 genes were enriched in Kyoto Encyclopedia of Genes and Genomes pathways related to T helper differentiation, T cell receptor (TCR) signaling, and T and NK cell–mediated cytotoxicity (Supplemental Table 5), as well as miRNAs implicated in cancer immune escape and immune metabolism (37–39) (Supplemental Figure 8, B and C). Using a broad collection of immune gene sets (40–43), we found that IED states correlated with lymphoid cells, CD8^+^ T cell and NK cell infiltration, the tumor inflammation signature score, and immune checkpoints *TIGIT*, *CTLA4*, and *PD-L1* (Figure 3C). A principal component analysis with the dependent variables of publically available immune signatures and PARADIGM-integrated pathways further supported the association between IED states and immune infiltration. It also identified T cell and B cell scores, STAT1 signaling, and stemness-related pathways as the top discriminative features (Figure 3D and Supplemental Table 6).

We looked for correlations between the IED172 score and pretreatment variables in diagnostic samples from the TCGA-AML and Beat-AML cohorts (Supplemental Table 7) (46). We found that the IED172 score did not correlate with patient age at diagnosis, 2017 European LeukemiaNet (ELN; https://www.leukemia-net.org/home/) risk category, or mutation count (Supplemental Figure 9, A and B), and that it was higher in AML cases with low leukemia burden (Figure 3, E and F) or in those harboring *TP53*, *RUNX1*, *ASXL1*, and *RAS* mutations (Figure 3G). These findings are consistent with previous reports on the immune landscape of *TP53*- and *RUNX1*-mutated AML (47, 48) and on the inverse correlation between immune infiltration and percentage of blasts, i.e., tumor purity (49). The analysis of Beat-AML cases (*n* = 264, of which 195 have chemotherapy-response data) revealed significantly higher IED172 scores at baseline in patients with primary induction failure (PIF; *n* = 63) compared with those achieving complete remission (CR; *n* = 132; *P* = 0.0044; Supplemental Figure 10, A and B). When analyzing matched samples collected at baseline and after induction chemotherapy (available only for 13 patients in the Beat-AML series), we found that the IED172 score was significantly higher in BM samples obtained at the time of response assessment — CR with measurable residual disease and relapse — compared with the baseline (*P* = 0.0046; Supplemental Figure 10, C and D). Immune cell type deconvolution with quanTIseq, which estimates an absolute score and therefore allows inter-sample comparisons (50), showed lack of statistically significant differences between baseline and post-chemotherapy samples (Supplemental Figure 10, E and F), suggesting that increased IED scores do not merely reflect a larger fraction of T and NK cells after treatment. When analyzing scRNA-Seq profiles of 11 patients with AML from van Galen et al. (31) for whom serial BM samples were available, we observed significantly higher IED172 scores after chemotherapy, both in responders and in nonresponders (Supplemental Figure 11, A–C).

### IED scores correlate with transcriptomic features of AML stemness and stratify survival.

**Figure 4:**
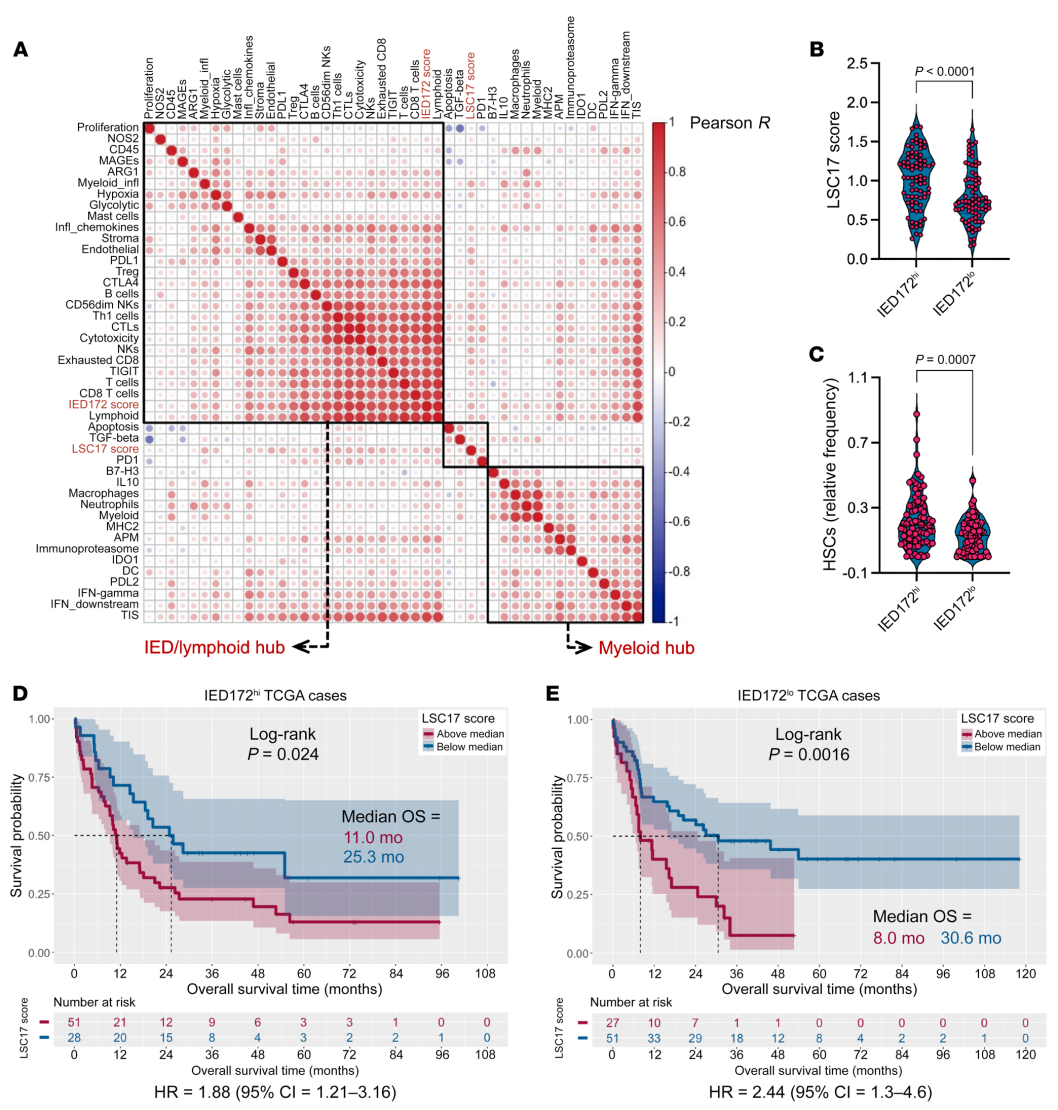
Covarying gene programs of immune effector dysfunction and stemness in the TCGA-AML cohort. (**A**) Pairwise correlation between transcriptomic traits of immune infiltration and LSC17/IED172 scores. Nonsignificant *P* values are shown as blank boxes. Modules, including traits that are densely connected (hubs), are identified based on hierarchical clustering (Euclidean distance, complete linkage) and are shown in black boxes. The IED172 and LSC17 scores are highlighted in red. IED, Immune effector dysfunction. (**B**) LSC17 score in IED172^hi^ and IED172^lo^ TCGA cases (*n* = 157; median split). Data were compared using the Mann-Whitney *U* test for unpaired determinations. (**C**) Inferred relative frequency of hematopoietic stem cells (HSCs) in patients with IED172^hi^ or IED172^lo^, as estimated by xCELL (54). Precalculated TCGA scores were downloaded from https://xcell.ucsf.edu/ (**D**) Kaplan-Meier estimates of overall survival (OS) in patients with IED172^hi^ with above-median and below-median LSC17 scores. Survival curves were compared using a log-rank test. HR, hazard ratio. (**E**) Kaplan-Meier estimates of OS in patients with IED172^lo^ with above-median and below-median LSC17 scores.

The 17-gene leukemia stem cell (LSC17) score has previously been associated with poor clinical outcomes and with *TP53* and *RUNX1* mutational status in de novo AML (51, 52). The LSC17 score discriminated survival outcomes in TCGA-AML and in Beat-AML patient cohorts (Supplemental Figure 12, A and B). The LSC17 score was not colinear with previously published immune cell type–specific gene signatures (42), immune checkpoints, and IFN-γ–related gene programs (Figure 4A) (53), and it was significantly higher in samples with above-median IED172 scores (Figure 4B). This finding was corroborated using xCELL, a single-sample GSEA-based tool that infers cellular content in the TME (Figure 4C) (54). When patients were stratified into IED172^hi^ and IED172^lo^ groups, the LSC17 score continued to predict OS (Figure 4, D and E).

**Figure 5:**
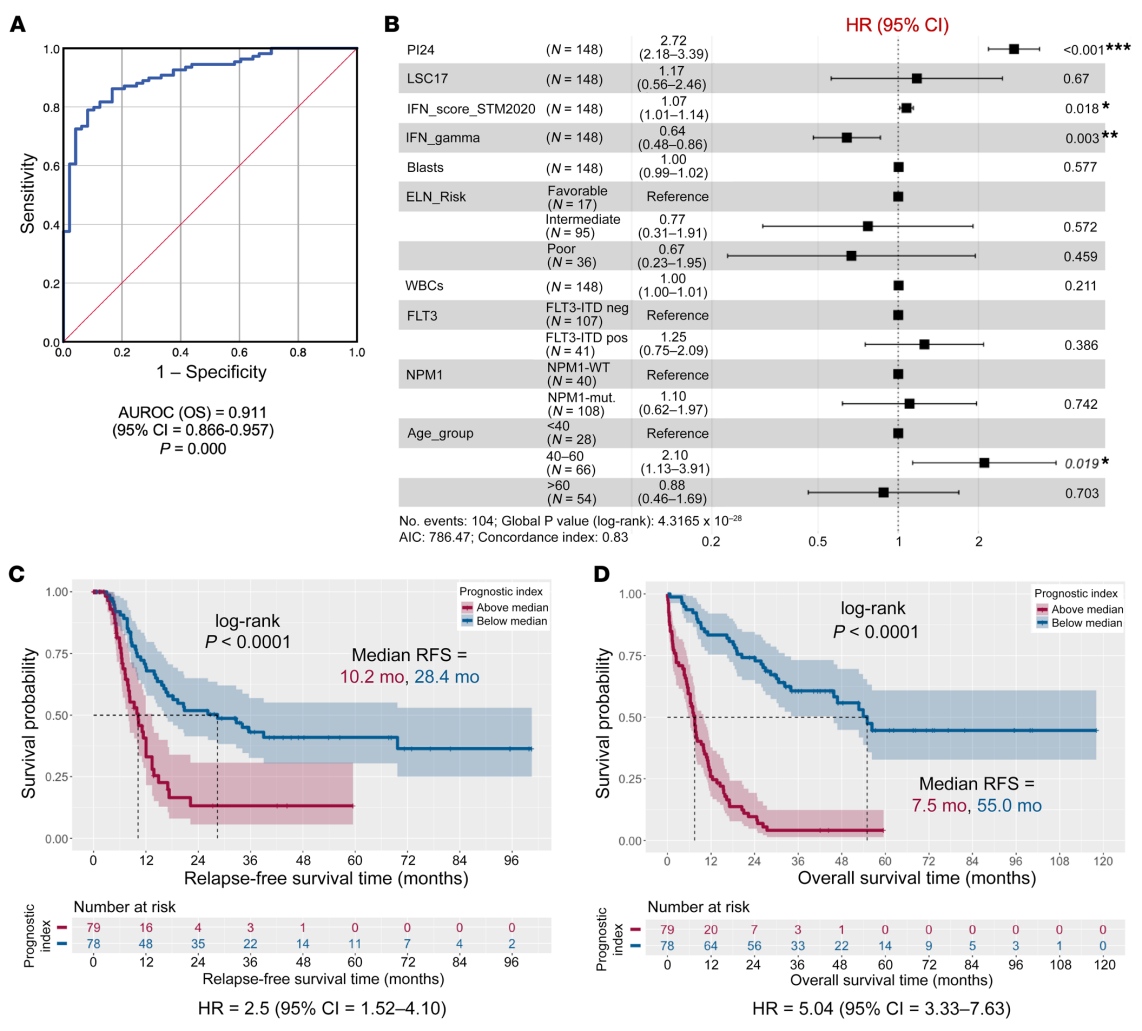
Predictive ability of immune effector dysfunction gene programs in the TCGA-AML cohort. (**A**) Area under the receiver operating characteristic (AUROC) curve measuring the predictive ability of the prognostic index (PI24) genes for overall survival (*n* = 157 TCGA cases). (**B**) Forest plot (ggforest function in survminer package in R) of pretreatment features (WBC count at diagnosis, percentage of bone marrow blasts, *FLT3*-ITD and *NPM1* mutational status, patient age at diagnosis), and RNA-based scores associated with survival in multivariate Cox proportional hazard analyses (PI24, LSC17, and IFN scores; refs. 2, 47, 52). HR = hazard ratio for death. (**C**) Kaplan-Meier estimates of relapse-free survival (RFS) in patients with TCGA-AML with above-median and below-median PI24 scores, which were calculated using β values from Cox regression analyses of gene expression and patient survival (56). Survival curves were compared using the log-rank test. (**D**) Kaplan-Meier estimates of OS in TCGA-AML patients with above-median and below-median PI24 scores. Survival curves were compared using the log-rank test.

To determine the parameters most predictive of OS in the IED172 signature, we used the least absolute shrinkage and selection operator (LASSO) statistical method to fit an L1-regularized linear model (55) that revealed a parsimonious set of 24 genes (Supplemental Table 2). We then generated a prognostic index (PI) using β values from Cox regression analyses of gene expression and OS (Supplemental Figure 13, A and B) (56). The 24-gene PI (PI24) scores inversely correlated with OS time (Supplemental Figure 13C) and was an independent predictor of OS with an area under the receiver operating characteristic (AUROC) value of 0.911 in the TCGA-AML cohort (Figure 5A and Supplemental Figure 13D). In multivariable analyses controlling for tumor purity — based on the percentage of BM blasts — and for patient age, the PI24 score was a more powerful predictor of OS than the LSC17 score (52), the IFN-γ–related score (2), and other established AML prognosticators, including *FLT3*-ITD and *NPM1*-mutational status at diagnosis (Figure 5B). On stratifying patients above or below the median PI24 scores, we found that subjects with an above-median PI24 score experienced significantly shorter relapse-free survival (RFS) and OS (*P* < 0.0001 for both; Figure 5, C and D). Other gene sets related to NK cells and/or capturing cytolytic activity and senescence-associated genes enriched in terminally differentiated CD8^+^ T cells from healthy individuals (27, 30) were unable to stratify survival in TCGA cases (Supplemental Figure 14). High PI24 scores were also associated with significantly inferior OS compared with patients with low PI24 scores in the Beat-AML cohort (*P* = 0.012; Supplemental Figure 15A). In agreement with TCGA data, the PI24 score was a good predictor of OS, with an AUROC value of 0.805 (Supplemental Figure 15B).

As shown in Supplemental Figure 16, A–D, an optimal PI24 cut point of 1.73 parsed the TCGA population into subgroups with maximally different survival probabilities. Furthermore, patients in the highest quartile of PI24 values had poor clinical outcomes (1-year RFS and OS rates of 0% and 3%, respectively) compared with patients in the lowest quartile (1-year RFS and OS rates of 74% and 97%, respectively). These findings were validated in the Beat-AML cohort (Supplemental Figure 16, E and F; optimal cut point = 0.94) and in another large cohort of 562 adult subjects with AML treated on the German AMLCG 1999 trial (GSE37642; Supplemental Figure 17, A and B; optimal cut-points for Affymetrix series GPL570 and GPL96 = 3.84 and 3.67, respectively) (57).

### Validation of IED scores in relation to immune infiltration, stemness, chemotherapy refractoriness, and patient outcome in independent AML cohorts.

**Figure 6:**
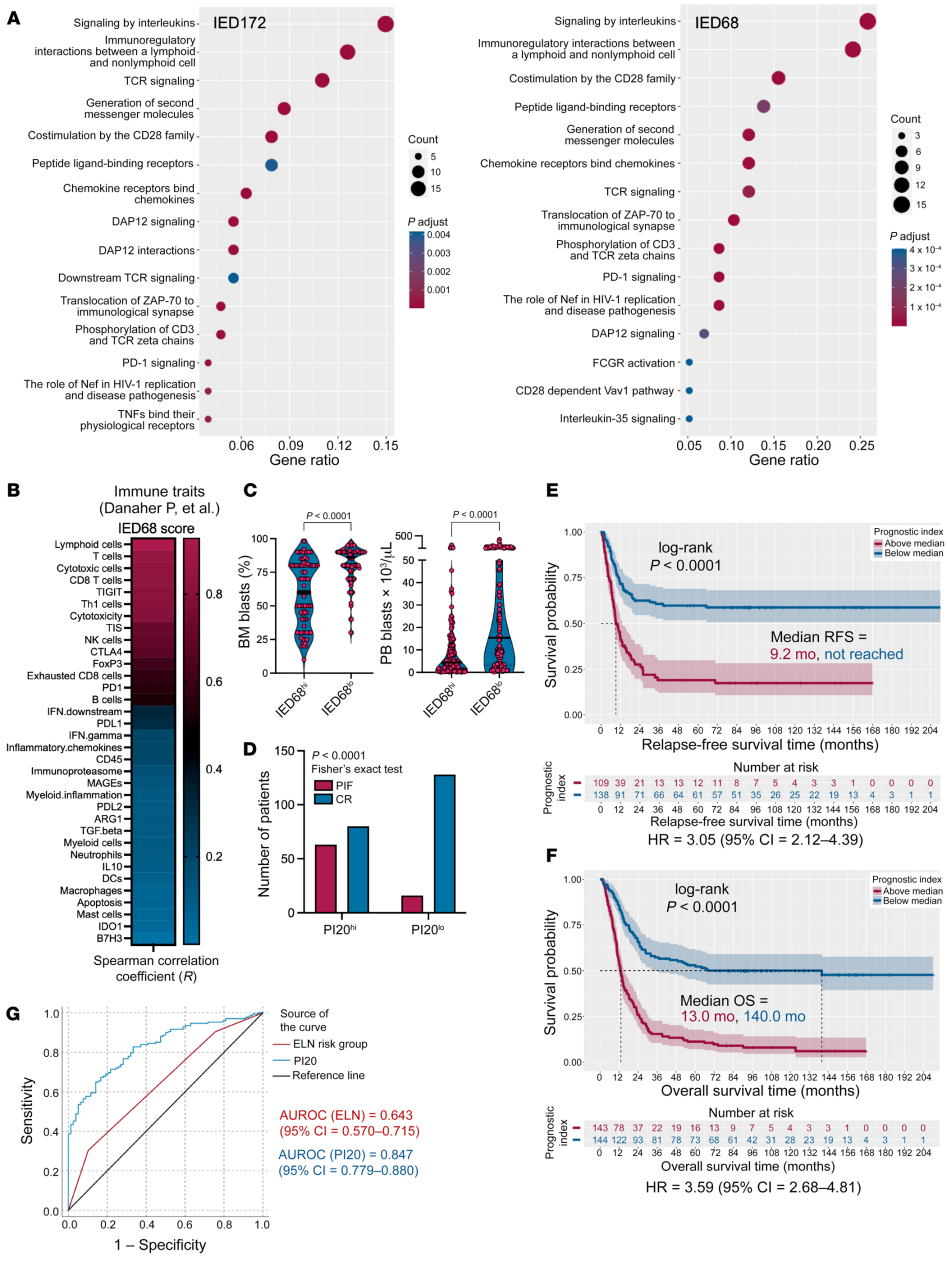
Immune effector dysfunction scores correlate with immune infiltration, stemness, primary induction failure, and patient outcome in an external AML cohort. (**A**) Bubble plot depicting enriched REACTOME pathways (https://reactome.org/) in IED172 and IED68 signature genes (clusterProfiler package in R), which were ranked based on the gene ratio (gene count divided by set size). IED, immune effector dysfunction. (**B**) Correlation between the IED68 score and previously published immune traits (*n* = 45; refs. 2, 42) in the PMCC cohort (*n* = 290 patients). Signature scores are available in the original publications. (**C**) Correlation between IED68 scores and leukemia burden at diagnosis in the PMCC cohort. Data were compared using the Mann-Whitney *U* test for unpaired determinations. BM, bone marrow; PB, peripheral blood. (**D**) Response to induction chemotherapy in patients with above-median and below-median prognostic index (PI20) scores in the PMCC cohort. PIF, primary induction failure following a standard 1 or 2 cycles of induction chemotherapy. CR, complete remission (defined as <5% BM blasts). (**E**) Kaplan-Meier estimates of relapse-free survival (RFS) in PMCC patients with above-median and below-median PI20 scores. Survival curves were compared using a log-rank test. HR, hazard ratio. (**F**) Kaplan-Meier estimates of overall survival (OS) in PMCC patients with higher than median and lower-than-median PI20 scores. Survival curves were compared using a log-rank test. (**G**) Area under the receiver operating characteristic (AUROC) curve measuring the predictive ability of the PI20 and the ELN cytogenetic risk classifier for OS. CI, confidence interval.

Benefiting from our previous work that harnessed large numbers of clinically annotated AML samples (2, 10) and with the aim to develop a gene expression assay that can be rapidly implemented in clinical practice, we turned to the nCounter platform (NanoString Technologies) (52, 58). We initially mined our published AML data set (GSE134589; Princess Margaret Cancer Center [PMCC] cohort encompassing 290 patients with newly diagnosed AML) and identified 68 genes that were shared between the RNA-Seq–based IED172 and NanoString panel (IED68) signatures (Supplemental Table 2). Both the IED172 and the IED68 signatures showed enrichment in genes with annotated functions in cytokine and chemokine signaling, TCR signaling, costimulation by the CD28 family, and PD-1/PD-L1 immune checkpoints in cancer (Figure 6A). As shown in Figure 6B and in agreement with earlier analyses, the IED68 signature was enhanced in tumors that were infiltrated with CD8^+^ and NK cells, characterized by the expression of inhibitory molecules, and inversely correlated with leukemia burden (Figure 6C). Overlaying the IED68 transcriptional signatures onto the UMAP of scRNA-Seq data from Dufva et al. (Supplemental Figure 18; ref. 30) and from van Galen et al. (Supplemental Figure 19, A and B; ref. 31) revealed that, similar to the IED172 score, the IED68 gene set largely mapped to cytotoxic T lymphocyte (CTL) and NK cell clusters, both in AML samples and in BM specimens from healthy controls.

Using LASSO-penalized regression for feature selection and colinearity reduction, we identified 20 genes in the NanoString IED68 signature that were most predictive of OS and that showed minimal overlap with the PI24 genes (Supplemental Figure 13B). We therefore computed a 20-gene PI (PI20) using gene expression values and β coefficients previously derived from Cox proportional hazards (PH) models of the TCGA-AML discovery cohort. The PI20 score was associated with PIF in response to standard chemotherapy (Figure 6D) and with significantly shorter RFS and OS in the PMCC cohort (*P* < 0.001 for both; Figure 6, E and F). Overall, the PI20 score predicted OS with greater accuracy (AUROC value of 0.847) than the 2017-ELN cytogenetic risk classifier (AUROC value of 0.643; Figure 6G and Supplemental Figure 20). These observations were validated in an independent AML series including participants with PIF, enrolled in the AMLCG-2008 study (GSE106291; *n* = 250 patients; Supplemental Figure 21; optimal cut point = 0.1) (57).

Similar to the recently defined IFN-γ gene signature (59), the PI20 score significantly separated survival in patients with intermediate and high ELN risk (Supplemental Figure 22A), as well as after censoring at the time of hematopoietic stem cell transplantation (Supplemental Figure 22B). The latter finding suggests that differences in clinical outcomes between PI20^hi^ and PI20^lo^ cases were not merely attributable to treatment intensity.

We calculated the LSC17 score for the PMCC cohort using publicly available gene expression data (GSE76004) and the same weights as those provided in the original publication (Supplemental Figure 23, A and B) (52). In line with TCGA data, the LSC17 score separated RFS and OS in both PI20^lo^ and PI20^hi^ cases (Supplemental Figure 23, C–F). Specifically, patients with PI20^hi^, a group with a 5-year OS of 11% (Figure 6), were further dichotomized into a subgroup of LSC17^lo^ individuals with an improved 5-year OS probability of 55% (Supplemental Figure 23F). Furthermore, when stratifying patients in the LSC17^hi^ and LSC17^lo^ subgroup by the PI20 scores, we identified a subset of LSC17^hi^ participants with very-poor-prognosis AML, who had 5-year RFS and OS rates of only 10% and 3.5%, respectively (Supplemental Figure 23, G–J).

We formally tested the interaction between senescence- and stemness-related pathways by a multiplication term in the Cox PH model. As shown by the Wald χ^2^ statistics (Supplemental Table 8), the PI20 score was substantially more predictive of OS (*P* < 0.001) in this modeling framework than the LSC17 score (*P* = 0.001). In addition, the interaction between the 2 continuous variables was statistically significant (*P* = 0.013), indicating that a higher PI20 value will increase the association between the LSC17 score and OS. Taken together, these analyses suggest that the PI20 score and its integration with the LSC17 score could provide accurate prognostic risk stratification.

### IED scores predict survival in independent pediatric AML cohorts.

**Figure 7:**
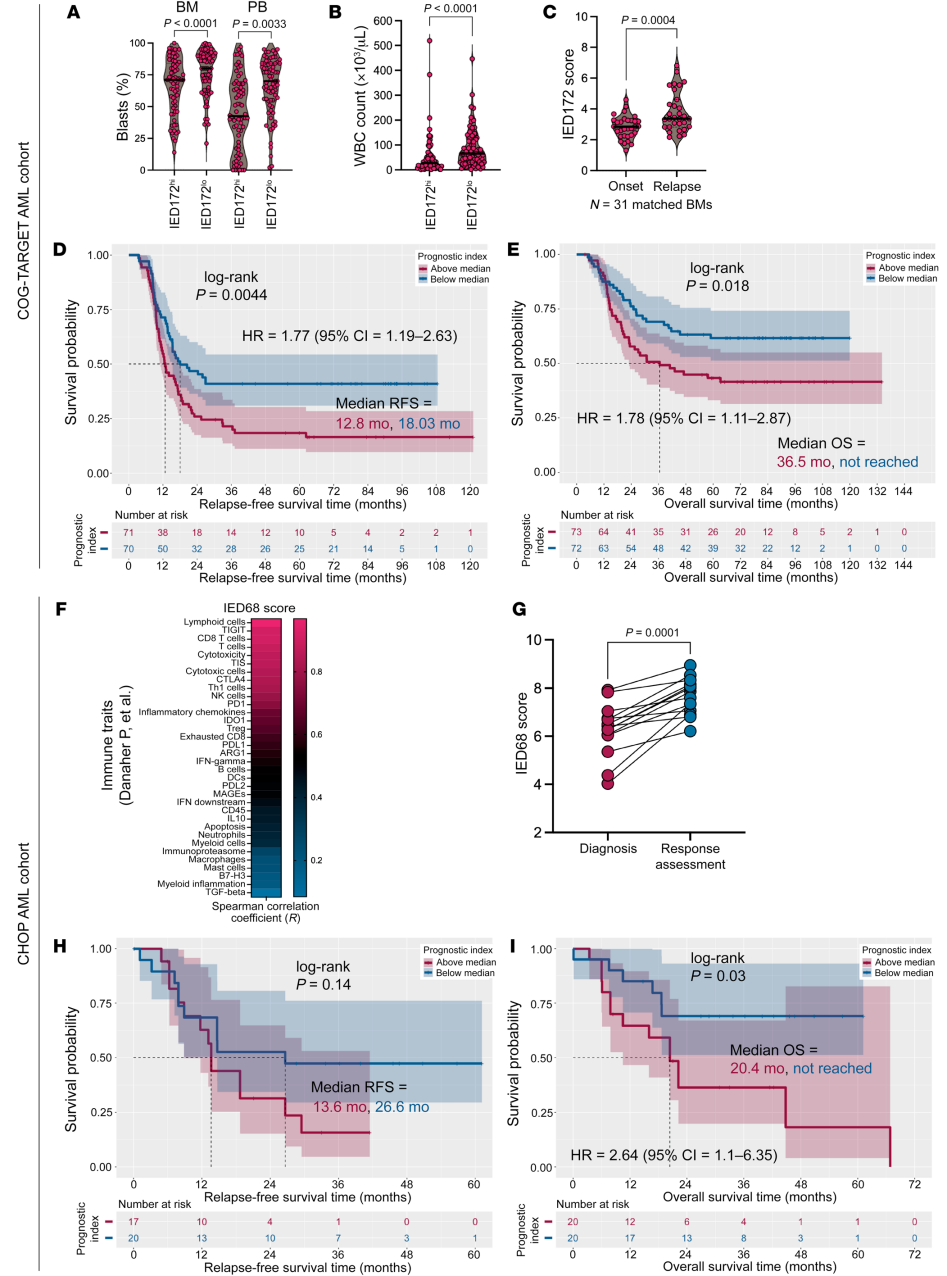
Immune effector dysfunction scores correlate with immune infiltration and separate survival in pediatric AML cohorts. (**A**) Leukemia burden in COG-TARGET AML cases (*n* = 145) with above-median and below-median IED172 scores. Data were compared using the Mann-Whitney *U* test for unpaired determinations. BM, bone marrow; PB, peripheral blood; IED, immune effector dysfunction. (**B**) WBC count at diagnosis in COG-TARGET AML cases with above-median and below-median IED172 scores. Data were compared using the Mann-Whitney *U* test for unpaired determinations. (**C**) IED172 scores at time of AML diagnosis and response assessment (bulk RNA-Seq data from matched BM samples available in 31 COG-TARGET AML cases). (**D**) Kaplan-Meier estimate of relapse-free survival (RFS) in patients from the COG-TARGET AML cohort with above-median and below-median prognostic index (PI24) scores. Survival curves were compared using a log-rank test (survminer package in R). HR, hazard ratio. (**E**) Kaplan-Meier estimate of overall survival (OS) in patients from the COG-TARGET AML cohort with above-median and below-median PI24 scores. (**F**) Correlation between the IED68 score and previously published immune traits (*n* = 45) in the CHOP AML series (*n* = 40). Signature scores are available through the original publications (2, 42). (**G**) IED68 scores in samples from the CHOP AML series collected at time of diagnosis and response assessment (*n* = 14 matched BM samples). Data were compared using the Wilcoxon’s matched-pairs signed-rank test. (**H**) Kaplan-Meier estimates of RFS in patients from the CHOP AML cohort with above-median and below-median PI20 scores. (**I**) Kaplan-Meier estimate of OS in patients from the CHOP cohort with above-median and below-median PI20 scores.

Microenvironmental immune gene sets are known to be differentially expressed between children and adults with AML (2, 60), which may in part be due to differences in pediatric versus adult AML biology (61–64). Furthermore, immunosenescence, a process of remodeling of immune functions upon chronic antigen exposure, is associated with physiologic aging (65, 66). We thus examined the relevance and applicability of the IED score to childhood AML and first analyzed diagnostic BM samples from 145 pediatric patients with de novo AML in the Children’s Oncology Group-TARGET (COG-TARGET) AML cohort for whom RNA-Seq data are publicly available (61). The IED172 score correlated inversely with leukemia burden (Figure 7, A and B) and was significantly higher at time of relapse (*n* = 31 paired BM samples; Figure 7C). Importantly, an above-median PI24 score predicted significantly worse RFS (*P* = 0.0044) and OS (*P* = 0.018; Figure 7, D and E). We then retrieved NanoString transcriptomic data from an additional cohort of pediatric participants with AML (CHOP series, *n* = 40 patients: GSE134589) (2). In line with previous results in adult AML, the IED68 score was higher in children with an immune-infiltrated/activated AML (Figure 7F) and in BM samples obtained at time of response assessment compared with disease onset (Figure 7G). Finally, the PI20 score separated patients into subgroups with different RFS and OS probabilities (Figure 7, H and I). These data support the applicability of IED scores in childhood AML.

### IED scores are increased at time of response assessment.

**Figure 8:**
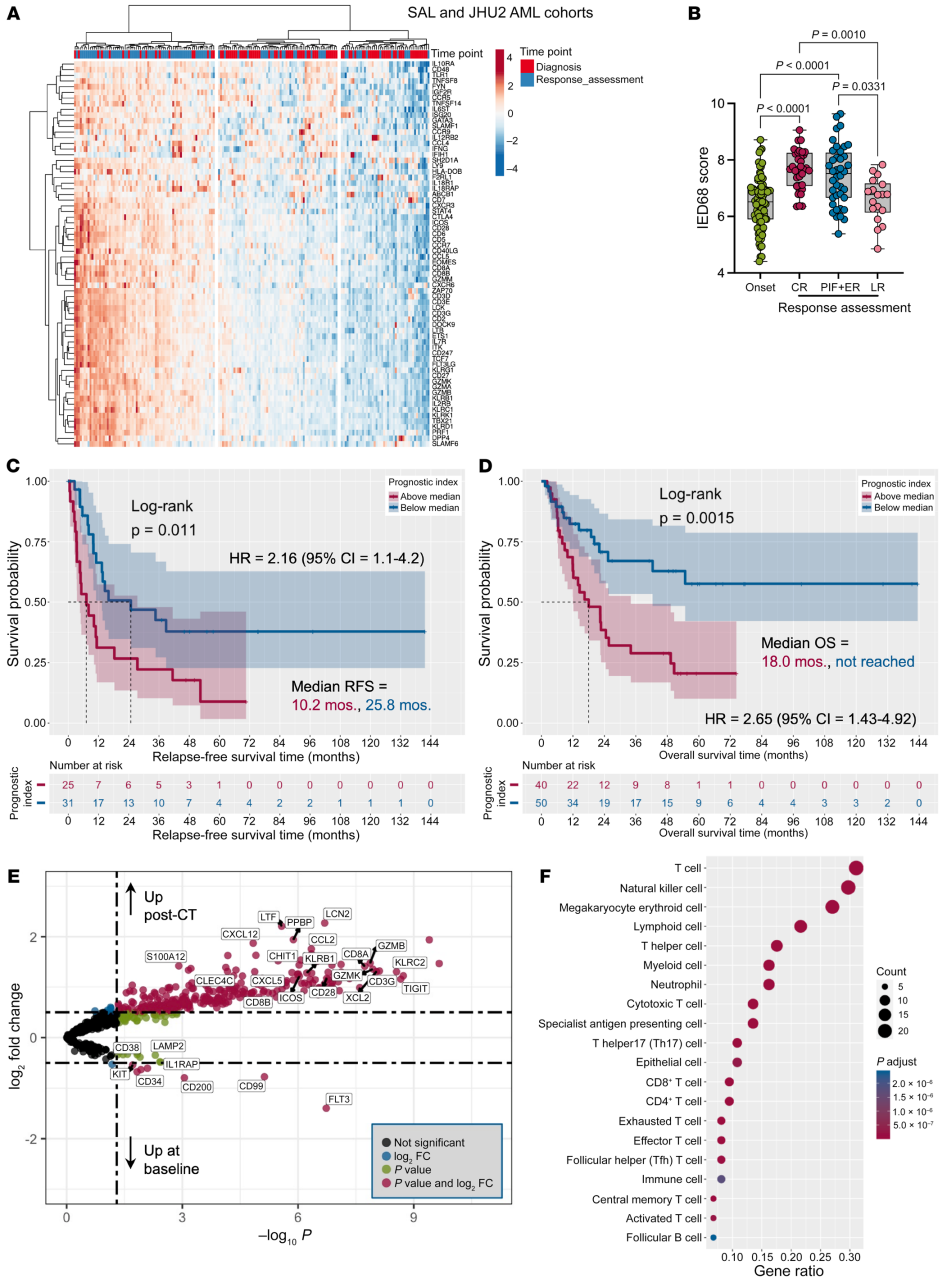
Immune effector dysfunction scores increase at time of response assessment and predict outcomes in additional external AML cohorts. (**A**) Expression of the IED68 genes in patients from the SAL and JHU cohorts (*n* = 183 BM samples from 90 patients). The heatmap annotation track shows sample collection time points (baseline and post-chemotherapy response assessment). (**B**) IED68 score at time of diagnosis and response assessment (Kruskal-Wallis test with correction for multiple comparisons). Nonsignificant *P* values are not shown. CR = complete remission; PIF = primary induction failure; ER = early relapse (<6 months after the achievement of CR); LR = late relapse (>6) months after the achievement of CR); IED = immune effector dysfunction. (**C**) Kaplan-Meier estimate of relapse-free survival (RFS; data available in 56 subjects) in higher-than-median and lower-than-median PI20 groups. HR = hazard ratio. (**D**) Kaplan-Meier estimate of overall survival (OS; data available in 90 subjects) in higher-than-median and lower-than-median PI20 groups. (**E**) Volcano plot showing differentially expressed genes (DEGs) between samples collected at baseline and post-chemotherapy (post-CT) response assessment (EnhancedVolcano package in R). Genes discussed in the paper are named. (**F**) Graphical summary of over-representation analysis (clusterProfiler package in R) showing the overlap between DEGs (post-chemotherapy versus baseline) and curated cell type signature gene sets (C8 collection), which were retrieved from the MSigDB (http://www.gsea-msigdb.org/gsea/index.jsp). Gene ratio = gene count divided by set size.

To further examine the effect of induction chemotherapy on IED scores, we generated nCounter gene expression data using serial BM samples from a large number of donors with newly diagnosed AML (the Studien Allianz Leukämie [SAL] and Johns Hopkins University 2 [JHU2] cohorts), totaling 90 patients and 183 BM specimens that were longitudinally collected at time of diagnosis and response assessment) (Supplemental Table 1). The IED68 scores were significantly higher after chemotherapy, both in patients who achieved CR and in those who experienced PIF or had an early relapse of AML (relapse before 6 months after the achievement of CR; Figure 8, A and B). As expected, the PI20 score separated both RFS (*P* = 0.011; Figure 8C) and OS (*P* = 0.0015; Figure 8D) in this cohort.

Differential expression analysis revealed upregulation of genes involved in T and NK cell biology (*CD3G*, *CD8A*, *CD8B*, *CD28*, *GZMK*, and *GZMB*), cosignaling molecules (*KLRC2*, *KLRB1*, *TIGIT*, *CD40L*, and *ICOS*), myeloid (*LCN2*, *LTF*, and *S100A12*) and dendritic cell differentiation (*CHIT1*, *CLEC4C*), and in chemoattraction (*CXCL5*, *CXCL12*, *CCL2*, *PPBP*, and *XCL2*) after chemotherapy (Figure 8E). As expected, genes associated with AML proliferation (*FLT3*, *KIT*), leukemia stem cells (*CD34*, *CD38*, and *IL1RAP*), and candidate genes overexpressed in AML (*CD99*, *CD200*, and *LAMP2*) were downregulated after chemotherapy, consistent with recent scRNA-Seq and immunohistochemistry data (31). GSEA on the C2 (curated) and C7 (immunologic signature) gene sets from the MSigDB revealed overrepresentation of T cell subsets, NK cells, and antigen-presenting cell signatures (Figure 8F) and clearance of leukemia signatures after chemotherapy (Supplemental Figure 24A). Furthermore, oncogenic pathways were downregulated, while immune signatures were enriched at time of response assessment (Supplemental Figure 24B).

### IED genes define ICB-unresponsive TMEs in AML.

**Figure 9:**
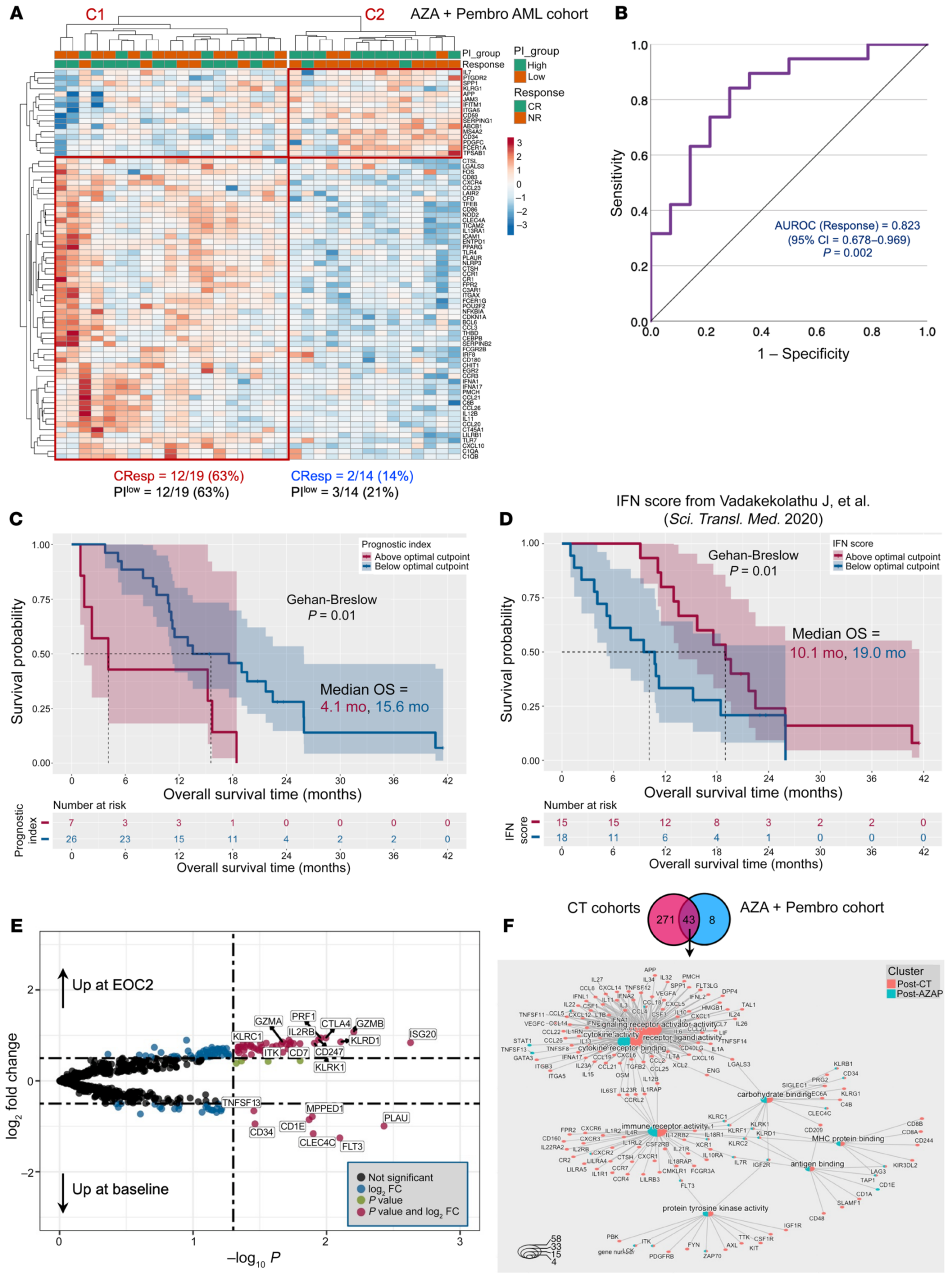
Immune effector dysfunction scores predict response to AZA+Pembro in clinical trial NCT02845297. (**A**) Differentially expressed genes (DEGs) at baseline associated with complete response (CResp) to AZA+Pembro (*n* = 33 patients). The heatmap annotation track shows the prognostic index (PI20) group and response status (complete remission [CR] and nonresponder [NR]) after 2 cycles of azacitidine and pembrolizumab. Complete response was defined as CR, CR with partial hematologic recovery (CRh), CR with incomplete hematologic recovery (CRi), or morphological leukemia-free state (MLFS) at the end of cycle 2. Patients with partial response (PR; >50% decrease in bone marrow blasts from baseline to 5%–25% at the end of cycle 1) were categorized as NRs. C, cluster. (**B**) Area under the receiver operating characteristic (AUROC) curve measuring the predictive ability of IED68 genes for response to AZA+Pembro. CI, confidence interval. (**C**) Kaplan-Meier estimate of overall survival (OS) in patients with above-median and below-median PI20. Survival curves were compared using the Gehan-Breslow-Wilcoxon’s test, a generalization of the Wilcoxon’s rank-sum test that attributes more weight to deaths at early time points. HR, hazard ratio. (**D**) Kaplan-Meier estimate of OS in patients with above-median and below-median IFN scores, which were computed as previously published (2). Survival curves were compared using the Gehan-Breslow-Wilcoxon’s test. (**E**) Volcano plot showing DEGs between baseline and end-of-cycle 2 (EO2) bone marrow samples. The top 20 DEGs are named. (**F**) The overlap between DEGs post-reatment versus baseline in the chemotherapy (CT; SAL and JHU2) and AZA+Pembro patient series is shown as a Venn diagram. Nonredundant, enriched gene ontologies in DEGs between the CT and AZA+Pembro cohorts were visualized as a network diagram (cnetplot) with color nodes using the cnetplot function of the GOSemSim package in R (67).

We assessed the relevance of IED scores in relation to therapeutic response to ICB. We profiled primary BM samples from 33 adult patients with newly diagnosed or relapsed/refractory AML who were treated with AZA+Pembro (ClinicalTrials.gov NCT02845297); Supplemental Table 9; GSE178926). We examined differentially expressed genes (DEGs) at baseline between patients who subsequently achieved CR and those who were nonresponders. Using unsupervised hierarchical clustering of DEGs (Figure 7A), we observed 2 patient clusters. Cluster 1 (C1 in Figure 7A) was enriched for patients who achieved CR (approximately 63%) and for patients with PI20 scores below the median (approximately 63%). In contrast, only approximately 14% of patients in Cluster 2 (C2 in Figure 9A) achieved CR, and approximately 21% of them had below-median PI20 scores (AUROC = 0.823; Figure 9B). Notably, patients with low PI20 scores experienced prolonged OS (median of 15.6 months compared with 4.1 months in patients with high PI20 scores; *P* = 0.01; optimal cut point = 2.44; Figure 9C). We also observed heightened expression of type I and type II IFN signaling genes (*IRF8*, *IFNA1*, *IFNA17*, *CXCL10*, and *CCL20*) in the PI^lo^ group, prompting us to examine the ability of a published IFN signature to predict OS (2). As shown in Figure 9D, high IFN scores were associated with prolonged OS (*P* = 0.01; optimal cut-point = 6.39). The analysis of scRNA-Seq data from 8 patients with relapsed/refractory AML treated with AZA + nivolumab (36) confirmed enrichment of IED68 scores on CD4^+^ and CD8^+^ T cells, NK cells, and NK/T cell precursors (Supplemental Figure 25A). In line with findings in the AZA+Pembro cohort, the IED68 score was significantly lower at baseline in responders to nivolumab-based immunotherapy; Supplemental Figure 25B). Compared with baseline, CD8^+^ T cells, CD4^+^ T cells, and conventional and plasmacytoid DCs from on-treatment BM samples expressed significantly lower IED68 scores (Supplemental Figure 25C). Taken together, these data reveal the unique ability of IED genes to define both chemotherapy- and ICB-unresponsive AML TMEs. By contrast, IFN-γ–related genes have been previously shown to be associated with chemotherapy resistance while also predicting response to T cell engagers (2, 8).

We sought to identify genes at the intersection of responses to chemotherapy and AZA+Pembro. We examined DEGs between matched posttreatment (available in 31 patients after cycle 2) and pretreatment BM samples in the immunotherapy cohort. Treatment with AZA+Pembro resulted in upregulation genes associated with immune effectors (*GZMA*, *GZMB*, *PRF1*, *KLRD1*, and *NCR1*), T cell and NK cell cosignaling molecules (*CTLA4*, *KLRB1*, *KLRC1*, *KLRC2*, and *KLRK1*), cytokine receptors (*IL7R*, *IL2RB*), IFN responsiveness (*ISG20*), and T cell signaling (*CD274*, *ITK*, *CD7*, and *ZAP70*) (Figure 9E). As with the chemotherapy cohort (Figure 8E), AZA+Pembro treatment was associated with downregulation of leukemia-associated genes (*FLT3*, *CD34*). We identified 43 genes that were significantly differentially expressed in both postchemotherapy and post-AZA+Pembro BM samples (Figure 9F), and we then assessed the semantic distance between gene ontologies (GOs) corresponding to these 43 genes using the GOSemSim Bioconductor R package (67). This procedure measures GO and gene similarity, thereby minimizing the redundancy of GO categorization. It identified shared nodes that included GO terms linked to immune functions as well as a prominent “macro-cluster” unique to the chemotherapy setting; and encompassed GO terms and genes related to IFN and cytokine receptor signaling (Figure 9F).

### IED genes define ICB-unresponsive TMEs in melanoma.

**Figure 10:**
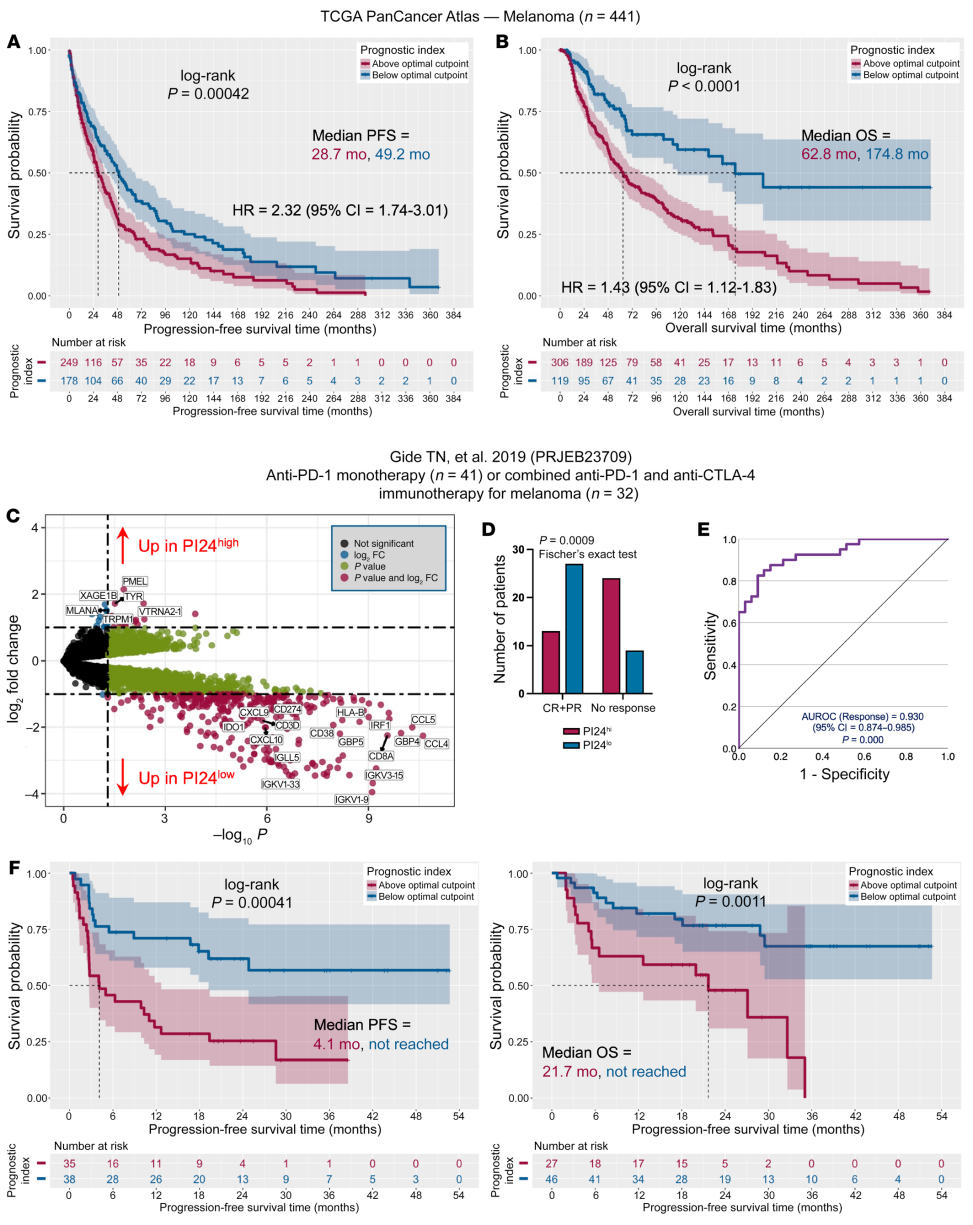
Immune effector dysfunction (IED) scores predict immunotherapy response in melanoma. (**A**) Progression-free survival (PFS) in 427 patients with melanoma from the TCGA Pan-Cancer Atlas profiling project. Participants were stratified based on an optimal cut point of the prognostic index (PI24) (value, 0.862). Survival curves were compared using a log-rank test. RNA-Seq and outcomes data were retrieved through the cBioPortal for Cancer Genomics (https://www.cbioportal.org/). HR, hazard ratio. (**B**) Overall survival (OS) in patients with melanoma from the TCGA Pan-Cancer Atlas cohort. (**C**) Volcano plot showing differentially expressed genes (DEGs) between patients with PI24^hi^ or PI24^lo^ in the PRJEB23709 immunotherapy cohort (73 participants with melanoma treated with standard-of-care single-agent nivolumab or pembrolizumab (*n* = 41) or combination anti–PD-1 + anti–CTLA-4 (*n* = 32; Supplemental Table 10). RNA-Seq and outcome data were retrieved through the original publication (72) and the Tumor Immune Dysfunction and Exclusion (TIDE) portal (http://tide.dfci.harvard.edu/login/) (58). The top 15 DEGs are named. (**D**) Number of responders and nonresponders with above-median and below-median PI24 scores in the PRJEB23709 immunotherapy cohort. Fisher’s exact test. CR = complete response; PR, partial response. In the original publication (72), responders are defined as individuals with complete response, partial response, or stable disease of greater than 6 months with no progression, whereas nonresponders are defined as progressive disease or stable disease for less than or equal to 6 months before disease progression. (**E**) AUROC curve measuring the predictive ability of PI24 genes for response to ICB-based therapies in the PRJEB23709 cohort. CI, confidence interval. (**F**) PFS and OS in patients with melanoma in the PRJEB23709 immunotherapy cohort. Patients were dichotomized based on an optimal cut point of PI24 values (0.12 and 0.344 for PFS and OS, respectively).

To investigate whether these findings can be generalized for ICB-responsive solid tumor types, we conducted an exploratory analysis of IED and its correlation with response to ICB in melanoma. We calculated the PI24 value for patients in the TCGA Pan-Cancer Atlas profiling project (441 subjects with resected primary and/or metastatic melanoma who received no previous systemic therapy) (68). The PI24 score was not correlated with patient age or tumor mutation count (Supplemental Figure 26, A and B) and was lower in patients with an immune-enriched (IE) TME, as defined by Bagaev et al. (25), and with high expression of immune-associated functional gene signatures (Supplemental Figure 26, C and D). As observed above in AML, PFS and OS rates were lower for melanoma cases with high PI24 scores (Figure 10, A and B). Interestingly, the PI24 score refined the ability of the IE, ICB-responsive TME profile — but not the depleted TME subtype (25) — to stratify patient survival (Supplemental Figure 26, E and F; optimal cut points = 1.33 and 0.9, respectively). Compared with PI24^lo^ cases, patients in the PI24^hi^ group had lower numbers of lymphocyte clusters and tumor infiltrating lymphocyte (TIL) patches and higher myeloid/macrophage RNA scores (Supplemental Figure 27, A and B; ref. 69). Furthermore, TIL spatial patterns were significantly different between PI24^hi^ and PI24^lo^ melanoma samples, with the latter showing diffusely infiltrative TILs scattered throughout 30% or more of the tumor area (referred to as a “brisk, diffuse” subtype (69); *P* = 0.0006, Fisher’s exact test; Supplemental Figure 27C). These data are consistent with the established role of TILs in controlling tumor growth in untreated melanoma (70). The analysis of scRNA-Seq profiles from malignant, immune, and stromal cells isolated from 19 melanoma samples (71), indicated that PI24 genes were predominantly expressed by NK and T cells but also by a cluster of “undefined” cells with fibroblast-associated genes (*LGALS1*, *CALD1*, *TIMP1*, *EGR1*, and *SPARC*; Supplemental Figure 28, A–C).

We analyzed publicly available RNA-Seq data from 73 melanoma patients treated with standard-of-care single-agent nivolumab or pembrolizumab (*n* = 41) or combination anti-PD-1 + anti-CTLA-4 (*n* = 32; PRJEB23709; Supplemental Table 10) (72). In this series, patients with above-median PI24 scores showed enrichment in melanocyte-associated markers (*MLANA*, *TYR*, and *PMEL*; Figure 10C) and poor response to ICB based on response evaluation criteria in solid tumors (RECIST) (Figure 10D). The ability of PI24 genes to predict lack of response to ICB (AUROC = 0.93) is shown in Figure 10E. As with TCGA Pan-Cancer Atlas data, patients with below-median PI24 scores expressed high levels of immunoglobulin genes, *CD8A*, and chemokine genes (*CCL4*, *CCL5*, and *CXCL10*), and had significantly higher PFS and OS rates (*P* = 0.00041 and *P* = 0.0011, respectively; Figure 10F).

Finally, an unsupervised analysis of scRNA-Seq profiles of immune cells isolated from 48 tumor biopsies taken either at baseline or during treatment with ICB (73) confirmed enrichment of PI24 scores in immune cells (NK cells, effector memory, and central memory CD4^+^ and CD8^+^ T cells) from pretherapy lesions of nonresponders, i.e., patients with progressive or stable disease, compared with responders (complete or partial response; Supplemental Figure 29, A and B). Overall, these findings suggest that signatures of IED might also be applied as potential biomarkers of response to ICB in melanoma.

## Discussion

An unanswered question in AML is whether deranged T cell functions affect the likelihood of therapeutic response to chemotherapy and/or immunotherapy. Our prior efforts to characterize the AML immune TME using transcriptomic and spatial profiling approaches led to the discovery of an IFN-γ–dominant and inflamed BM milieu (2, 8, 74). In the present study, features of deranged T cell function were identified in multiple independent cohorts of adult and pediatric patients with AML (*n* = 1,896) and were found to be associated with leukemia stemness and with poor response to induction chemotherapy. OS prediction afforded by validated clinical cytogenetic categories and experimental LSC17 signatures (1, 52) was improved by the derived IED gene set, which also defined ICB-unresponsive microenvironments.

Determining how dysfunctional T cell states modulate therapeutic response or resistance in AML remains a challenge, partly due to a lack of selective markers that parse exhaustion from senescence (11, 13). We previously detected increased numbers of circulating senescent-like T cells in AML, which were associated with a low likelihood of response to induction chemotherapy (10). Some reports suggest that tumors induce T cell senescence via cancer cell–derived soluble molecules, while other studies implicate CD4^+^ regulatory T cells in this process (75, 76). Herein, we found that AML blasts influence T cell activation and proliferation through direct contact and bystander effects, whereas induction of CD8^+^ T cell senescence appears primarily dependent on the latter. These mechanisms are particularly relevant for hematologic malignancies such as AML, since leukemia blasts are proximate to circulating T cells and, as such, their potential to promote T cell senescence is expected to be greater than peripherally located solid tumors.

It has also been shown that chemotherapy-induced senescence confers higher tumor-initiating potential to AML and solid tumor cell lines compared with nonsenescent tumor cells (77, 78). While we observed an association between stemness and effector senescence programs, an important question to be addressed is whether crosstalk between senescent-like AML cells and immune effectors could amplify immunosuppressive circuits, leading to failed control of residual disease. Senescent-like cells are known to secrete inflammatory chemokines, cytokines, and growth factors in a paracrine fashion, promoting the reprogramming of neighboring cells (79–81). Furthermore, the humoral communication via senescence-associated secretory phenotype factors might accelerate tumor progression by maintaining chronic inflammation (82). In accord with this model, we show that IED signatures that are shared between central memory and effector memory CD4^+^ and CD8^+^ T cells and functionally matured NK cells are enhanced after chemotherapy, both in bulk and in scRNA-Seq data sets. In contrast to T cell exhaustion, immunosenescence states are maintained by intrinsic signaling induced by DNA damage or other stress responses (75, 83, 84). While a subset of the IED signature comprised exhaustion genes, the overlap between the IED score and published T cell exhaustion gene sets was minimal (34, 85).

Enhancing T cell–mediated clearance of AML is an attractive therapeutic strategy, but some ICB trials and BiTE construct trials have met with only limited success (86–88). Multiple mechanisms have been proposed to explain AML resistance to therapeutic attempts to reverse T cell exhaustion by ICB. These include upregulation of alternative checkpoint receptors or diminished T cell infiltration in patients with advanced disease (3, 89). Our data suggest that senescent-like T cells in pretreatment BM samples are unable to lyse AML blasts when activated with the CD3/CD33 BiTE construct. Consistent with this, a higher proportion of senescent-like CD8^+^ T cells in the BM and blood was associated with lower response rates to pembrolizumab sequenced after high-dose cytarabine in relapsed/refractory AML (7). Therefore, this T cell population may underpin resistance to immunotherapy.

Our study also shows that the initially defined immunosenescence signature in AML also predicts worse outcomes in patients receiving AZA+Pembro or nivolumab immunotherapy, and suggests that senescence reversal could be pursued as a strategy to functionally reinvigorate T cells and to improve response rates to ICB and other T cell–targeting immunotherapies (7, 8). The potential clinical utility of senolytics is currently being tested in animal models (82). By analyzing the immune transcriptome of pretreatment samples from the AZA+Pembro cohort, we identified gene sets and biological functions that were enriched in responders. In contrast to the IED score, the IFN-γ signature score was associated with response to ICB. A plausible explanation for this observation is that stemness states negatively affect type I IFN signaling and anticancer immunity, ultimately leading to poor AML cell killing (49). In melanoma — a tumor type known to derive durable clinical benefit from ICB (72, 90–92) — the IED-related gene set was also expressed by a cluster of cells with fibroblast features, in addition to CD8^+^, CD4^+^, and NK cells. Furthermore, it predicted long-term outcomes and objective responses to single-agent nivolumab or pembrolizumab, or to combination anti–PD-1 + anti–CTLA-4. Prospective immunotherapy clinical trials are warranted to validate the translational relevance of the IED signature in solid tumors other than melanoma.

One limitation of our study is that we focused primarily on gene sets pertaining to immune biology. However, efforts to link immunology with genomic subtypes, therapeutic response, and clinical outcomes in AML are in their infancy (2, 30, 47, 93, 94). In contrast, genome-wide transcriptomic approaches and high-dimensional single-cell analyses have been extensively employed to resolve the molecular heterogeneity and clonal diversity of malignant AML cells (31, 95–98). Both scDNA-Seq and scRNA-Seq studies would be required to explore the relationships among T cell differentiation stages, clonal complexity, and AML hierarchies (31, 98); however, a major challenge is the difficulty of acquiring adequate numbers of T cells from the TMEs in which cells of the myeloid lineage are predominant. Future studies will also have to comprehensively characterize the molecular mechanisms underlying the induction of T cell senescence in the AML TME.

Overall, our findings indicate that IED scores offer advantages over signatures of T cell exhaustion which are solely predictive of response to ICB (58, 73, 99). Our approach elucidates the immune contexture of AML in both chemotherapy and ICB settings, enables refinement of risk stratification, and generates hypotheses for further investigation and clinical exploration of strategies to overcome T cell senescence.

## Methods

Full details are provided in Supplemental Methods.

### Study approval.

Primary specimens (nonpromyelocytic AML) and associated clinical data were obtained with written informed consent from the donors in accordance with the Declaration of Helsinki on research protocols approved by the Ethics Committee of TU Dresden and Studienallianz Leukämie, Germany (EK98032010), and by the Institutional Review Boards of the Children’s Hospital of Philadelphia (no. 10-007767) and Johns Hopkins University.

## Author contributions

S Rutella and LL conceptualized and designed the study. S Rutella, JV, FM, TY, RM, S Reeder, VR, and LL developed the methodology. RM, HA, MK, HAK, JFZ, AA, BW, HAA, MDM, SKT, MB, IG, and LL acquired, obtained consent from, and managed patients, as well as processing patient samples. S Rutella, JV, FM, S Reeder, TY, RM, BD, BRB, VR, BW, HAA, MDM, SKT, MB, IG, and LL analyzed and interpreted data. S Rutella wrote the manuscript. S Reeder, JV, FM, RM, BD, BRB, JFZ, SKT, MB, IG, LL reviewed and/or revised the manuscript. S Rutella and LL supervised the study.

## Supplementary Material

Supplemental data

Supplemental tables 1-10

## Version 1 09/13/2022

In-Press Preview

## Version 2 11/01/2022

Electronic publication
