# Supplementary material for: Immune dysfunction signatures predict outcomes and define checkpoint blockade–unresponsive microenvironments in acute myeloid leukemia
Source: J Clin Invest. 2022 Nov 1;132(21):e159579. doi: 10.1172/JCI159579 (PMC9621145; doi:10.1172/JCI159579)
Supplement: Supplemental data [file jci-132-159579-s173.pdf]

SUPPLEMENTARY INFORMATION

Rutella S, *et al*. Signatures of immune dysfunction predict outcomes and define checkpoint blockade-unresponsive microenvironments in acute myeloid leukemia

TABLE OF CONTENTS

METHODS 3

PATIENT COHORTS 3

Chemotherapy 3

Immunotherapy 3

RNA ISOLATION, NCOUNTER DATA QUALITY CONTROL AND NORMALIZATION 3

IN VITRO CYTOTOXICITY ASSAYS 3

Validation Bulk RNA-Seq Datasets (AML) 4

Validation Bulk RNA-Seq Datasets (Melanoma) 5

Signature Calculation 5

GSEA and Leading-Edge Analysis 6

Immune Deconvolution 6

STATISTICS 7

DATA AND MATERIALS AVAILABILITY 7

Single-cell RNA Sequencing Datasets 8

KEY RESOURCES 11

SUPPLEMENTARY FIGURES 14

Figure S1 | Patient cohorts and gene expression platforms utilized in this study. 15

Figure S2 | Senescent-like T cells at time of response assessment and survival in patients with AML in the JHU1 cohort. 16

Figure S3 | Expression of IED genes in FIMM AML cases (single-cell RNA-sequencing). 17

Figure S4 | Expression of IED genes in AML cases from van Galen et al. (single-cell RNA-sequencing). 19

Figure S5 | Expression of IED genes in healthy donor BM samples from van Galen et al. (single-cell RNA-sequencing). 21

Figure S6 | Expression of IED genes by natural killer (NK) cell functional subtypes from patients with AML (FIMM cohort). 23

Figure S7 | Expression of IED genes by natural killer (NK) cell functional subtypes from patients with AML (FIMM cohort). 25

Figure S8 | Signature overlap and pathway analysis. 27

Figure S9 | IED172 scores in TCGA-AML and Beat-AML cases. 29

Figure S10 | IES172 scores in Beat-AML cases. 31

Figure S11A | IED172 scores in AML cases from van Galen et al. 33

Figure S11B-S11C | IED172 scores in AML cases from van Galen et al. 35

Figure S12 | Leukemia stem cell (LSC17) score and survival in the TCGA-AML and Beat-AML Master Trial cohorts. 36

FIGURE S13 | DERIVATION OF A PARSIMONIOUS IMMUNE EFFECTOR DYSFUNCTION (IED) GENE SET USING LASSO PENALIZED REGRESSION FOR FEATURE SELECTION. ... 38
FIGURE S14 | PUBLISHED GENE SETS CAPTURING NK CELLS, CYTOLYTIC ACTIVITY AND IMMUNE SENESCENCE, AND SURVIVAL IN TCGA-AML CASES. ... 39
FIGURE S15 | AN IMMUNE EFFECTOR DYSFUNCTION (IED)-RELATED PROGNOSTIC INDEX (PI24) SEPARATES SURVIVAL IN A VALIDATION AML COHORT. ... 41
FIGURE S16 | IDENTIFICATION OF AN OPTIMAL PROGNOSTIC INDEX (PI24) CUT-POINT IN TCGA-AML AND BEAT-AML CASES. ... 42
FIGURE S17 | PROGNOSTIC INDEX (PI24) AND SURVIVAL IN THE GSE37642 SERIES (GERMAN AMLCG 1999 TRIAL). ... 44
FIGURE S18 | EXPRESSION OF IED-RELATED GENE SETS IN BONE MARROW SAMPLES FROM PATIENTS WITH AML. ... 45
FIGURE S19 | EXPRESSION OF IED-RELATED GENE SETS IN BONE MARROW SAMPLES FROM PATIENTS WITH AML AND FROM HEALTHY DONORS. ... 46
FIGURE S20 | PREDICTIVE ABILITY OF IED-RELATED GENE SETS IN PATIENTS WITH AML IN THE PMCC COHORT. ... 48
FIGURE S21 | PROGNOSTIC INDEX (PI20) AND PRIMARY INDUCTION FAILURE IN PATIENTS WITH AML (N = 250) TREATED IN THE AMLCG-2008 STUDY (NCT01382147). ... 49
FIGURE S22 | NANOSTRING-BASED PROGNOSTIC INDEX (PI20) IN SUBJECTS OF THE INDICATED CYTOGENETIC GROUPING (PRINCESS MARGARET CANCER CENTRE [PMCC] COHORT). ... 51
FIGURE S23 | NANOSTRING-BASED PROGNOSTIC INDEX (PI20) AND SURVIVAL IN LSC17^{HIGH} AND LSC17^{LOW} PATIENTS (PRINCESS MARGARET CANCER CENTRE [PMCC] COHORT). ... 53
FIGURE S24 | GENE SET ENRICHMENT ANALYSIS (GSEA) OF DIFFERENTIALLY EXPRESSED GENES (DEGS) BETWEEN BASELINE AND POST-CHEMOTHERAPY BONE MARROW SAMPLES FROM PATIENTS IN THE SAL AND JHU2 CHEMOTHERAPY COHORTS. ... 54
FIGURE S25 | EXPRESSION OF IED68 GENES IN A SINGLE-CELL RNA-SEQUENCING COHORT OF 8 PATIENTS WITH CHEMOTHERAPY-REFRACTORY AND/OR RELAPSED AML TREATED WITH AZACITIDINE AND NIVOLUMAB IMMUNOTHERAPY. ... 56
FIGURE S26 | IED SCORES IN PATIENTS WITH CUTANEOUS MELANOMA (TCGA SERIES). ... 58
FIGURE S27 | PI24 SCORES IN PATIENTS WITH CUTANEOUS MELANOMA (TCGA SERIES). ... 60
FIGURE S28 | EXPRESSION OF PI24 GENES IN A SINGLE-CELL RNA-SEQUENCING COHORT ENCOMPASSING 19 HUMAN MELANOMA TUMORS (TIROSH ET AL.). ... 62
FIGURE S29 | EXPRESSION OF PI24 GENES IN A SINGLE-CELL RNA-SEQ COHORT ENCOMPASSING 48 TUMOR SAMPLES OF PATIENTS WITH MELANOMA TREATED WITH IMMUNE CHECKPOINT BLOCKADE (SADE-FELDMAN ET AL.). ... 64
REFERENCES ... **66**

METHODS

Patient Cohorts

Chemotherapy

The study workflow is illustrated in Figure 1. Patient and disease characteristics are detailed in Supplementary Table 1.

Immunotherapy

BM samples were obtained from 33 elderly patients with chemotherapy-refractory/early relapsed AML on a phase 2 study of AZA+Pembro (clinicaltrials.gov identifier: NCT02845297). Azacitidine was given intravenously at 75 mg/m^2 daily on days 1 to 7 every 4 weeks, and pembrolizumab was given intravenously at 200 mg on day 8 and every 3 weeks thereafter. Patient and disease characteristics are detailed in Supplementary Table 9.

RNA Isolation, nCounter Data Quality Control and Normalization

RNA was isolated and processed as previously described (1). Briefly, 100-150 ng per sample of RNA extracted from BM aspirates were processed on the nCounter FLEX analysis system (NanoString Technologies) using the PanCancer Immune Profiling (PCI) panel.

The reporter probe counts, i.e., the number of times the color-coded barcode for that gene is detected, were tabulated in a comma separated value (CSV) format for data analysis with the nSolver software package (version 4.0.62) and nSolver Advanced Analysis module (version 2.0.115; NanoString Technologies). The captured transcript counts were normalized to the geometric mean of the housekeeping reference genes included in the assay (n = 40) and the code set's internal positive controls. Batch effects and other unwanted sources of variation were removed using the Surrogate Variable Analysis (SVA) package in Bioconductor.

In vitro Cytotoxicity Assays

Cytotoxicity of senescent ($CD8^+CD57^+KLRG1^+$) and non-senescent ($CD8^+CD57^-KLRG1^-$) T cells against primary AML cells ($CD45^{low}SSC^{int}$) was tested in vitro using anti–CD33/CD3 and

control bi-specific T-cell engager (BiTE) antibody constructs (both provided by Amgen), as previously described (2). Briefly, primary AML samples were sorted into $CD8^{+}CD57^{+}KLRG1^{+}$ T cells, $CD8^{+}CD57^{-}KLRG1^{-}$ T cells and AML blasts. T cells were then co-cultured with primary AML blasts (effector/target [E/T] ratio = 1:5) in Iscove's Modified Dulbecco's Medium (Life Technologies) supplemented with 15% fetal bovine serum, and 10 ng/ml each of IL-3, SCF, G-CSF, and GM-CSF (all from Life Technologies), for 48 hours. Cells were exposed to either BiTE (10 ng/ml) or cBiTE (10 ng/ml). After 48 hours, T-cell cytotoxicity against $CD33^{+}CD34^{+}$ primary AML cells was determined by flow cytometry using the Live/Dead Fixable Yellow Dead Cell Stain Kit (ThermoFisher Scientific).

Validation Bulk RNA-Seq Datasets (AML)

The Cancer Genome Atlas (TCGA-AML) series consisted of RNA-sequencing data (Illumina HiSeq2000) from 147 adult patients with nonpromyelocytic AML who were enrolled on Cancer and Leukemia Group B treatment protocols 8525, 8923, 9621, 9720, 10201 and 19808. RNA and clinical data were retrieved from cBioPortal for Cancer Genomics (https://www.cbioportal.org/) (3). Level 3 RSEM-normalized RNASeqV2 data was downloaded and $\log_2$-transformed prior to analysis. No further pre-processing was applied. For mRNA expression data, cBioPortal for Cancer Genomics computes the relative expression of an individual gene and tumor specimen to the gene's distribution in all samples that are diploid for the gene in question. The returned value (*z*-score) indicates the number of standard deviations away from the mean of expression in all other tumor samples. To ensure high stringency, a *z*-score threshold of ±2.0 was used in all analyses. Patients had a median age of 60 years, 54% were male, with 12%, 65% and 22% classified as favorable, intermediate, and adverse risk, respectively, based on 2017 European Leukemia Net (ELN) risk stratification by genetics. One hundred thirteen patients (77%) were reported as having received "7+3" cytotoxic induction chemotherapy. The remaining patients were treated with adjunctive therapy in addition to "7+3" or with hypomethylating agents.

The second data series (Beat-AML) was retrieved using the VIZOME interface (http://www.vizome.org/aml/) and consisted of RNA-sequencing data (Agilent platform) from primary specimens from 281 patients with nonpromyelocytic AML and detailed clinical annotation, including diagnostic information, responses and outcomes, treated on the Beat AML Master Trial (4, 5).

The third data series, hereafter referred to as the Children's Oncology Group Therapeutically Applicable Research to Generate Effective Treatments (COG-TARGET) AML series, consisted of RNA-sequencing data (Illumina HiSeq2000) from 145 children, adolescents, and young adults with *de novo* AML enrolled onto biology studies and clinical trials managed through the COG on studies CCG-2961, AAML03P1, or AAML0531 (6, 7).

NanoString immune transcriptomic datasets are available through GEO accession number GSE134589 (n = 432 children and adults with newly diagnosed AML) and our previous publication (1).

Validation Bulk RNA-Seq Datasets (Melanoma)

The TCGA Pan-Cancer Atlas series consisted of RNA-sequencing data (Illumina HiSeq2000) from 441 adult patients with untreated primary and/or metastatic melanoma. The PRJEB23709 series encompassed 73 patients with melanoma treated with standard-of-care single-agent nivolumab or pembrolizumab (n = 41) or combination anti-PD-1 + anti-CTLA-4 (n = 32). RNA-sequencing data were retrieved through the original publication (8) and the Tumor Immune Dysfunction and Exclusion (TIDE) portal (http://tide.dfci.harvard.edu/login/) (9). In the original study, responders were defined as individuals with complete response, partial response, or stable disease of greater than 6 months with no progression, and non-responders as progressive disease or stable disease for less than or equal to 6 months before disease progression.

Signature Calculation

The relative abundance of immune cell types was computed as previously published (10, 11).

For each sample, immune gene expression scores were calculated as an average (arithmetic mean) of gene expression values for all genes in the signature.

The LSC17 score was computed as the weighted sum of the normalized expression values of the 17 genes included in the signature using the same weights as those provided in the original publication (12):

LSC17 score =

(*DNMT3B* × 0.0874) + (*ZBTB46* × −0.0347) + (*NYNRIN* × 0.00865) + (*ARHGAP22* × −0.0138) + (*LAPTM4B* × 0.00582) + (*MMRN1* × 0.0258) + (*DPYSL3* × 0.0284) + (*KIAA0125* × 0.0196) + (*CDK6* × −0.0704) + (*CPXM1* × −0.0258) + (*SOCS2* × 0.0271) + (*SMIM24* × −0.0226) + (*EMP1* × 0.0146) + (*NGFRAP1* × 0.0465) + (*CD34* × 0.0338) + (*AKR1C3* × −0.0402) + (*GPR56* × 0.0501).

GSEA and Leading-Edge Analysis

GSEA was performed using the GSEA software v4.1.0 (Broad Institute). A collection of 4,872 gene sets (ImmuneSigDB) derived from 389 published studies of immune cell states and experimental perturbations, both genetic and chemical, was downloaded from https://www.gsea-msigdb.org/gsea/msigdb/index.jsp (13). Each gene set in the ImmuneSigDB contains either up- or downregulated genes only. The GSEA-p software package was used to extract leading-edge genes that contribute most to the enrichment signal and are shared across the top-ranking ImmuneSigDB gene sets (14).

Immune Deconvolution

Immune cell fractions relative to all cells were inferred using *immunedeconv*, an R package for quantifying cell types from bulk RNA-sequencing data. We selected quanTIseq since this method provides an absolute score representing immune cell fractions and therefore allows both intra- and inter-sample comparisons (15, 16).

Statistics

Descriptive statistics included calculation of median, inter-quartile ranges and proportions to summarize study outcomes. Comparisons were performed with the Mann-Whitney U test for paired or unpaired data (two-sided), as appropriate, or with the ANOVA with correction for multiple hypothesis testing. Given the potentially large number of parameters with high correlation and in order to prevent overfitting, we used the Least Absolute Shrinkage and Selection Operator (LASSO) regularization technique for variable reduction (*glmnet* and *penalized* packages in R) (17). Ten-fold internal cross-validation was used to select the optimal λ value (optL1 function). Model performance was estimated through the mean likelihood ratio test statistic across the 10 outer cross-validation splits. This was repeated for 1,000 iterations. Genes with nonzero coefficients were selected as predictive of the outcome variable (patient survival).

Overall survival was computed from the date of diagnosis to the date of death. Relapse-free survival was measured from the date of first complete remission to the date of relapse or death. Subjects lost to follow-up were censored at their date of last known contact. Kaplan-Meier survival plots were generated using the *survminer* package in R and the log-rank (Mantel-Cox) test was used to compare survival distributions. The *P* values were adjusted for multiple hypothesis testing using the Benjamini-Hochberg procedure. A *P* value less than 0.05 was considered significant. IBM SPSS Statistics (version 27), R (version 4.2.0) and GraphPad Prism (version 9.3.1) were used for statistical analyses.

Data and Materials Availability

The transcriptomic datasets generated in this study have been deposited on to the GEO repository under accession numbers GSE176100 and GSE178926 and will be publicly available as of the date of publication. The results published here are in part based upon data generated by the TCGA Research Network and by the TARGET initiative, which can be accessed, queried, and visualized through the cBioPortal for Cancer Genomics (https://www.cbioportal.org/).

Accessions for gene expression and RNA-sequencing data sets used in this study: newly diagnosed AML GEO: GSE134589 (1), newly diagnosed AML TCGA https://www.cbioportal.org/ (3), newly diagnosed AML COG-TARGET https://www.cbioportal.org/ (6), newly diagnosed Beat-AML Master Trial https://www.cbioportal.org/ (4), newly diagnosed AML syn21991338 (18), newly diagnosed AML GEO: GSE76004 (12), newly diagnosed AML (German AMLCG 1999 trial) GEO: GSE37642 (19, 20), newly diagnosed, chemotherapy-resistant AML GEO: GSE106291 (19), untreated cutaneous melanoma TCGA https://www.cbioportal.org/ (21), cutaneous melanoma immunotherapy ENA: PRJEB23709 (8), tumor microenvironment (TME) classification and functional TME gene signatures https://science.bostongene.com/tumor-portrait/ (22).

Codes for reproducibility of data are publicly available.

Gene lists generated in this study are provided in Supplementary Table 2.

Single-cell RNA Sequencing Datasets

Dufva *et al*. (18)

Single-cell RNA sequencing (scRNA) data from eight diagnostic AML samples were retrieved through the Synapse data repository (https://www.synapse.org/#!Synapse:syn21991338). The following objects were analyzed using the *Seurat* package in R (v.4.2.0) without any further processing (23): FIMM_AML_scRNA.rdata, FIMM_AML_HCA_T_scRNA.rdata (integrated reference dataset of BM T cells from the FIMM AML cohort and the Human Cell Atlas [HCA; n = 8 healthy donors], totaling 52,909 profiled cells) and FIMM_AML_HCA_Yang_NK_scRNA.rdata (integrated reference dataset of NK cells from the FIMM AML cohort, the HCA and Yang *et al*. (n = 6 healthy donors) (24), resulting in 26,601 profiled cells). SingleR cell type annotations (ENCODE/Blueprint) were provided in the original publication (18). Single-cell signature scores were estimated with the AddModuleScore_UCell function (UCell v1.0.0 package, available on GitHub at https://github.com/carmonalab/UCell), which is based on the Mann-Whitney *U* statistic and calculates scores based on the relative ranking of genes for individual cells.

van Galen *et al.* (25)

scRNA-seq profiles for 16 AML samples at diagnosis and during treatment (totaling 30,712 transcriptomes) and for 5 healthy BM donors (7,698 cells) were downloaded from GEO: GSE116256. After filtering out cells with >5% mitochondrial gene counts, or <200 or >2,500 detected genes, data were normalized using the *sctransform* and *glmGamPoi* packages (26). Twenty principal components were selected for UMAP projection and clustering using the *Seurat* implementation of the Leiden algorithm with resolution set at 0.5. Cell type annotations were provided in the original publication.

Abbas *et al*. (27)

scRNA-seq data of eight patients with relapsed/refractory AML treated with azacitidine and nivolumab were analyzed as previously published. A total of 60,753 AML and 52,641 TME cells from 22 BM aspirates (8 pre-ICB and 14 post-ICB) were used for downstream analyses. IES scores were compared for responders (PT1A/2A/3A) and non-responders (PT4A/5A/6A) before (timepoint A) and after treatment (timepoints B and C) across cell types. Cell type annotations were provided in the original publication.

Tirosh *et al*. (28)

scRNA-seq profiles of 19 human melanoma tumors were downloaded from GEO: GSE77940. After filtering out cells with >5% mitochondrial gene counts, or <200 or >2,500 detected genes, data were processed as detailed above. Cell type annotations were provided in the original publication.

Sade-Feldman *et al*. (29)

scRNA-seq profiles of 16,291 individual immune cells from 48 tumor samples of patients with melanoma treated with ICB were accessed through the Single Cell Portal (https://singlecell.broadinstitute.org). Data were processed as detailed above. Cells were

automatically annotated (ENCODE/Blueprint reference map) using the *SingleR* and *celldex* packages in R (30, 31).

Key Resources

Reagent or Resource	Source	Identifier
Antibodies		
CD45 (clone 2D1)	BioLegend	Cat#: 368510
CD19 (clone HIB19)	BioLegend	Cat#: 302258
CD4 (clone OKT4)	BioLegend	Cat#: 317408
CD8 (clone SK1)	BioLegend	Cat#: 344740
CD8 (clone RPA T8)	e-Bioscience	Cat#: 45-0088-42
CD57 (clone HNK1)	BioLegend	Cat#: 359608
KLRG1 (clone SA231A2)	BioLegend	Cat#: 367716
CD28 (clone CD28.2)	BioLegend	Cat#: 302946
CD33 (clone P67.6)	BioLegend	Cat#: 366622
TIGIT (clone A15153G)	BioLegend	Cat#: 372712
CD127 (clone A019D5)	BioLegend	Cat#: 351326
PD1 (clone EH12.1)	BD Biosciences	Cat#: 560795
Tim-3 (clone F38-2E2)	BioLegend	Cat#: 345034
CD34 (clone 561)	BioLegend	Cat#: 343608
CD3 (clone OKT3)	BioLegend	Cat#: 317340
Ki-67(clone B56)	BD Biosciences	Cat#: 561284
γH2AX (clone N1-431)	BD Biosciences	Cat#: 562377
ICOS (clone C398.4)	BioLegend	Cat#: 313534
CD25 (clone BC96)	e-Bioscience	Cat#: 25-0259-42
**Biological Samples**		
Newly diagnosed AML	This paper	GEO: GSE176100
Relapsed/refractory AML	This paper	GEO: GSE178926
Newly diagnosed AML	This paper	Flow cytometry
**Deposited data**		
Human Cell Atlas (HCA) bone marrow dataset	Freeberg and Welter	https://data.humancellatlas.org/explore/projects/cc95ff89-2e68-4a08-a234-480eca21ce79
Human Primary Cell Atlas (HPCA)	Mabbott et al. (2013)	https://rdrr.io/github/LTLA/celldex/src/R/HumanPrimaryCellAtlasData.R
Normal bone marrow NK cells	Yang et al. (2019)	GEO: GSE130430
Newly diagnosed AML	Vadakekolathu et al. (2020)	GEO: GSE134589
Newly diagnosed AML	TCGA-AML	https://www.cbioportal.org/<br>See **Table S1**
Newly diagnosed AML	COG-TARGET AML	https://www.cbioportal.org/<br>See **Table S1**
Newly diagnosed AML	Beat-AML Master Trial	https://www.cbioportal.org/<br>See **Table S1**
Newly diagnosed AML	Herold et al. (2018)<br>Li et al. (2013)	GEO: GSE37642
Newly diagnosed AML	Ng et al. (2016)	GEO: GSE76004
Newly diagnosed AML (scRNA-seq)	van Galen et al. (2019)	GEO: GSE116256<br>DOI: 10.1016/j.cell.2019.01.031
Newly diagnosed AML (scRNA-seq)	FIMM<br>Dufva et al. (2020)	syn21991338<br>DOI: 10.1016/j.ccell.2020.06.002
AML immunotherapy (scRNA-seq)	Abbas et al. (2021)	https://ega-archive.org/studies/EGAS00001004894<br>DOI: 10.1038/s41467-021-26282-z
Cutaneous melanoma immunotherapy (scRNA-seq)	Sade-Feldman et al. (2018)	GEO: GSE120575<br>DOI: https://doi.org/10.1016/j.cell.2018.10.038
Cutaneous melanoma (scRNA-seq)	Tirosh et al. (2016)	GEO: GSE77940<br>DOI: 10.1126/science.aad0501

Cutaneous melanoma	TCGA PanCancer Atlas<br>Gao et al. (2013)	https://www.cbioportal.org/
Cutaneous melanoma immunotherapy	Gide et al. (2019)	ENA: PRJEB23709<br>See Table S9
Cutaneous melanoma immunotherapy	Prat et al. (2017)	GEO: GSE93157<br>See Table S10
Cutaneous melanoma	Bagaev et al. (2021)	DOI: 10.1016/j.ccell.2021.04.014<br>https://science.bostongene.com/tumor-portrait/
**Chemicals, Peptides, and Recombinant Proteins**		
IL3	PHC0034	Life Technologies
G-CSF	RGCSF10	Life Technologies
GM-CSF	PHC2013	Life Technologies
SCF	PHC2115	Life Technologies
Critical Commercial Assays		
RNA extraction kit	Qiagen	Cat#: 74106
PanCancer Immune Profiling kit	NanoString Technologies	Cat#: 115000132
Qubit™ RNA HS Assay Kit	Thermo Fisher Scientific	Cat#: Q32852
RNA Clean & Concentrator-5 with DNase I Set	Zymo Research	Cat#: R1013
**Software and Algorithms**		
celldex R package	Aran et al. (2019)	https://bioconductor.org/packages/release/data/experiment/html/celldex.html
CIBERSORT	Gentles et al. (2015)	https://cibersort.stanford.edu/
clusterProfiler R package	Yu et al. (2012)	https://guangchuangyu.github.io/software/clusterProfiler/
ClustVis	Metsalu et al. (2015)	https://biit.cs.ut.ee/clustvis/
corrplot R package	Wei et al. (2021)	https://github.com/taiyun/corrplot
EnhancedVolcano R package	Blighe et al. (2019)	DOI: 10.18129/B9.bioc.EnhancedVolcano
EPIC	Racle et al. (2017)	http://epic.gfellerlab.org
GeneMANIA	Warde-Farley et al. (2010)	https://genemania.org/
GEPIA2021	Chenwei et al. (2021)	http://gepia2021.cancer-pku.cn/sub-expression.html
GEPIA2	Tang et al. (2019)	http://gepia2.cancer-pku.cn/#index
ggfortify R package	Horikoshi et al. (2018)	https://CRAN.R-project.org/package=ggfortify
ggplot2 R package	Wickham (2016)	https://ggplot2.tidyverseorg
ggrepel R package	Slowikowski et al. (2021)	https://cran.r-project.org/web/packages/ggrepel/index.html
glmnet R package	Friedman et al. (2010)	https://www.jstatsoft.org/v33/i01/
GSEA-P	Subramanian et al. (2007)	https://www.gsea-msigdb.org/gsea/downloads.jsp
Genotype-Tissue Expression (GTEx)	Broad Institute of MIT and Harvard	https://gtexportal.org/home/
ggVennDiagram R package	Gao et al. (2021)	https://cran.r-project.org/web/packages/ggVennDiagram/readme/README.html
GOSemSim R package	Yu et al. (2010)	http://bioconductor.org/packages/release/bioc/html/GOSemSim.html

immunedeconv R package	Sturm et al. (2019)	https://github.com/icbi-lab/immunedeconv
ImmuneSigDB	Godec et al. (2016)	http://www.gsea-msigdb.org/gsea/msigdb/genesets.jsp?collection=IMMUNESIGDB
maxstat R package	Hothorn et al. (2003)<br>Lausen et al. (2004)	https://cran.r-project.org/web/packages/maxstat/maxstat.pdf
nCounter advanced analysis v2.0.134	NanoString Technologies	https://www.nanostring.com/products/analysis-solutions/ncounter-advanced-analysis-software/
NetworkAnalyst	Xia et al. (2015)	https://www.networkanalyst.ca/
nSolver v4.0.70	NanoString Technologies	https://www.nanostring.com/products/analysis-solutions/ncounter-advanced-analysis-software/
PANTHER (v16.0)	Mi et al. (2013)<br>Thomas et al. (2003)	http://www.pantherdb.org/
pathfindR R package	Ulgen et al. (2019)	https://cran.r-project.org/web/packages/pathfindR/index.html
Prism v9.0	GraphPad	https://www.graphpad.com/scientific-software/prism/
quanTIseq	Finotello et al. (2019)	https://icbi.i-med.ac.at/software/quantiseq/doc/
R v4.0.4	R Core Team	https://cran.r-project.org/bin/macosx/
sctransform R package	Hafemeister and Satija, 2019	https://github.com/ChristophH/sctransform
SeneQuest	Gorgoulis et al. (2021)	https://senequest.net/
Seurat R package (v4)	Hao et al. (2021)	https://github.com/satijalab/seurat
SingleR R package	Aran et al. (2019)	https://bioconductor.org/packages/release/bioc/html/SingleR.html
SPSS Statistics v26	IBM	https://www.ibm.com/uk-en/analytics/spss-statistics-software
STRING	Szklarczyk et al. (2019)	http://string-db.org
survminer R package	Kassambara et al. (2021)	https://cran.r-project.org/web/packages/survminer/survminer.pdf
SVA Bioconductor package	Leek et al. (2021)	https://bioconductor.org/packages/sva/
TIDE	Jiang et al. (2018)	http://tide.dfci.harvard.edu/login/
TIMER2.0	Taiwen et al. (2020)	http://timer.cistrome.org/
TRRUST	Han et al. (2018)	https://www.grnpedia.org/trrust/
UCell R package	Andreatta et al. (2021)	https://github.com/carmonalab/UCell
UCSC Xena	Goldman et al. (2020)	https://xenabrowser.net/datapages/

Supplementary Figures

Patients/samples analyzed in this study
N=2,461/2,673

***In silico* cohorts (AML)**
(N=1,730/1,833)

TCGA-AML	N=147/147 (Bulk RNA-sequencing)
Beat-AML	N=264/277 (Bulk RNA-sequencing)
COG-TARGET AML	N=145/176 (Bulk RNA-sequencing)
FIMM AML	N=8/8 (Single-cell RNA-sequencing)
van Galen *et al.*	N=16/35 (Single-cell RNA-sequencing)
Abbas *et al.* (ICB)	N=8/22 (Single-cell RNA-sequencing)
PMCC (GSE134589)	N=290 (NanoString)
CHOP (GSE134589)	N=40/66 (NanoString)
GSE106291	N=250 (Bulk RNA-sequencing)
GSE37642-GPL96	N=422 (Affymetrix)
GSE37642-GPL570	N=140 (Affymetrix)

Wet-lab AML cohorts
(N=166/259)

SAL (GSE176100)	N=64/131 (NanoString)
JHU1	N=43 (Flow cytometry, mechanistic studies)
JHU2 (GSE176100)	N=26/52 (NanoString)
Azacitidine + pembrolizumab (NCT02845297; GSE178926)	N=33 (NanoString)

***In silico* cohorts (cutaneous melanoma)**
(N=565/581)

TCGA Pan-Cancer Atlas	N=441 (Bulk RNA-sequencing)
PRJEB23709 (ICB)	N=73 (Bulk RNA-sequencing)
Tirosh *et al.* (GSE77940)	N=19 (Single-cell RNA-sequencing)
Sade-Feldman *et al.* (ICB)	N=32/48 (Single-cell RNA-sequencing)

Figure S1 | Patient cohorts and gene expression platforms utilized in this study. Related to Materials & Methods.

PMCC = Princess Margaret Cancer Centre; CHOP = Children's Hospital of Philadelphia; SAL = Studien Allianz Leukämie; JHU = Johns Hopkins University; ICB = immune checkpoint blockade.

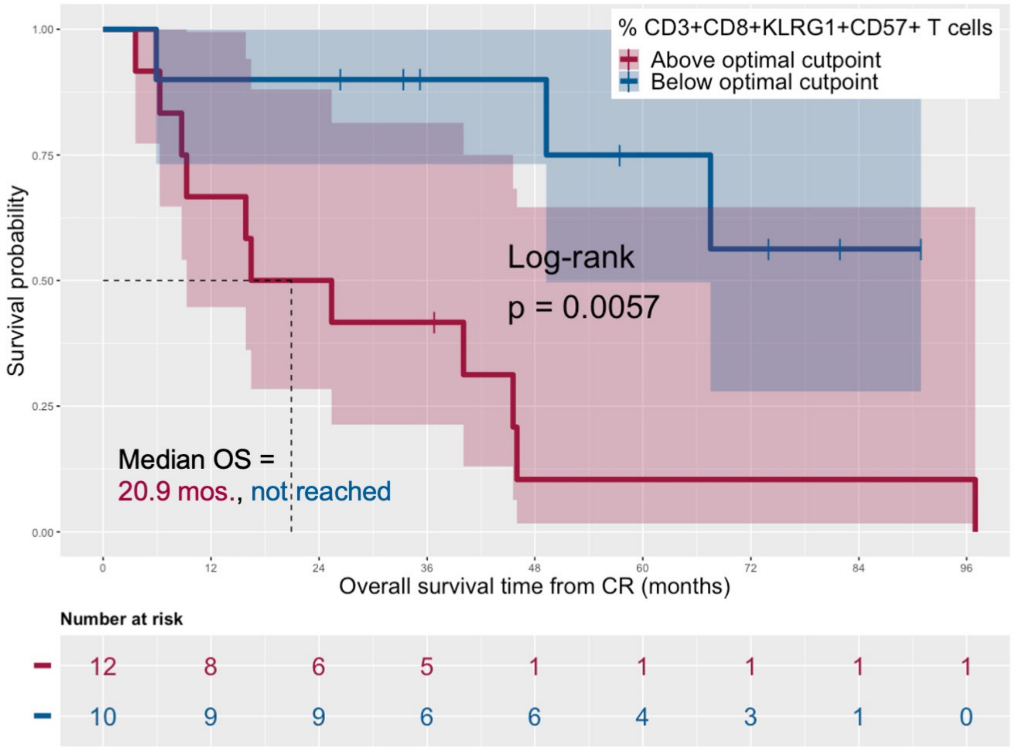

Figure S2 | Senescent-like T cells at time of response assessment and survival in patients with AML in the JHU1 cohort. Related to Fig. 2.

Kaplan-Meier estimates of overall survival (OS) from complete remission (CR) in patients (N = 22) with senescent-like $CD3^{+}CD8^{+}KLRG1^{+}CD57^{+}$ T cells above (magenta line) and below (blue line) the optimal cut-point, which was computed using the *maxstat* package in R. Survival curves were compared using a log-rank test (*survminer* package in R). Median OS is indicated (color-coded by the optimal cut-point of the proportion of $CD3^{+}CD8^{+}CD57^{+}KLRG1^{+}$ T cells). HR = hazard ratio; CI = confidence interval.

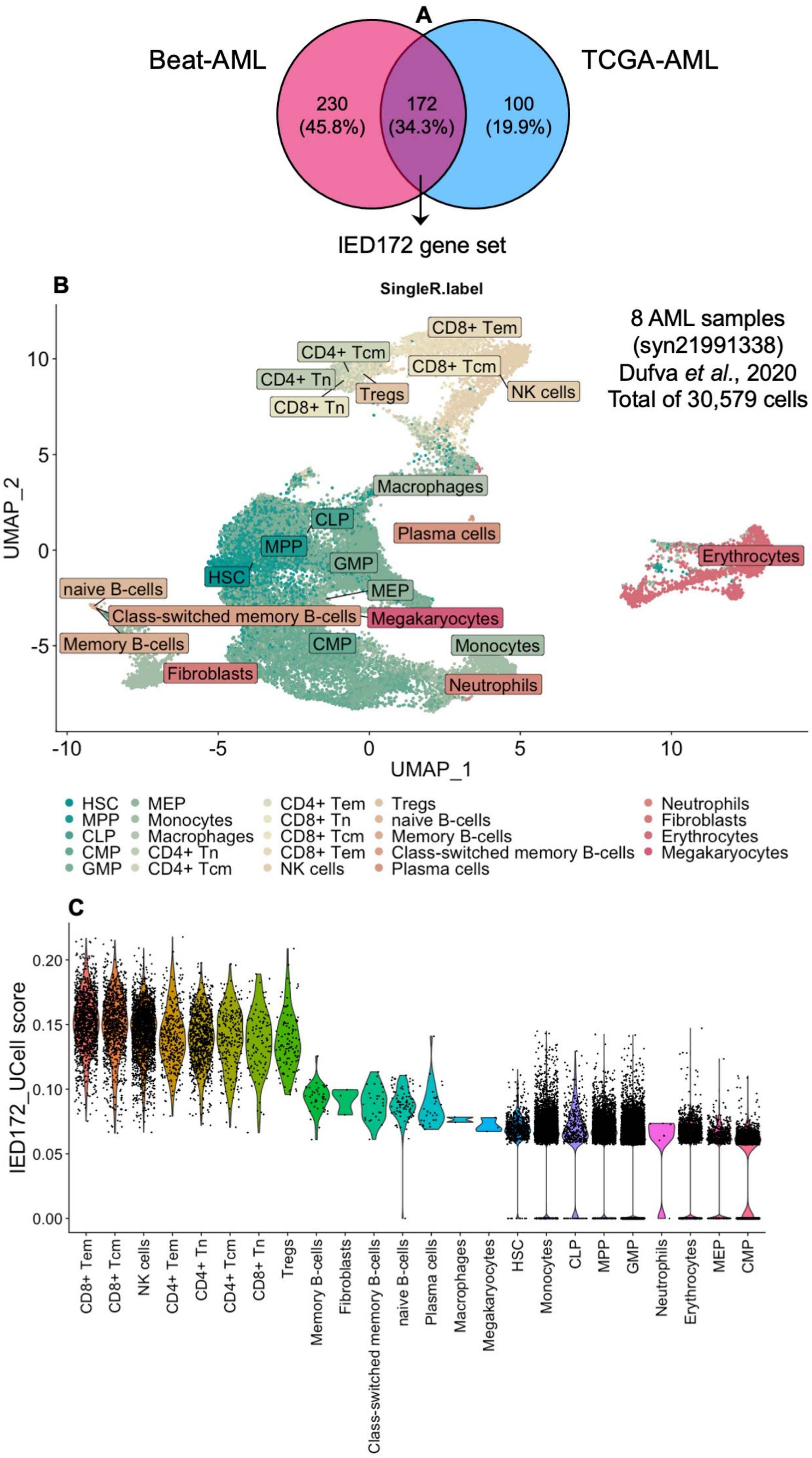

Figure S3 | Expression of IED genes in FIMM AML cases (single-cell RNA-sequencing). Related to Fig. 2.

(A) Venn diagram showing the overlap between immunosenescence-associated genes in TCGA-AML and Beat-AML Master Trial cases and the derivation of a 172-gene IED signature.
(B) Uniform manifold approximation and projection (UMAP) embedding of cell types identified by *SingleR* (ENCODE/Blueprint annotation) in 8 BM samples from patients with untreated AML (FIMM cohort) (18).
(C) Violin plot of the expression of IED172 genes in the FIMM cohort. The IED172 score was computed using the *UCell* package in R.

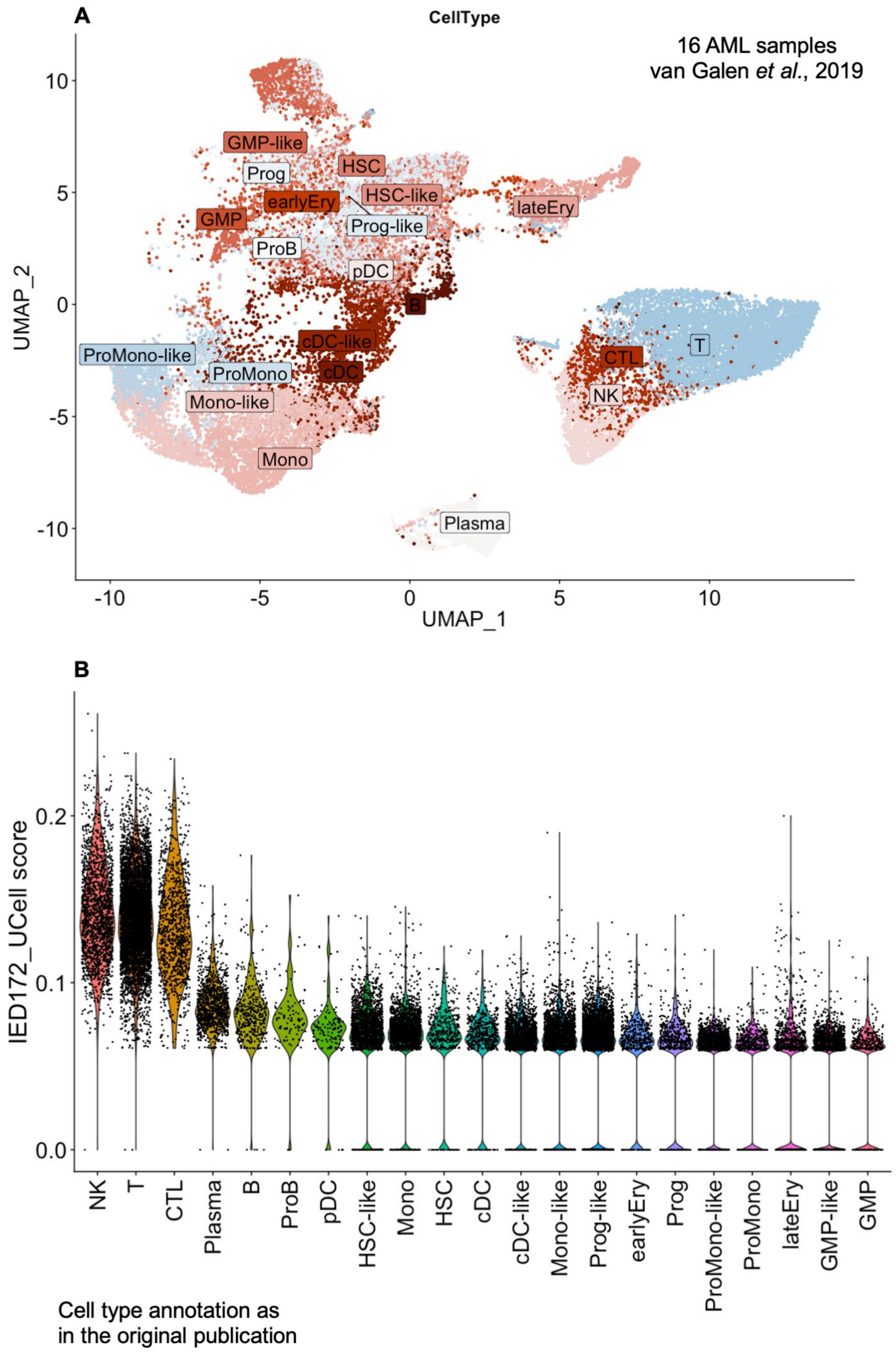

Figure S4 | Expression of IED genes in AML cases from van Galen et al. (single-cell RNA-sequencing). Related to Fig. 2.

(A) Uniform manifold approximation and projection (UMAP) embedding of cell types identified by van Galen *et al*. in 16 BM samples from patients with untreated AML (25).
(B) Violin plot of the expression of IED172 genes in the van Galen cohort. The IED172 score was computed using the *UCell* package in R. Cell type annotation as in the original publication.

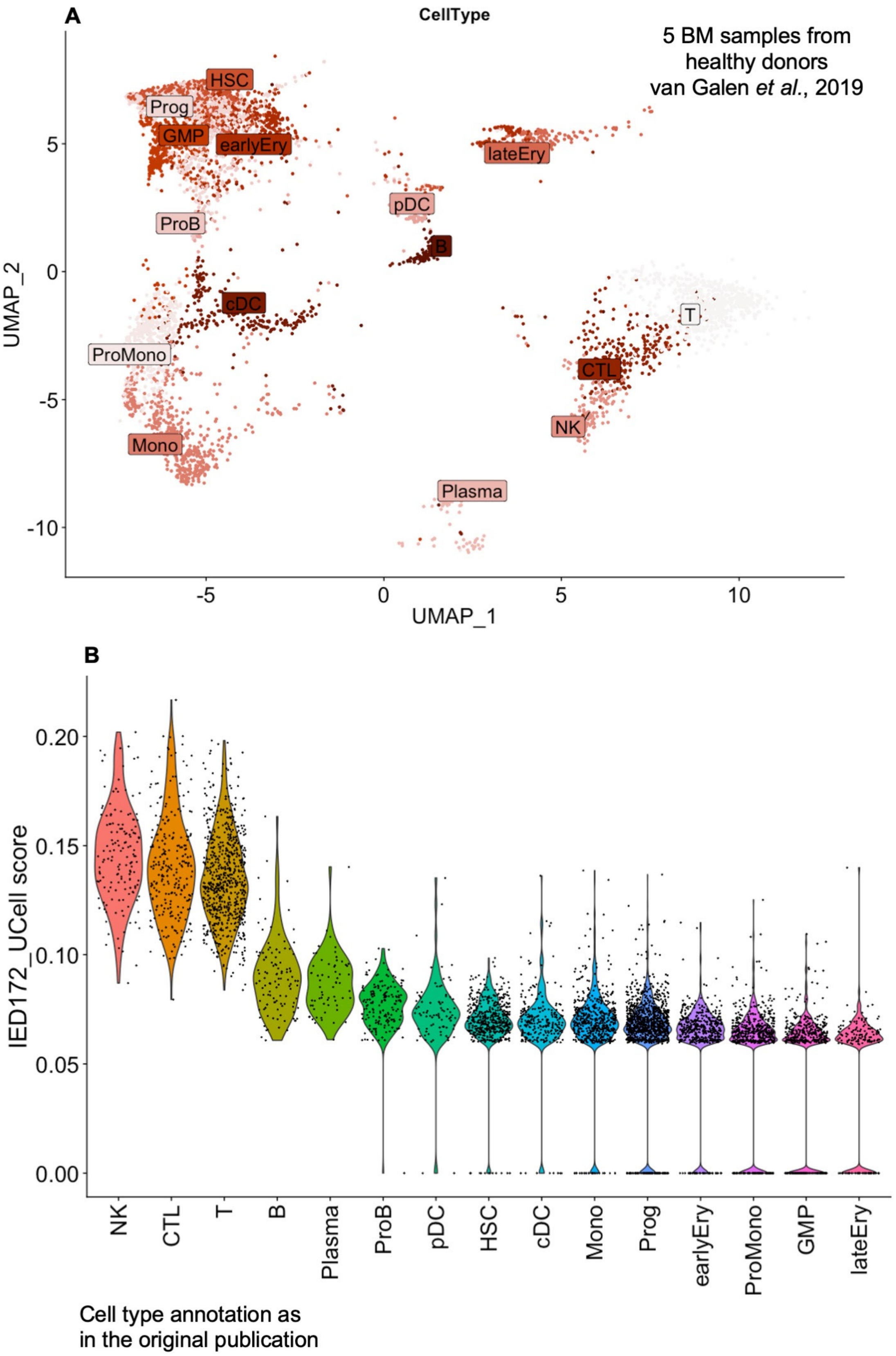

Figure S5 | Expression of IED genes in healthy donor BM samples from van Galen et al. (single-cell RNA-sequencing). Related to Fig. 2.

(A) Uniform manifold approximation and projection (UMAP) embedding of cell types identified by van Galen *et al*. in 5 BM samples from healthy controls (25).

(B) Violin plot of the expression of IED172 genes in healthy donor BM samples from the van Galen cohort. The IED172 score was computed using the *UCell* package in R. Cell type annotation as in the original publication.

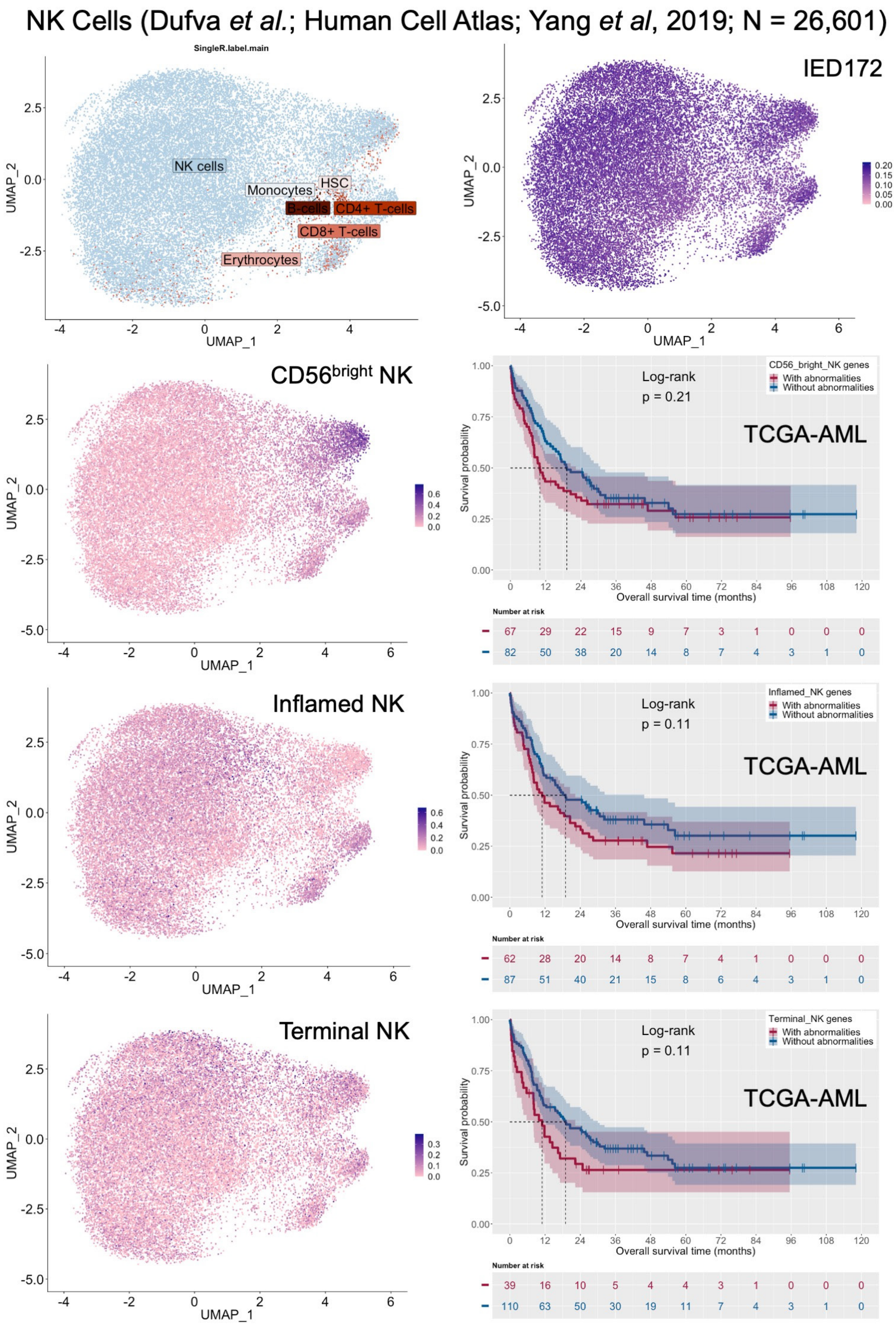

Figure S6 | Expression of IED genes by natural killer (NK) cell functional subtypes from patients with AML (FIMM cohort). Related to Fig. 2.

Uniform manifold approximation and projection (UMAP) embedding of IED172 genes in NK cell subsets from healthy donor BM samples and patients with AML from the FIMM cohort (18). The NK functional scores were computed using the *UCell* package in R. A list of marker genes is provided in Supplementary Table 3 and can also be retrieved through the original publication (24).

Abnormalities of the top 15 genes defining each NK subtype (mostly RNA up-regulation and/or gene amplification) were correlated with clinical outcomes in TCGA-AML cases (right column). Survival curves were compared using a log-rank test (*survminer* package in R).

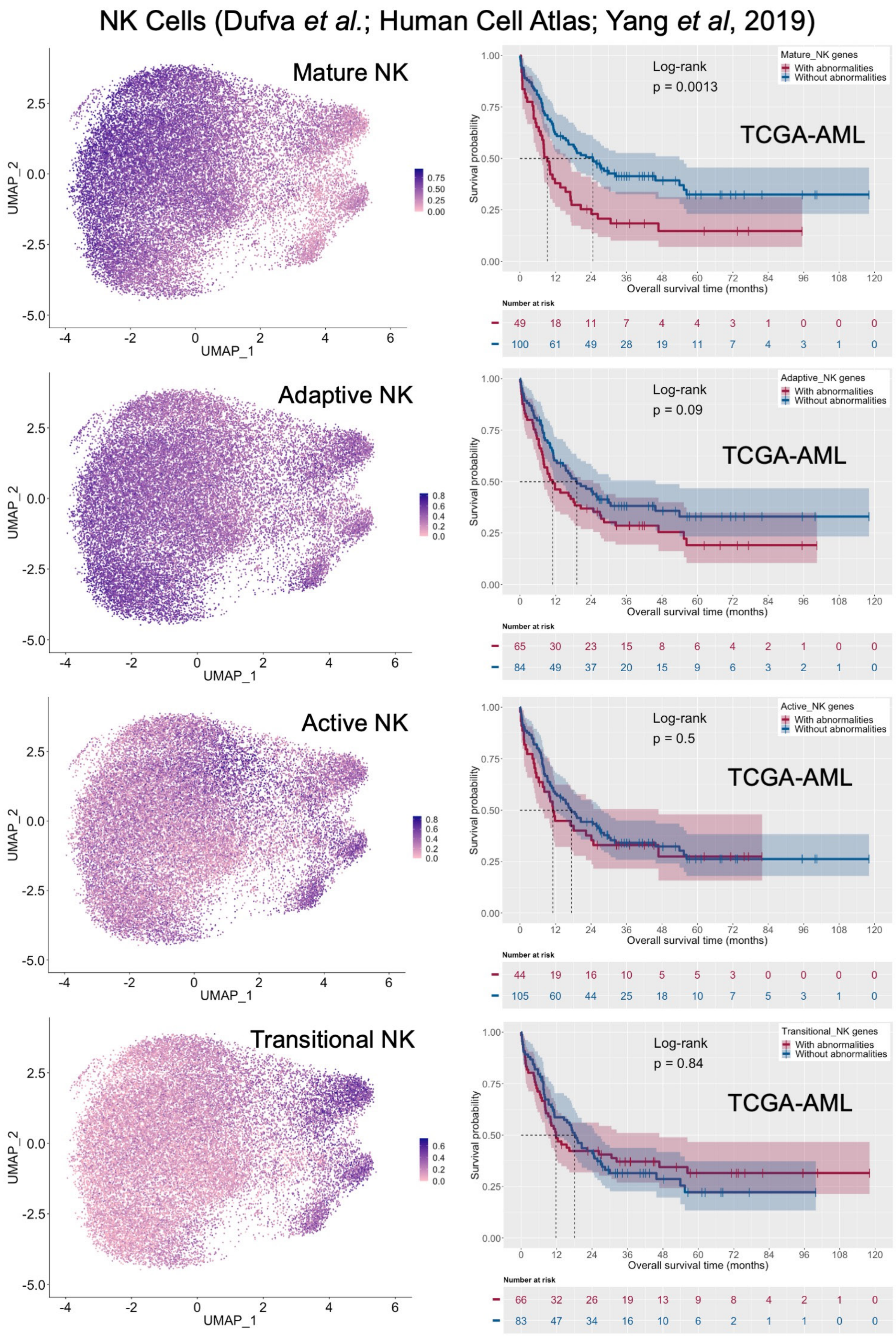

Figure S7 | Expression of IED genes by natural killer (NK) cell functional subtypes from patients with AML (FIMM cohort). Related to Fig. 2.

Uniform manifold approximation and projection (UMAP) embedding of IED172 genes in NK cell subsets from healthy donor BM samples (24) and patients with AML from the FIMM cohort (18). The NK functional scores were computed using the *UCell* package in R. A list of marker genes is provided in Supplementary Table 3 and can also be retrieved through the original publication (24).

Abnormalities of the top 15 genes defining each NK subtype (mostly RNA up-regulation and/or gene amplification) were correlated with clinical outcomes in TCGA-AML cases (right column). Survival curves were compared using a log-rank test (*survminer* package in R).

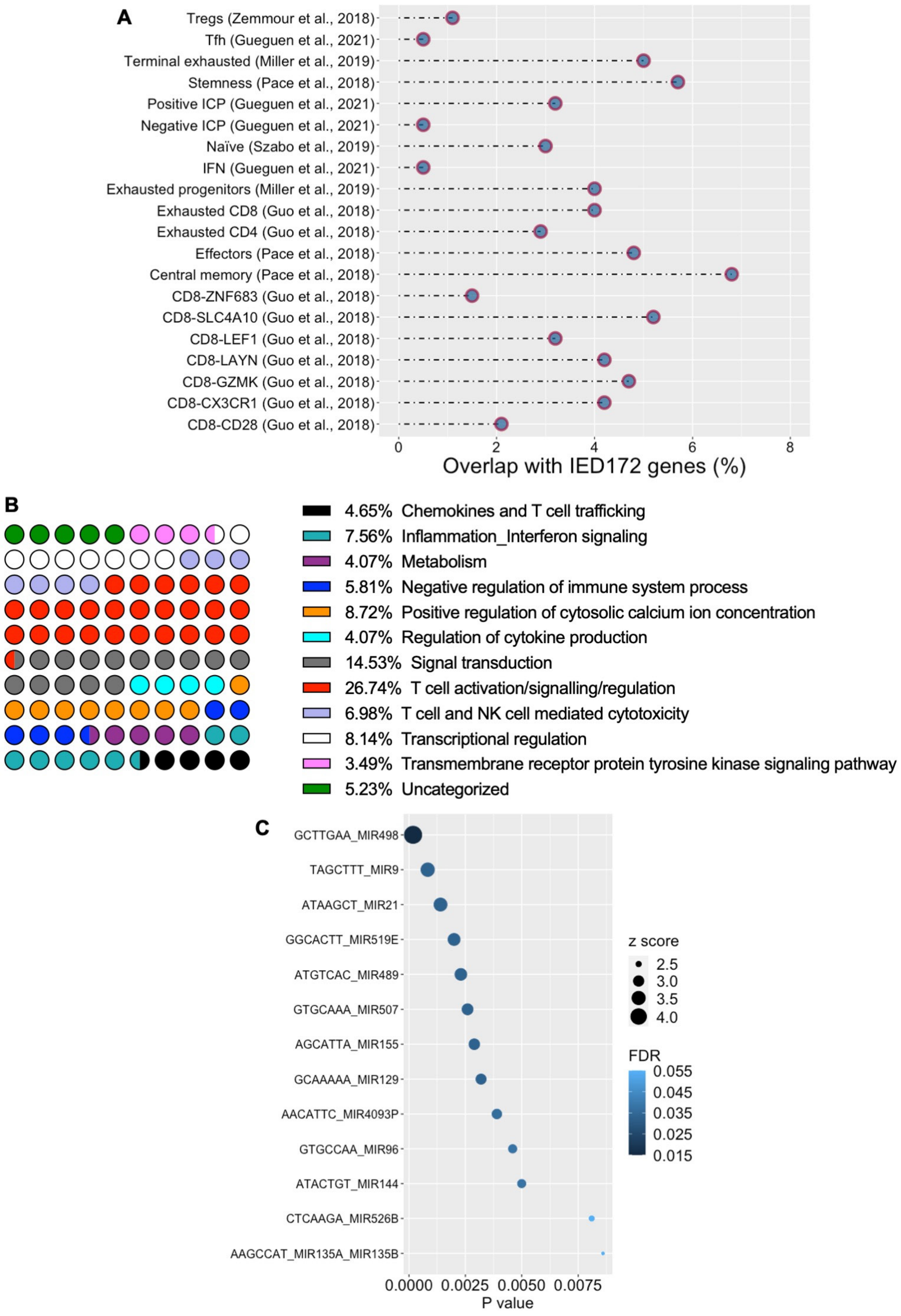

Figure S8 | Signature overlap and pathway analysis. Related to Figure 2.

(A) Percent overlap between the 172 immune effector dysfunction (IED) genes from this study and published signatures of T-cell dysfunction/exhaustion and response of solid tumors to immune checkpoint blockade (32-34). Gene lists are provided in Supplementary Table 4.
(B) Gene ontologies (GO) captured by the 172 genes in the IED signature (Panther Classification System, v16.0; http://www.pantherdb.org/). Percent of gene hits against total number of process hits. Parts of whole (100%) are shown as color-coded entries.
(C) Bubble plot depicting enriched miRNAs in IED172 signature genes (*clusterProfiler* package in R).

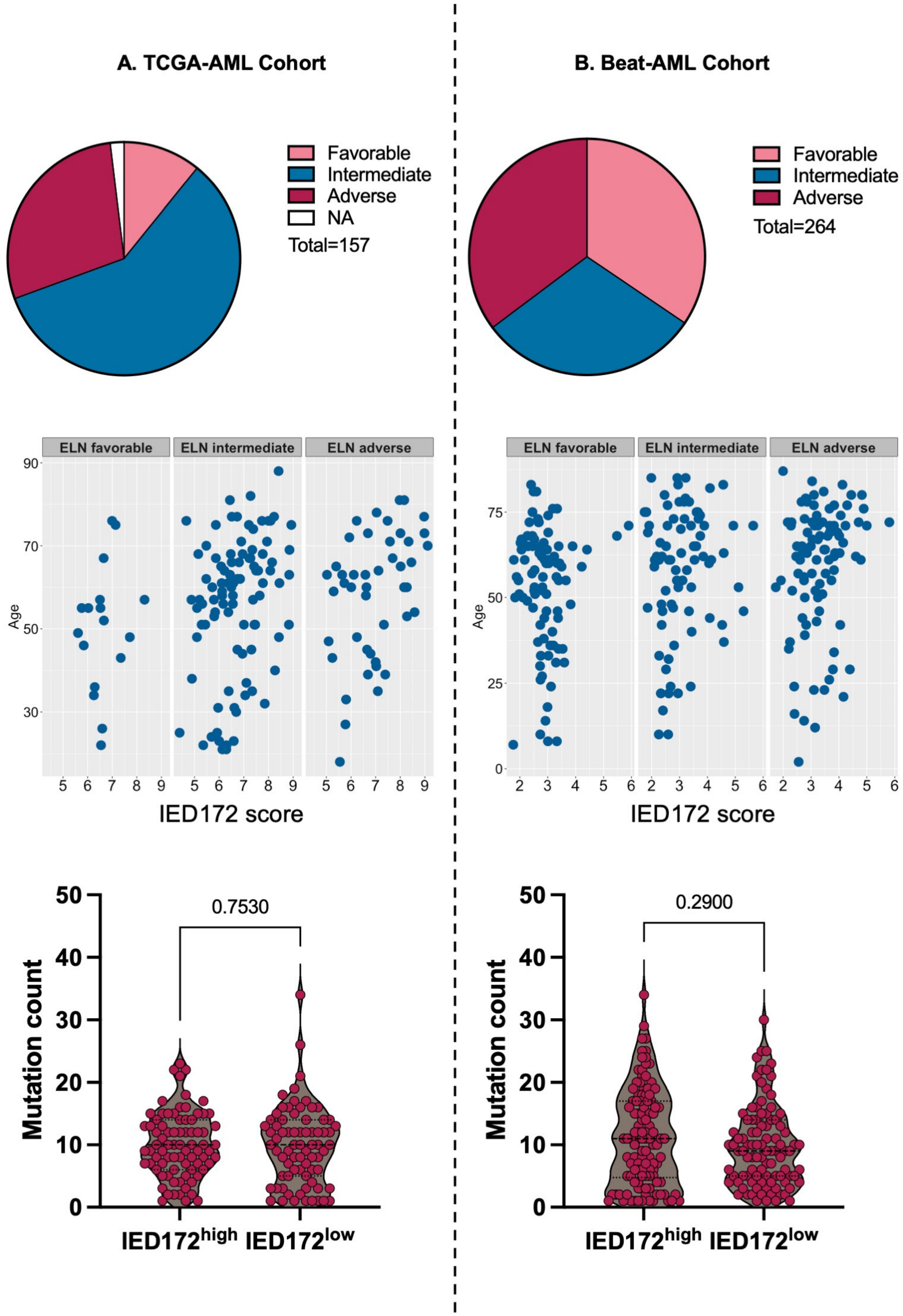

Figure S9 | IED172 scores in TCGA-AML and Beat-AML cases. Related to Figure 2.

(A) European Leukemia Net (ELN) risk category, correlation between IED172 score and patient age, and mutation count in TCGA-AML cases (Mann-Whitney *U* test for unpaired determinations).

(B) European Leukemia Net (ELN) risk category, correlation between IED172 score and patient age, and mutation count in Beat-AML cases (Mann-Whitney *U* test for unpaired determinations).

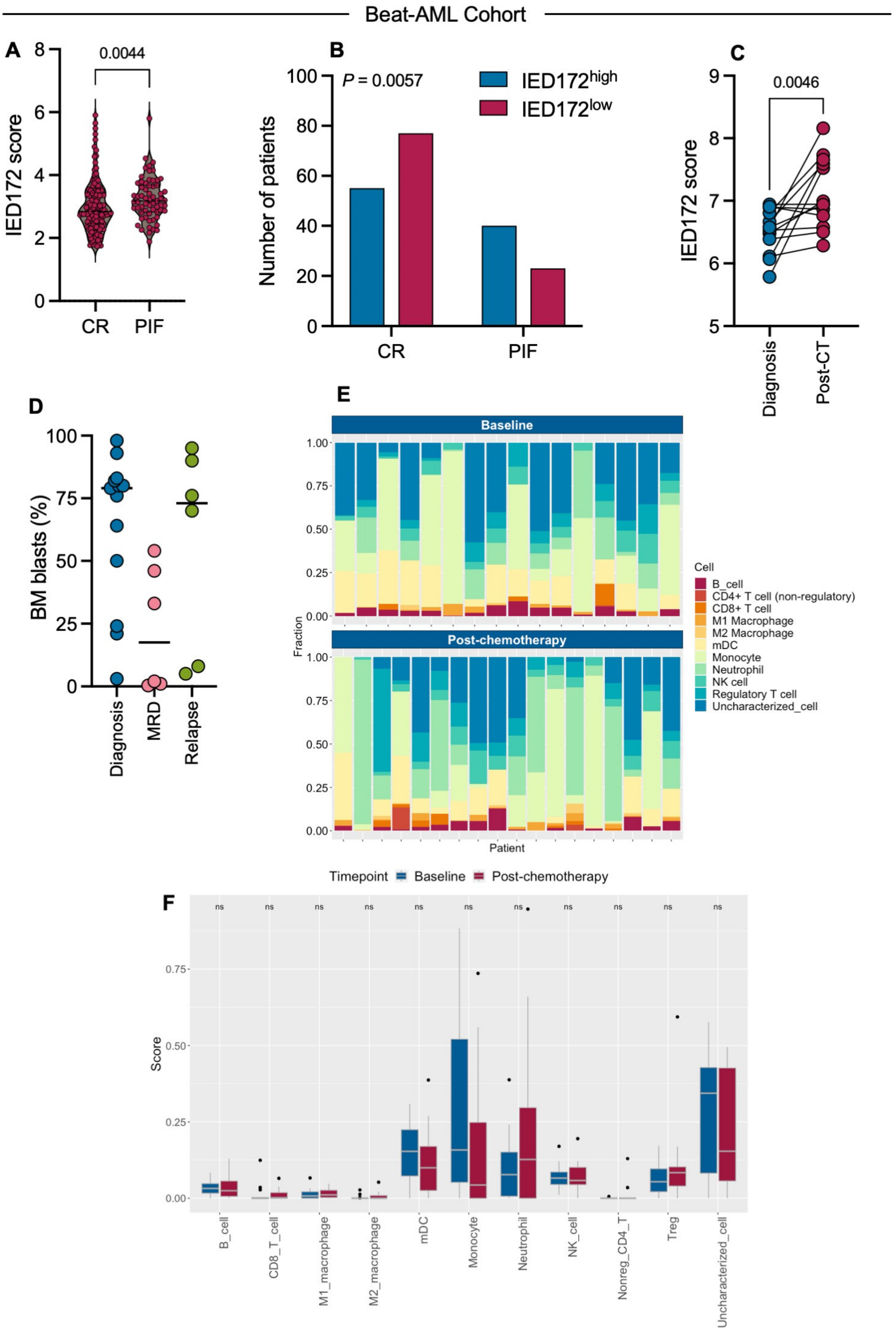

Figure S10 | IED172 scores in Beat-AML cases. Related to Figure 2.

(A) IED172 scores at baseline in Beat-AML patients who experienced primary induction failure (PIF, following a standard 2 cycles) and in those who achieved complete remission (CR) after induction chemotherapy.

(B) Number of patients with PIF and CR in the $IED172^{high}$ and $IED172^{low}$ group at baseline (median split). Data were compared using the Fisher's exact test.

(C) IED172 scores at baseline and at time of response assessment in 13 cases with matched bone marrow (BM) samples. Data were compared using the Wilcoxon matched pairs signed rank test. CT = chemotherapy.

(D) Percentage of BM blasts in 13 cases with matched samples. MRD = measurable residual disease at time of BM sampling.

(E) Immune cell type deconvolution of bulk RNA-seq data. The composition of immune cells in the tumor microenvironment was inferred using quanTIseq (*immunedeconv* package in R), which provides an absolute score representing immune cell fractions and allows both intra- and inter-sample comparisons (15).

(F) Boxplots comparing immune cell fractions in matched BM samples collected at baseline and post-chemotherapy (Mann-Whitney *U* test for unpaired determinations). Outliers are shown with black dots. **** $P < 0.0001$; *** $P < 0.001$; * $P < 0.05$; ns = not significant; mDC = myeloid dendritic cells.

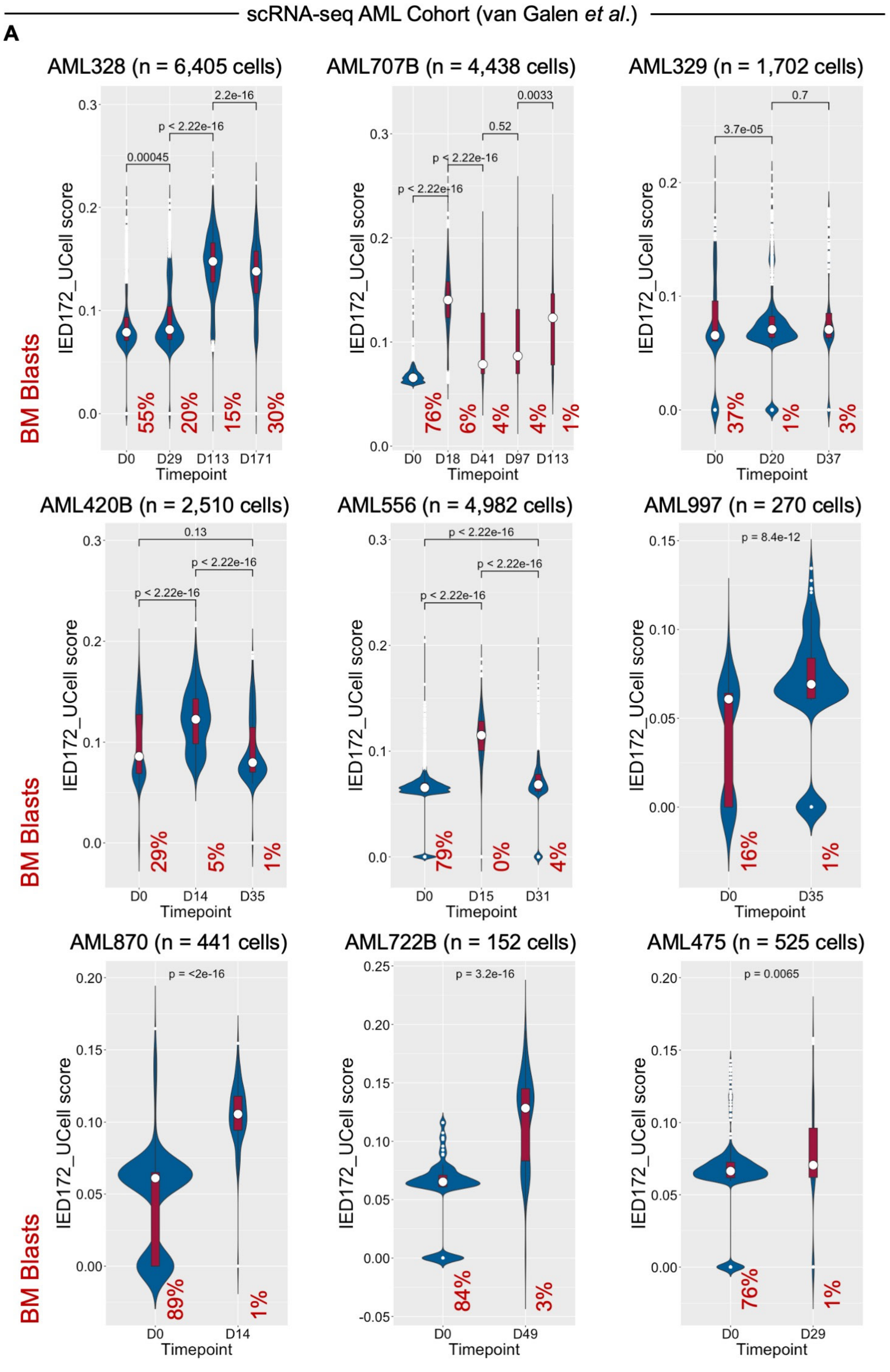

Figure S11A | IED172 scores in AML cases from van Galen et al. Related to Figure 3.

Violin plot of the expression of IED172 genes in patients with AML from the van Galen cohort (25). The IED172 score was computed using the *UCell* package in R. Blast counts at diagnosis and at different timepoints after treatment are indicated in each plot. BM = bone marrow; D = day.

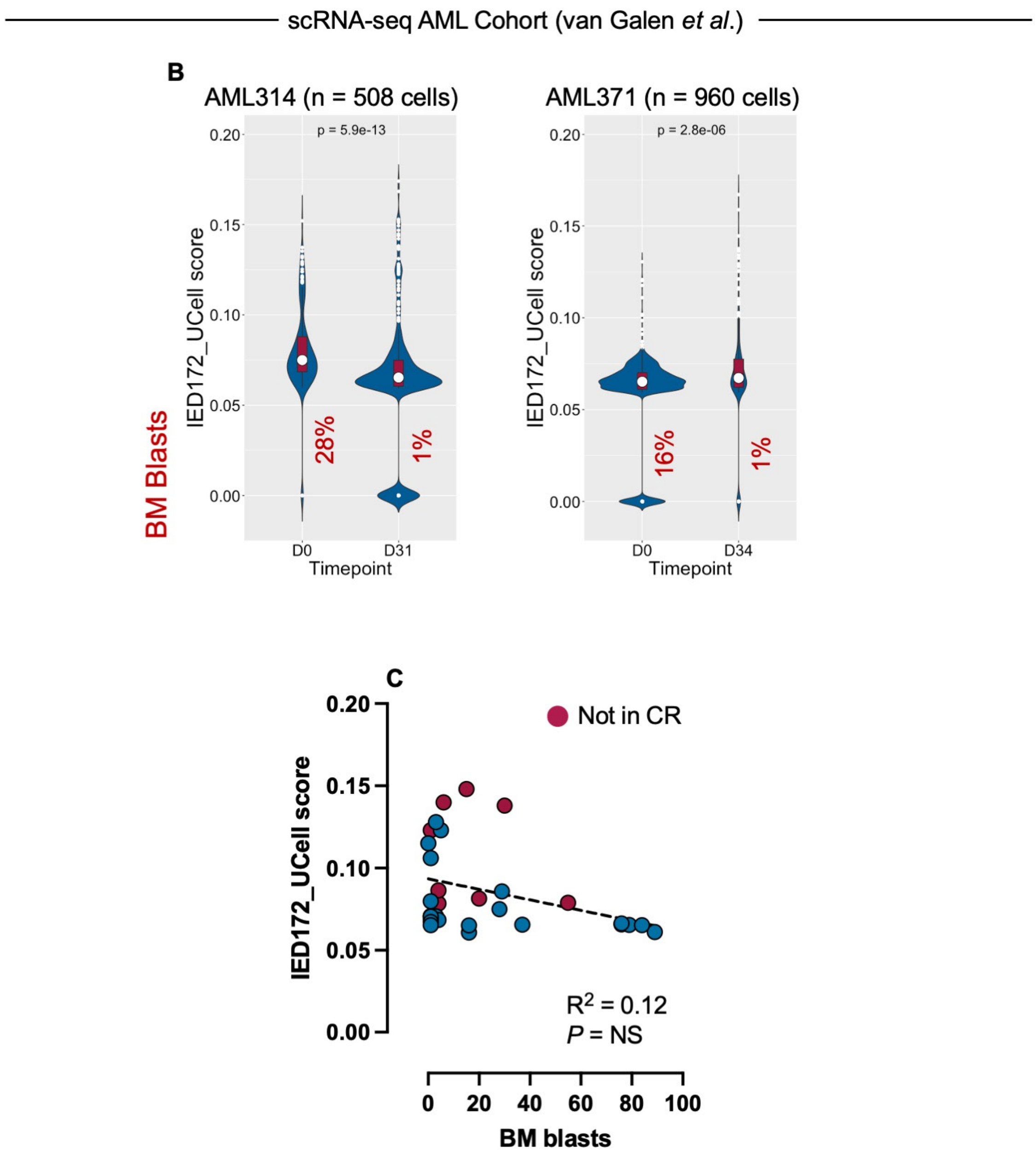

Figure S11B-S11C | IED172 scores in AML cases from van Galen et al. Related to Figure 3.

(B) Violin plot of the expression of IED172 genes in patients with AML from the van Galen cohort (25). The IED172 score was computed using the *UCell* package in R. Blast counts at diagnosis and at different timepoints after treatment are indicated in each plot. BM = bone marrow; D = day.

(C) Correlation between blast count and IED172 score. Red dots denote BM aspirates collected from patients who were not in complete remission (CR) at time of sampling.

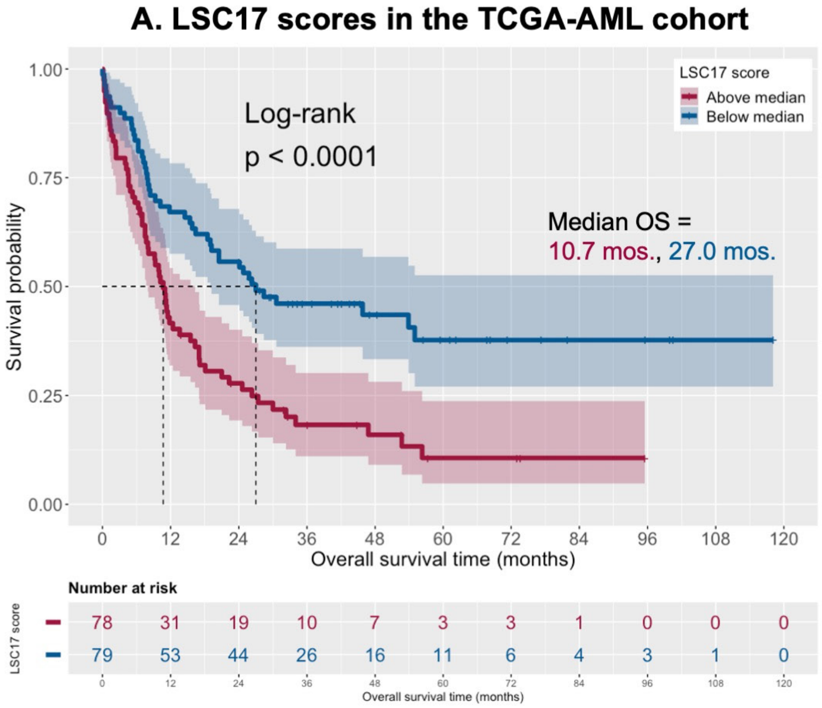

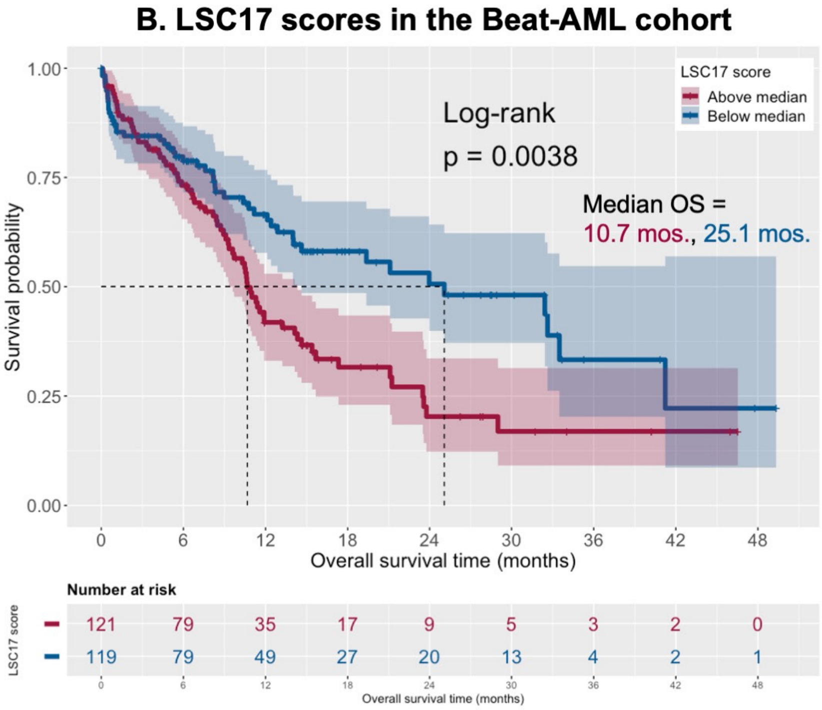

Figure S12 | Leukemia stem cell (LSC17) score and survival in the TCGA-AML and Beat-AML Master Trial cohorts. Related to Figure 3. The LSC17 score was calculated as the weighted sum of the normalized expression values of the 17 genes included in the signature using the same weights as those provided in the original publication (12). Kaplan-Meier estimates of overall survival (OS) in TCGA-AML (A) and Beat-AML Master Trial patients (B) with above median (magenta line) and below median (blue line) LSC17 scores. Survival curves were compared using a log-rank test (*survminer* package in R).

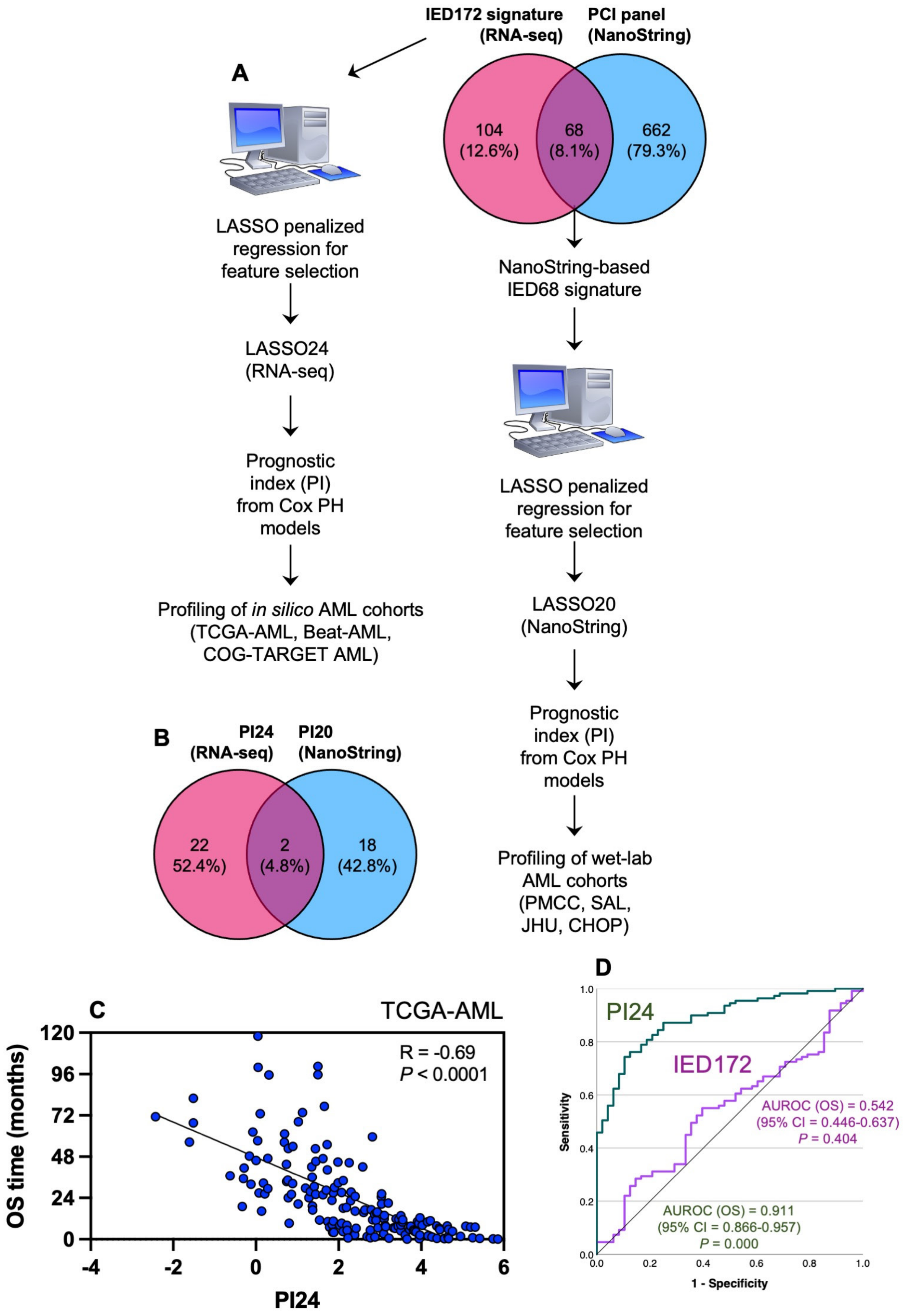
A
IED172 signature (RNA-seq)
PCI panel (NanoString)
104 (12.6%)
68 (8.1%)
662 (79.3%)
LASSO penalized regression for feature selection
LASSO24 (RNA-seq)
Prognostic index (PI) from Cox PH models
Profiling of *in silico* AML cohorts (TCGA-AML, Beat-AML, COG-TARGET AML)
NanoString-based IED68 signature
LASSO penalized regression for feature selection
LASSO20 (NanoString)
Prognostic index (PI) from Cox PH models
Profiling of wet-lab AML cohorts (PMCC, SAL, JHU, CHOP)
B
PI24 (RNA-seq)
PI20 (NanoString)
22 52.4%)
2 (4.8%)
18 (42.8%)
C
TCGA-AML
R = -0.69
P < 0.0001
OS time (months)
PI24
D
PI24
IED172
AUROC (OS) = 0.542
(95% CI = 0.446-0.637)
P = 0.404
AUROC (OS) = 0.911
(95% CI = 0.866-0.957)
P = 0.000
Sensitivity
1 - Specificity

Figure S13 | Derivation of a parsimonious immune effector dysfunction (IED) gene set using LASSO penalized regression for feature selection. Related to Figure 4.
(A) A prognostic index (PI) was generated using β values from Cox regression analyses of gene expression and patient survival, as previously published (35). PCI = PanCancer Immune profiling panel.
(B) Venn diagram showing the overlap between PI24 and PI20 genes.
(C) Correlation between the PI24 score and overall survival (OS) time in the TCGA-AML cohort. R = Pearson correlation coefficient.
(D) AUROC curve measuring the predictive ability of IED172 and PI24 genes for OS. CI = confidence interval. AUROC = 1.0 denotes perfect prediction and AUROC = 0.5 denotes no predictive ability.

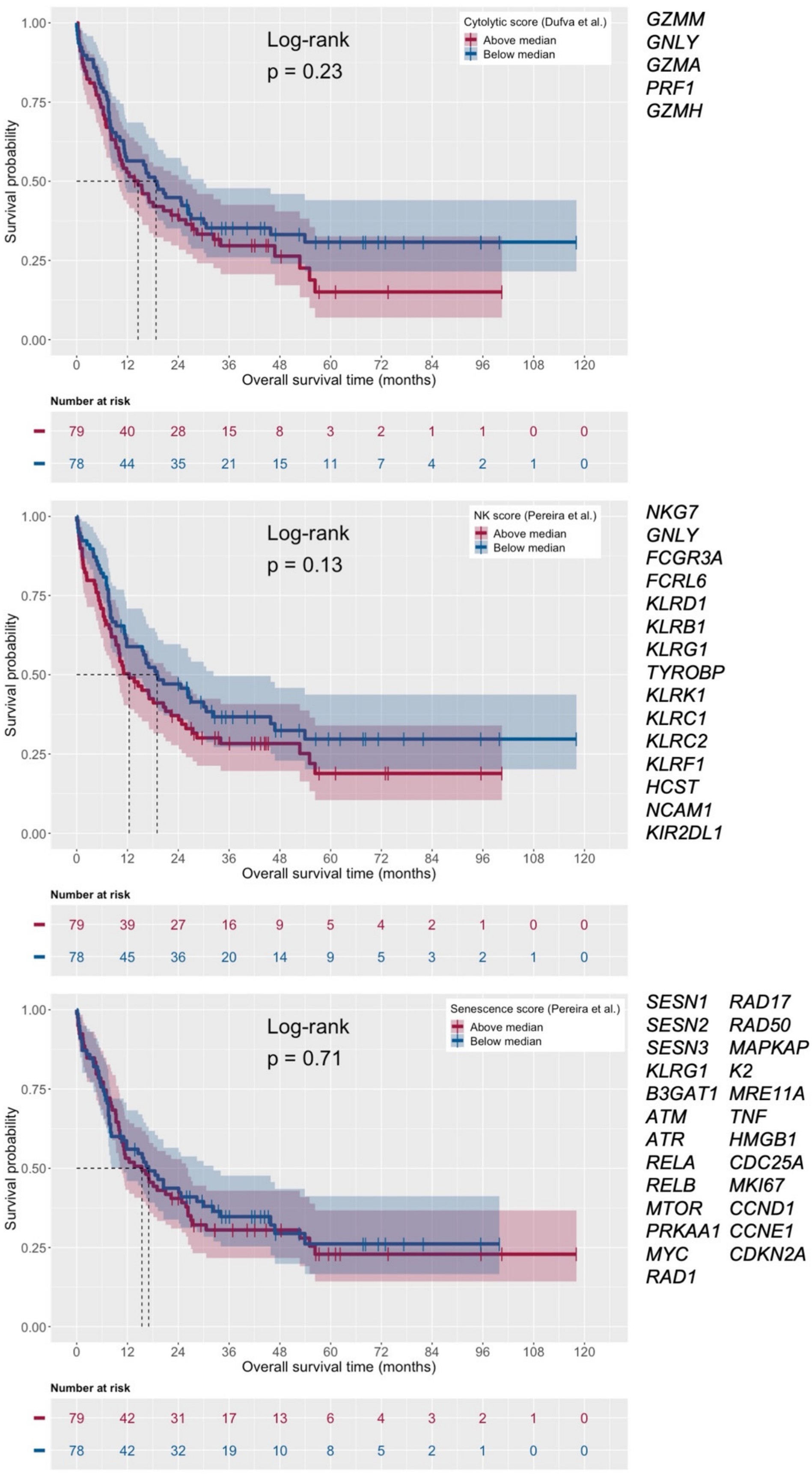

Figure S14 | Published gene sets capturing NK cells, cytolytic activity and immune senescence, and survival in TCGA-AML cases. Related to Figure 4.

Gene lists were downloaded from Dufva *et al*. (cytolytic genes) and Pereira *et al.* (NK-related genes, senescence-related genes) (18, 36) and are reported in the Figure as well as in Supplementary Table 4. For each sample, gene expression scores were calculated as an average (arithmetic mean) of gene expression values for all genes in the signature. Kaplan-Meier estimates of overall survival (OS) in TCGA-AML cases with gene expression score above median (magenta line) and below median (blue line). Survival curves were compared using a log-rank test (*survminer* package in R).

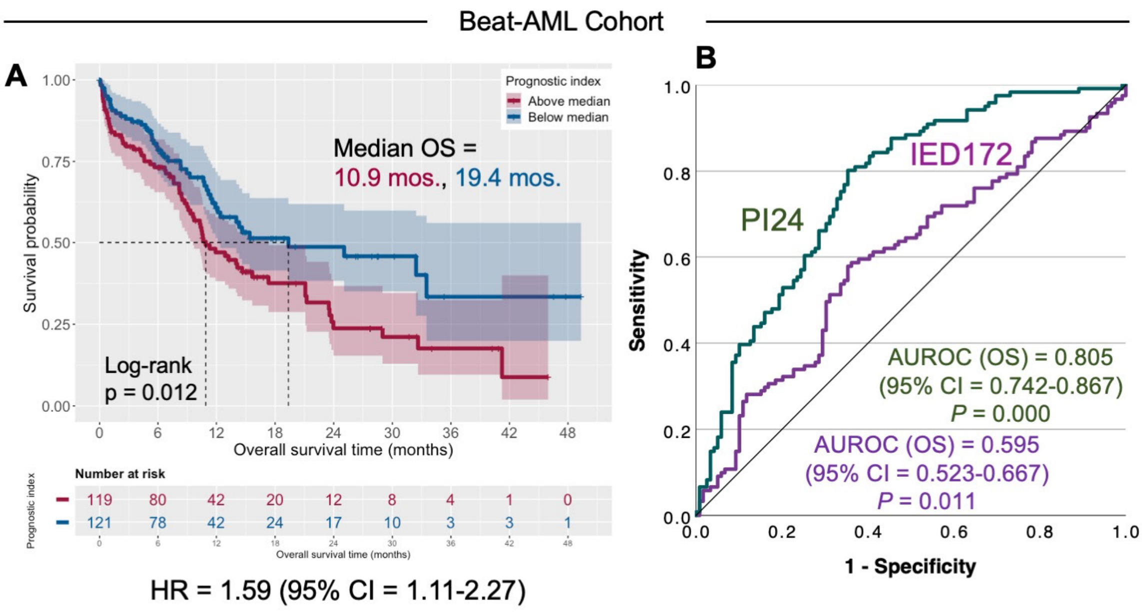

Figure S15 | An immune effector dysfunction (IED)-related prognostic index (PI24) separates survival in a validation AML cohort. Related to Figure 4.

(A) Kaplan-Meier estimates of overall survival (OS) in Beat-AML cases with PI24 above (magenta line) and below the optimal cut-point (blue line). Survival curves were compared using a log-rank test (*survminer* package in R).

(B) AUROC curve measuring the predictive ability of IED172 (magenta curve) and PI24 genes (green curve) for OS. CI = confidence interval. AUROC = 1.0 denotes perfect prediction and AUROC = 0.5 denotes no predictive ability.

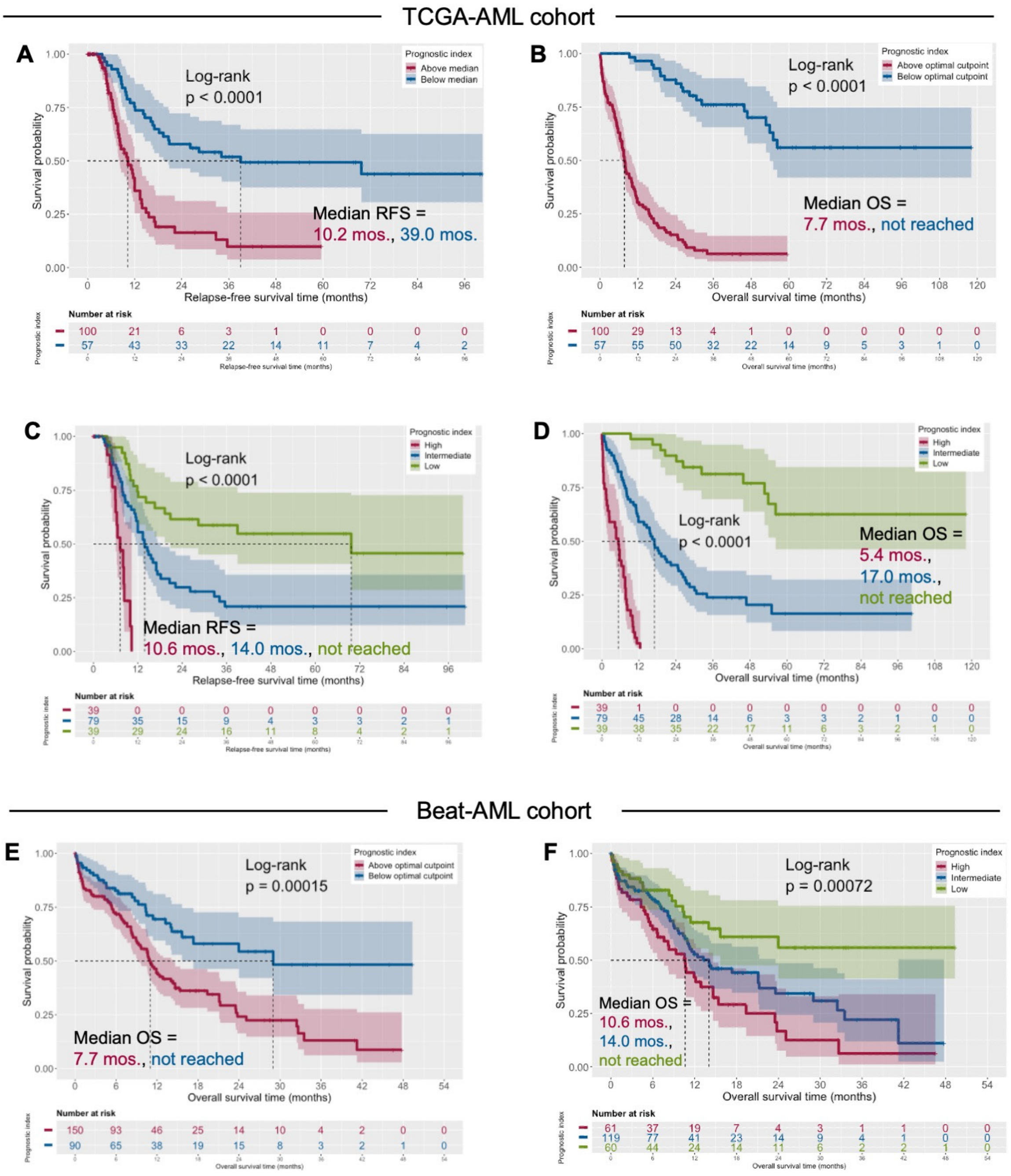

Figure S16 | Identification of an optimal prognostic index (PI24) cut-point in TCGA-AML and Beat-AML cases. Related to Figure 4.

The PI was computed as detailed in Materials and Methods.

(A) Kaplan-Meier estimates of relapse-free survival (RFS) in TCGA cases with PI24 above (magenta line) and below the optimal cut-point (blue line). Survival curves were compared using a log-rank test (*survminer* package in R).

(B) Kaplan-Meier estimates of overall survival (OS) in TCGA-AML cases with PI24 above (magenta line) and below the optimal cut-point (blue line).

(C) Kaplan-Meier estimates of RFS in TCGA cases with high (top quartile; green line), intermediate (blue line) and low PI24 (bottom quartile; magenta line).
(D) Kaplan-Meier estimates of OS in TCGA cases with high (top quartile; green line), intermediate (blue line) and low PI24 (bottom quartile; magenta line).
(E) Kaplan-Meier estimates of overall survival (OS) in Beat-AML cases with PI24 above (magenta line) and below the optimal cut-point (blue line).
(F) Kaplan-Meier estimates of OS in Beat-AML cases with high (top quartile; green line), intermediate (blue line) and low PI24 (bottom quartile; magenta line).

GSE37642 Affymetrix AML Cohort (Validation)
(N = 562 subjects treated in the German AMLCG 1999 trial)

A. GSE37642-GPL570 (n=140)

B. GSE37642-GPL96 (n=422)

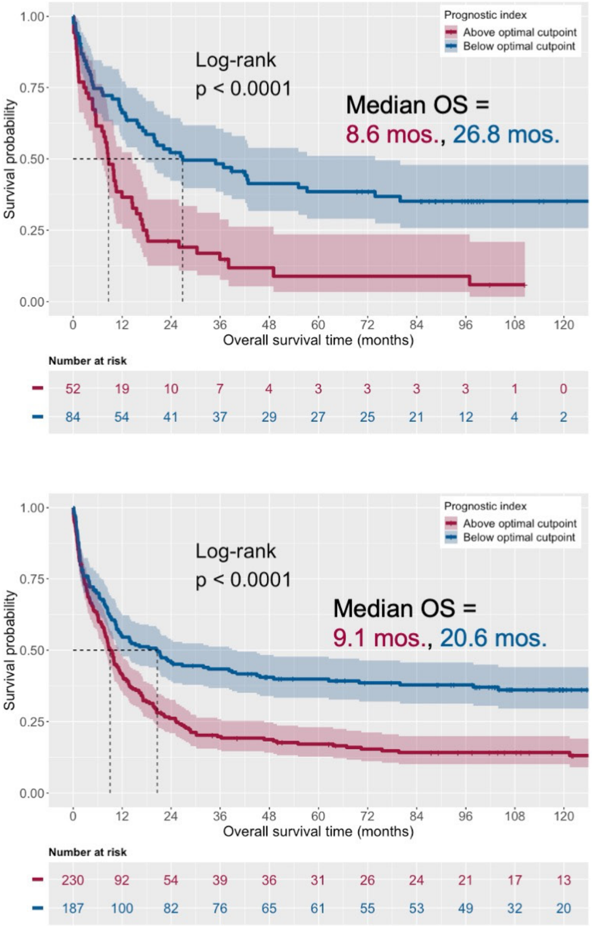

Figure S17 | Prognostic index (PI24) and survival in the GSE37642 series (German AMLCG 1999 trial). Related to Figure 4.

Gene expression data were retrieved through GEO (accession numbers: GSE37642-GPL570 and GSE37642-GPL96) (19, 20). The PI24 was computed as detailed in Materials and Methods. Maximally selected rank statistics (*maxstat* package in R) was used to identify an optimal cut-point of PI24 values.

(A) Kaplan-Meier estimate of OS in GSE37642-GPL570 cases (n = 140) with PI24 above (magenta line) and below the optimal cut-point (blue line). Survival curves were compared using a log-rank test (*survminer* package in R).

(B) Kaplan-Meier estimate of OS in GSE37642-GPL96 cases (n = 422) with PI24 above (magenta line) and below the optimal cut-point (blue line) identified in the GSE37642-GPL570 series.

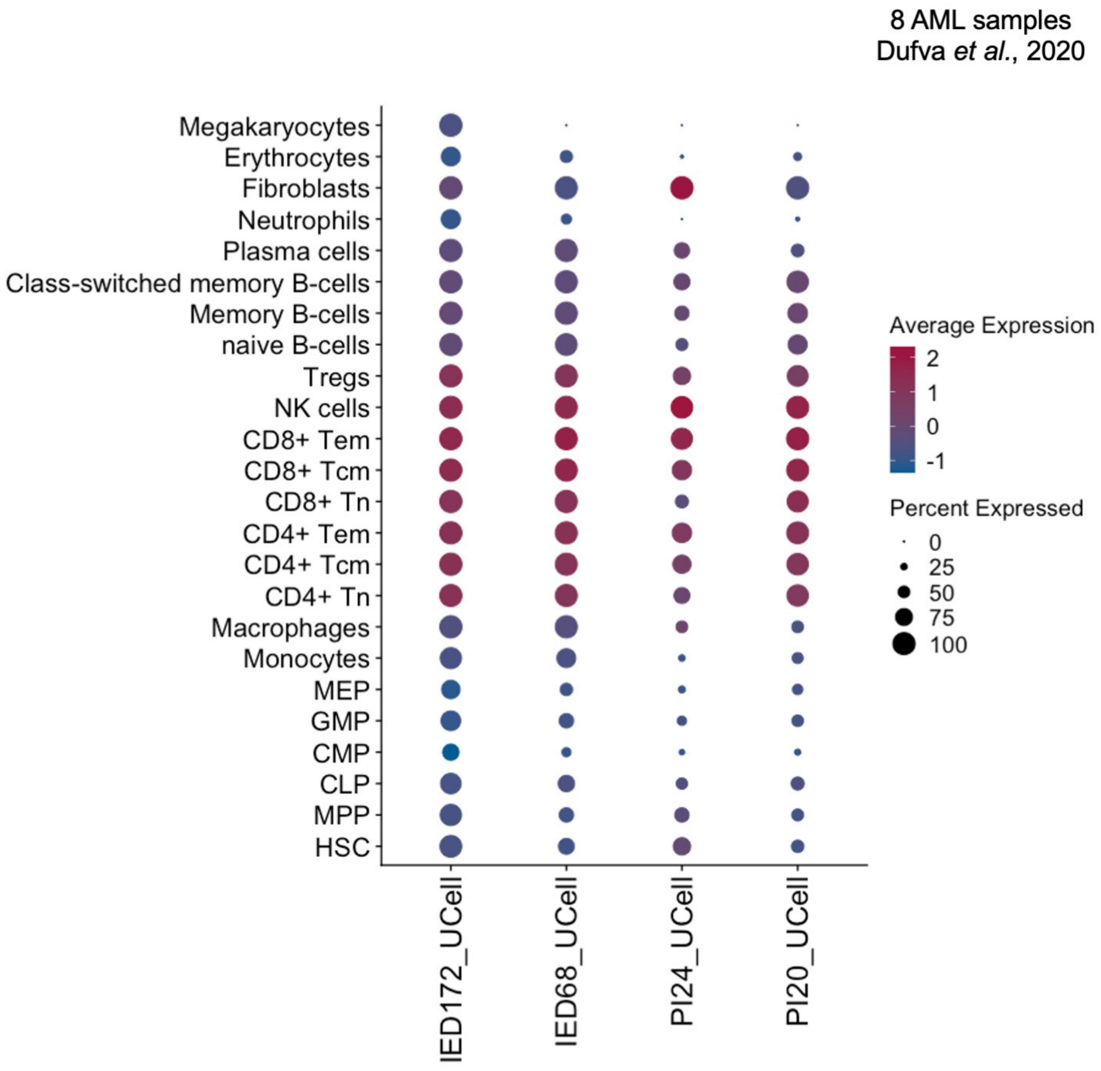

Figure S18 | Expression of IED-related gene sets in bone marrow samples from patients with AML. Related to Figure 4.

Dot plot showing the expression of IED genes (RNA-sequencing signature [IED172]; NanoString IES signature [IED68], LASSO-based RNA-sequencing IED signature [PI24] and LASSO-based NanoString IED signature [PI20]) on immune cell types annotated by Dufva *et al*. (ENCODE/Blueprint) in 8 single-cell RNA-sequencing AML samples (18). Data and R objects were retrieved from the Synapse data repository (https://www.synapse.org/#!Synapse:syn21991338; SynapseID: syn21991338) and analyzed with R v.4.2.0. MPP = multipotent progenitors; MEP = megakaryocyte erythroid progenitors; GMP = granulocyte-macrophage progenitors; CMP = common myeloid progenitors; NK = natural killer. Signature scores were calculated using the *UCell* package in R.

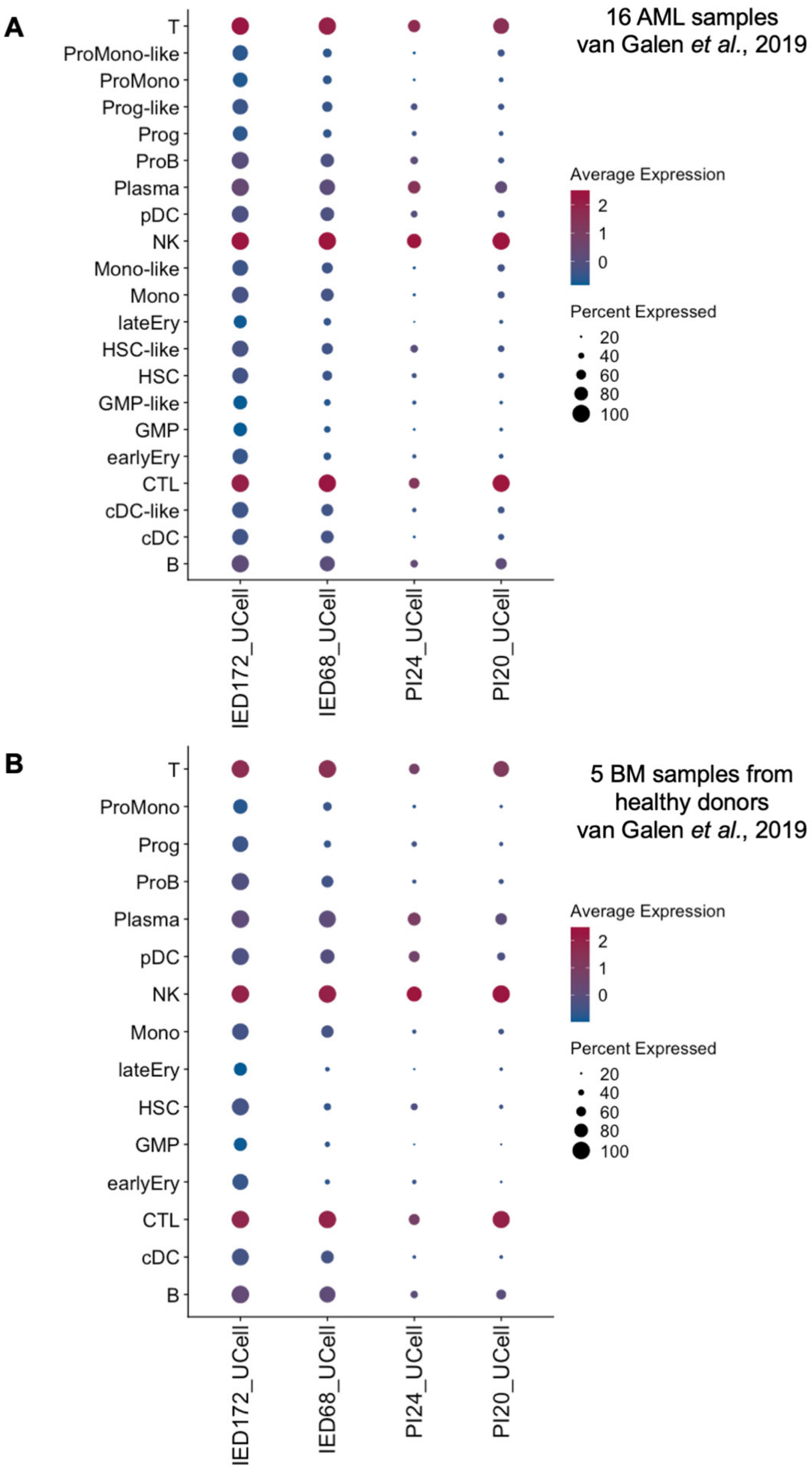

Figure S19 | Expression of IED-related gene sets in bone marrow samples from patients with AML and from healthy donors. Related to Figure 4.

(A) Dot plot showing the expression of IED genes (RNA-sequencing signature [IED172]; NanoString IED signature [IED68], LASSO-based RNA-sequencing IED signature [PI24] and LASSO-based NanoString IED signature [PI20]) on immune cell types originally annotated by van Galen *et al*. in 16 single-cell RNA-sequencing AML samples (25). Signature scores were calculated using the *UCell* package in R.

(B) Dot plot showing the expression of IED genes on immune cell types originally annotated by van Galen *et al*. in 5 single-cell RNA-sequencing BM samples from healthy donors (25).

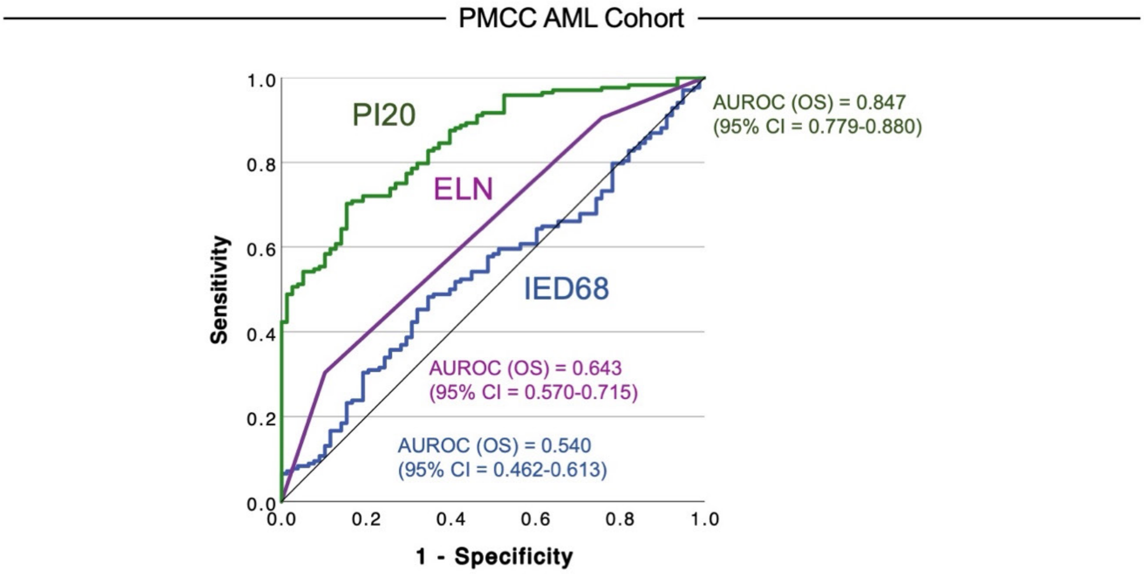

Figure S20 | Predictive ability of IED-related gene sets in patients with AML in the PMCC cohort. Related to Figure 6.

Head-to-head comparison of AUROC curves measuring the predictive ability of the IED68 score (blue line), the PI20 (blue line) and the ELN cytogenetic risk classifier (magenta line) for overall survival (OS). CI = confidence interval. AUROC = 1.0 denotes perfect prediction and AUROC = 0.5 denotes no predictive ability.

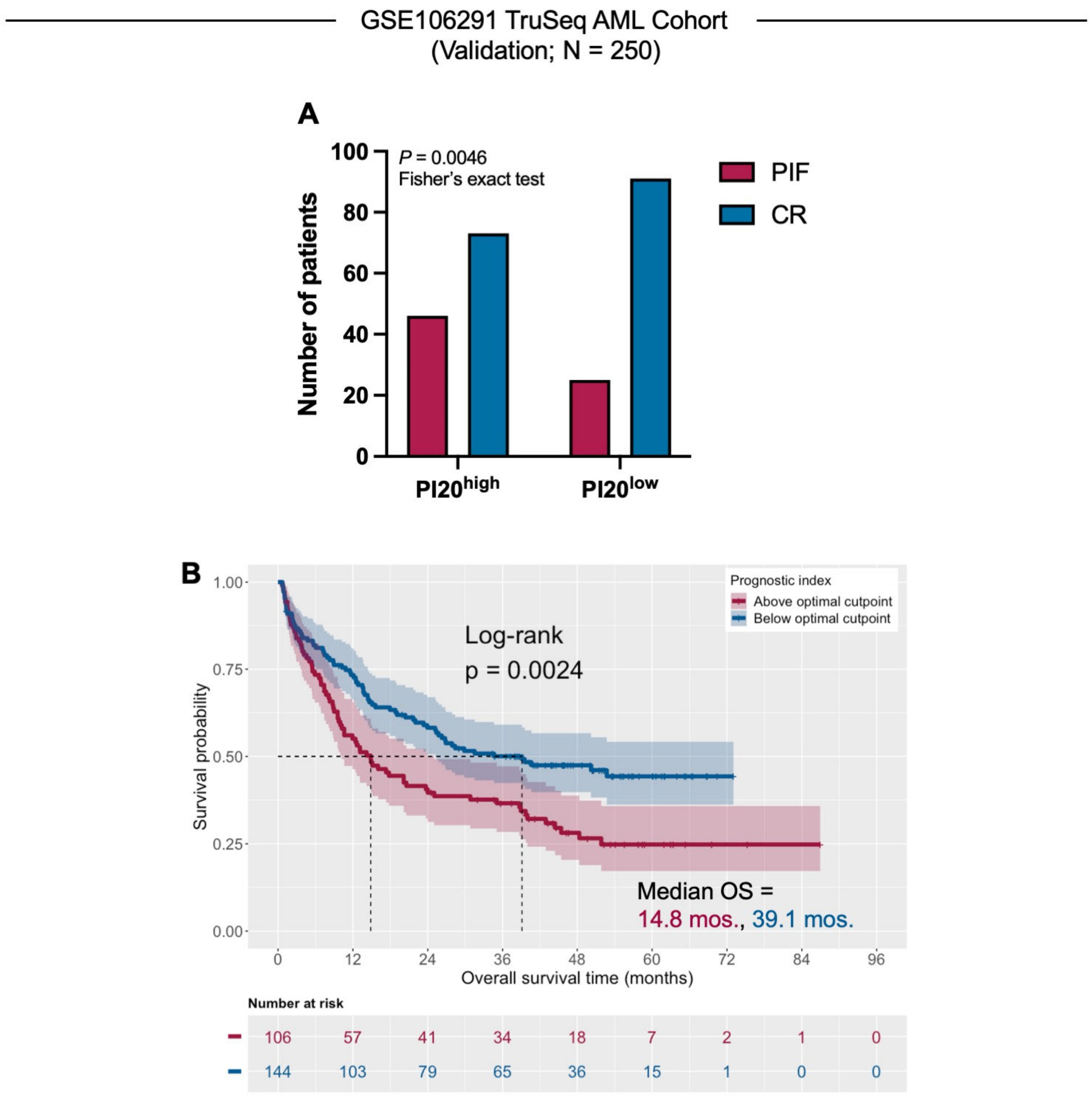

Figure S21 | Prognostic index (PI20) and primary induction failure in patients with AML (n = 250) treated in the AMLCG-2008 study (NCT01382147). Related to Figure 6. Gene expression data were retrieved through GEO (accession number: GSE106291) (19). The PI20 was computed as detailed in Materials and Methods.

(A) Number of patients with primary therapy resistance (primary induction failure; PIF) and complete response (CR) in the PI20^{high} and PI20^{low} group (median split). Data were compared using the Fisher's exact test.

(B) Kaplan-Meier estimates of overall survival (OS) in patients with PI20 above (magenta line) and below the optimal cut-point (blue line). Survival curves were compared using a log-rank test (*survminer* package in R).

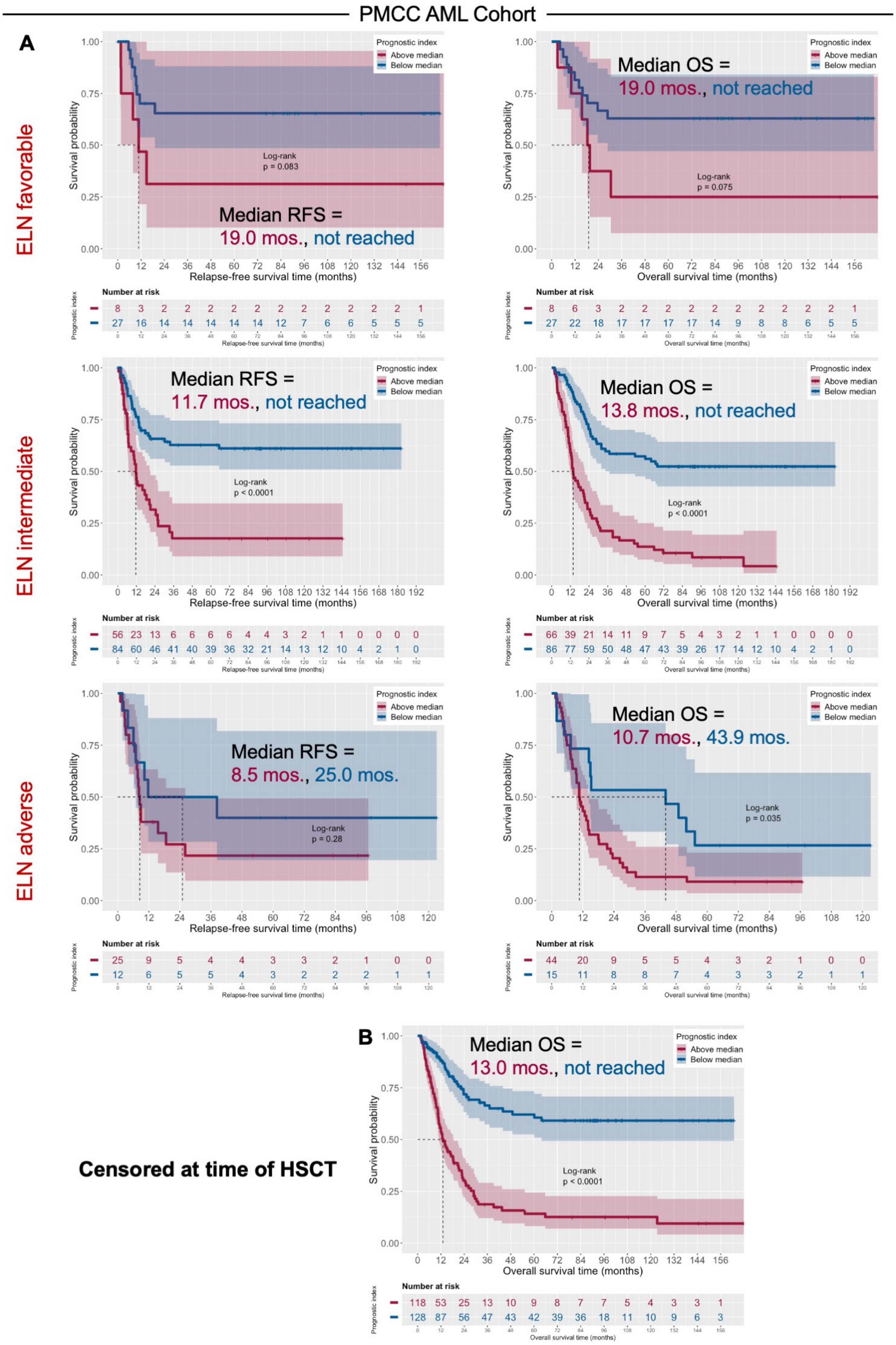
PMCC AML Cohort
A
ELN favorable
Median RFS =
19.0 mos., not reached
Log-rank
p = 0.083
Median OS =
19.0 mos., not reached
Log-rank
p = 0.075
ELN intermediate
Median RFS =
11.7 mos., not reached
Log-rank
p < 0.0001
Median OS =
13.8 mos., not reached
Log-rank
p < 0.0001
ELN adverse
Median RFS =
8.5 mos., 25.0 mos.
Log-rank
p = 0.28
Median OS =
10.7 mos., 43.9 mos.
Log-rank
p = 0.035
B
Censored at time of HSCT
Median OS =
13.0 mos., not reached
Log-rank
p < 0.0001
Prognostic index
Above median
Below median
Survival probability
Relapse-free survival time (months)
Overall survival time (months)
Number at risk

Figure S22 | NanoString-based prognostic index (PI20) in subjects of the indicated cytogenetic grouping (Princess Margaret Cancer Centre [PMCC] cohort). Related to Figure 6.

(A) Kaplan-Meier estimates of relapse-free survival (RFS; top row) and overall survival (OS; bottom row) in patients from the PMCC cohort with higher than median (magenta line) and lower than median (blue line) PI20. Survival curves were compared using a log-rank test (*survminer* package in R). ELN = 2017 European Leukemia Net.

(B) Kaplan-Meier estimates of OS after censoring at time of hematopoietic stem cell transplantation (HSCT).

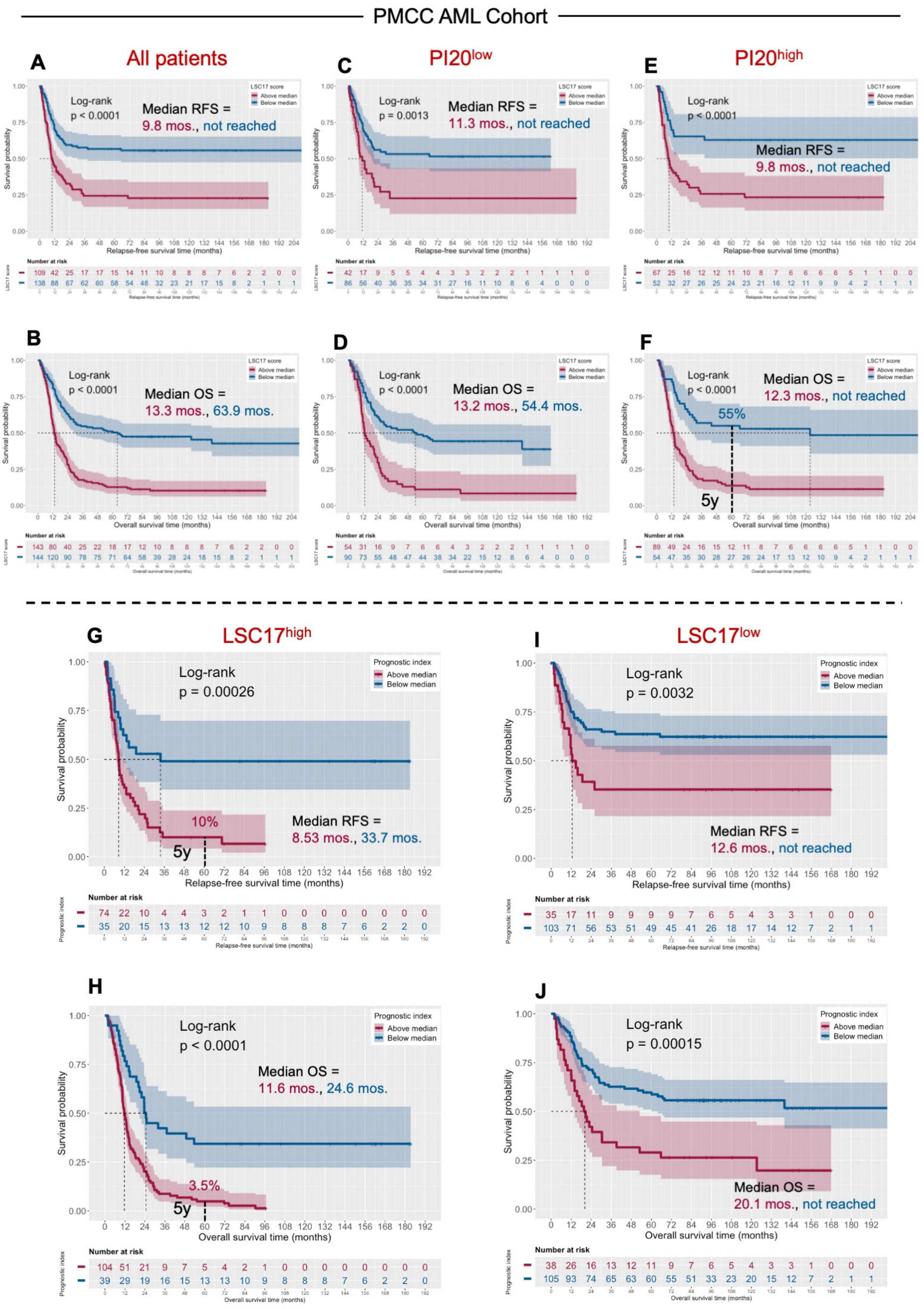

PMCC AML Cohort
A
All patients
Log-rank
p < 0.0001
Median RFS =
9.8 mos., not reached
C
PI20low
Log-rank
p = 0.0013
Median RFS =
11.3 mos., not reached
E
PI20high
Log-rank
p < 0.0001
Median RFS =
9.8 mos., not reached
B
Log-rank
p < 0.0001
Median OS =
13.3 mos., 63.9 mos.
D
Log-rank
p < 0.0001
Median OS =
13.2 mos., 54.4 mos.
F
Log-rank
p < 0.0001
Median OS =
12.3 mos., not reached
55%
5y
G
LSC17high
Log-rank
p = 0.00026
Prognostic index
Above median
Below median
10%
5y
Median RFS =
8.53 mos., 33.7 mos.
Survival probability
Relapse-free survival time (months)
Number at risk
I
LSC17low
Log-rank
p = 0.0032
Median RFS =
12.6 mos., not reached
H
Log-rank
p < 0.0001
Median OS =
11.6 mos., 24.6 mos.
3.5%
5y
Overall survival time (months)
J
Log-rank
p = 0.00015
Median OS =
20.1 mos., not reached

Figure S23 | NanoString-based prognostic index (PI20) and survival in LSC17^{high} and LSC17^{low} patients (Princess Margaret Cancer Centre [PMCC] cohort). Related to Figure 6.

Kaplan-Meier estimates of relapse-free survival (RFS) and overall survival (OS) in PI20^{low} and PI20^{high} patients from the PMCC cohort with higher than median (magenta line) and lower than median (blue line) LSC17 scores, which were computed as detailed in Materials and Methods and in the original publication (12). Panels A and B = all patients; panels C and D = PI^{low} patients; panels E and F = PI^{high} patients. RNA-sequencing data were retrieved through GEO (accession number: GSE76004). Survival curves were compared using a log-rank test (*survminer* package in R).

Kaplan-Meier estimates of RFS and OS in patients with higher than median (magenta line) and lower than median (blue line) PI stratified based on a median split of the LSC17 stemness score. Panels G and H = LSC17^{high} patients; panels I and J = LSC17^{low} patients.

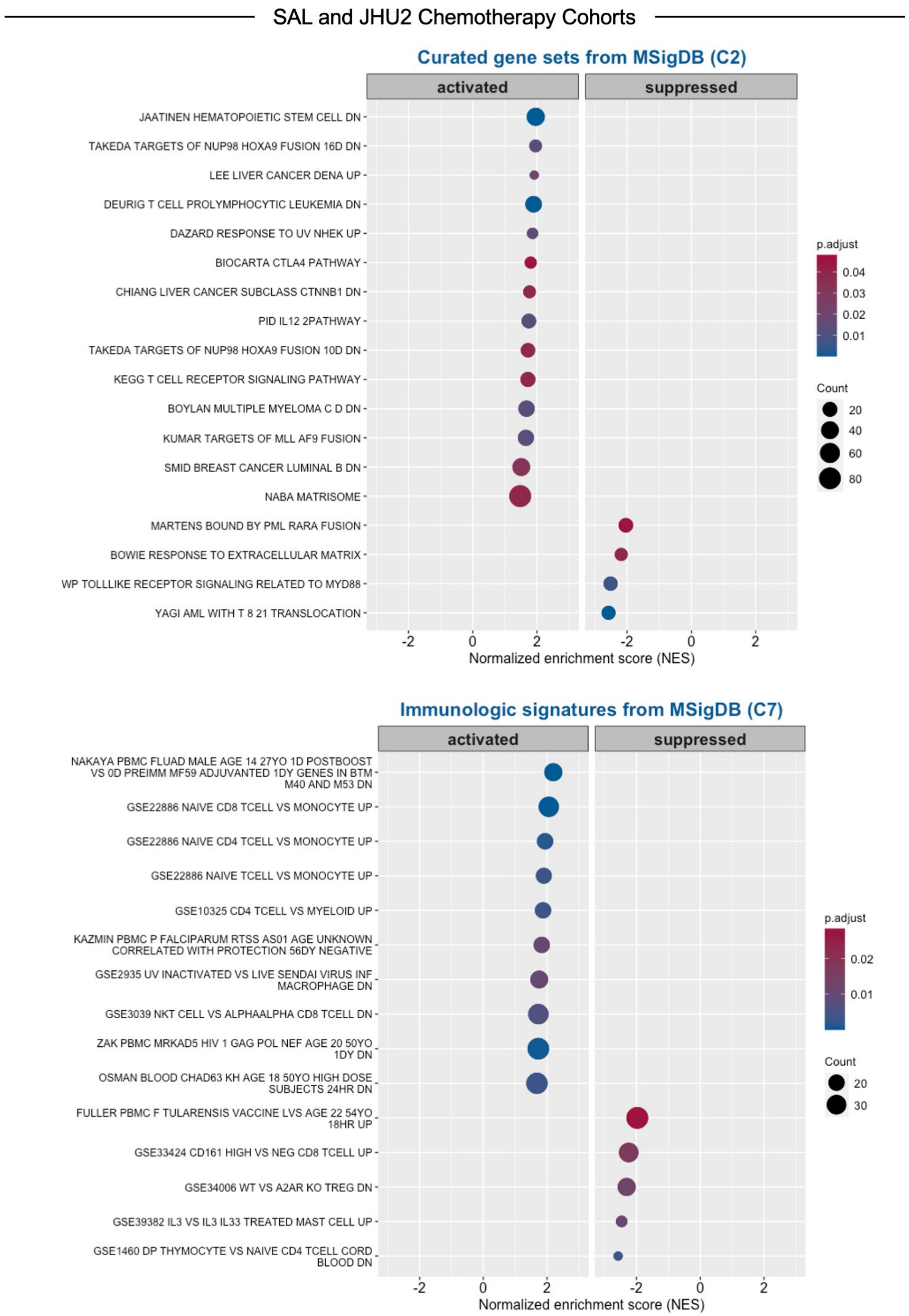

Figure S24 | Gene set enrichment analysis (GSEA) of differentially expressed genes between baseline and post-chemotherapy bone marrow samples from patients in the SAL and JHU2 chemotherapy cohorts. Related to Figure 8.

GSEA was performed using the *clusterProfiler* package in R using differentially expressed genes between baseline and post-chemotherapy bone marrow samples ($\log_2$ fold-change >1.0; adjusted P value <0.05) as an input. C2 and C7 gene sets were downloaded from the MSigDB (https://www.gsea-msigdb.org/gsea/msigdb/). SAL = Studien Allianz Leukämie; JHU = Johns Hopkins University.

Single-Cell RNA-Sequencing AML Immunotherapy Cohort (Abbas *et al*., Nat. Comms. 2021)

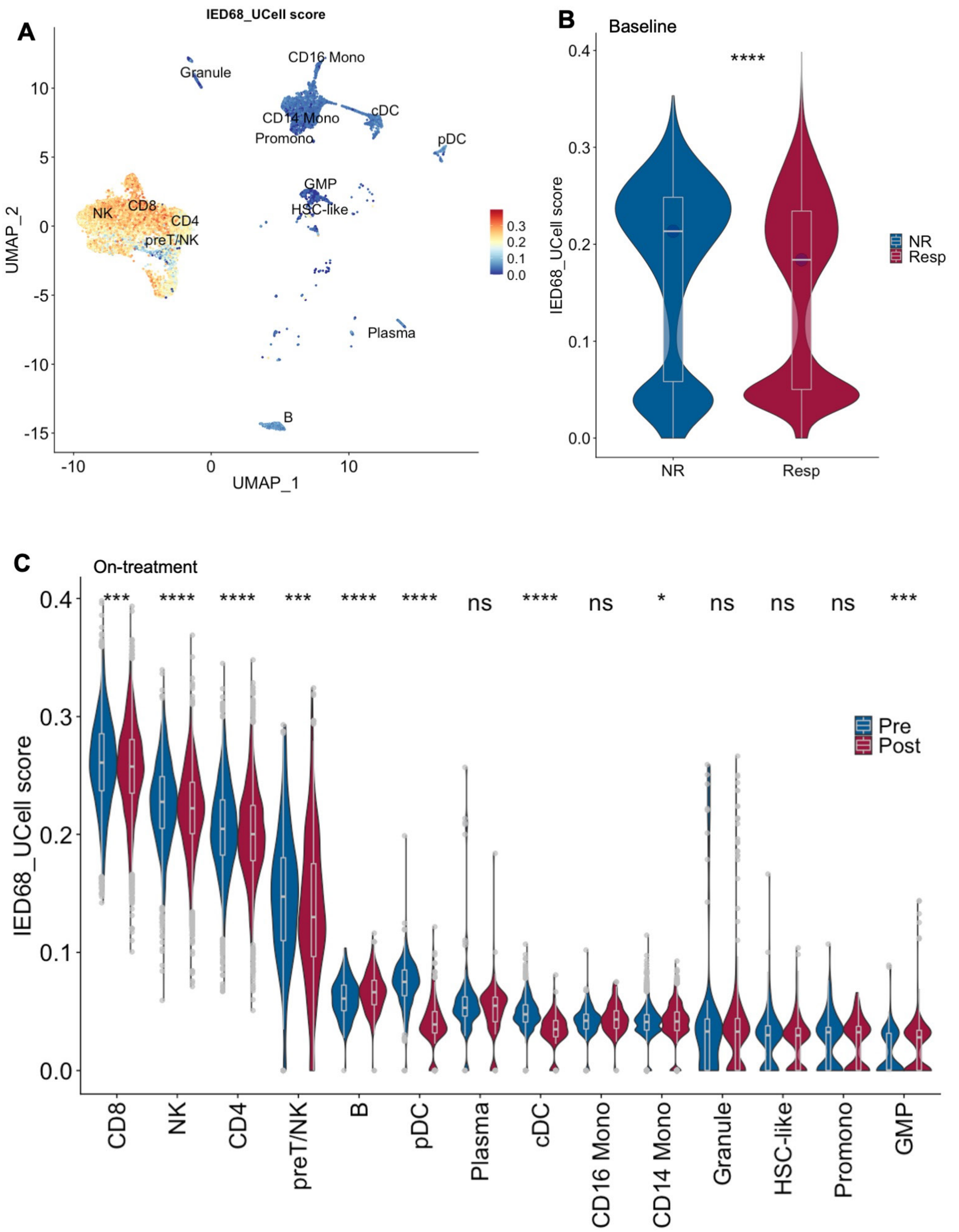

Figure S25 | Expression of IED68 genes in a single-cell RNA-sequencing cohort of 8 patients with chemotherapy-refractory and/or relapsed AML treated with azacitidine and nivolumab immunotherapy. Related to Figure 9.

(A) Uniform manifold approximation and projection (UMAP) embedding of IED68 genes in primary bone marrow (BM) samples (27). Cell type annotation as in the original publication. The IED68 score was computed using the *UCell* package in R.

(B) Violin plot of IED68 single-cell scores in primary BM samples. NR = non-responder; Resp = responder. Data were compared using the Wilcoxon matched pairs signed rank test. **** *P*<0.001.

(C) Violin plot of IED68 single-cell scores in primary BM samples collected at baseline and on-treatment. Data were compared using the Wilcoxon matched pairs signed rank test. * *P*<0.05; *** *P*<0.001; **** *P*<0.0001.

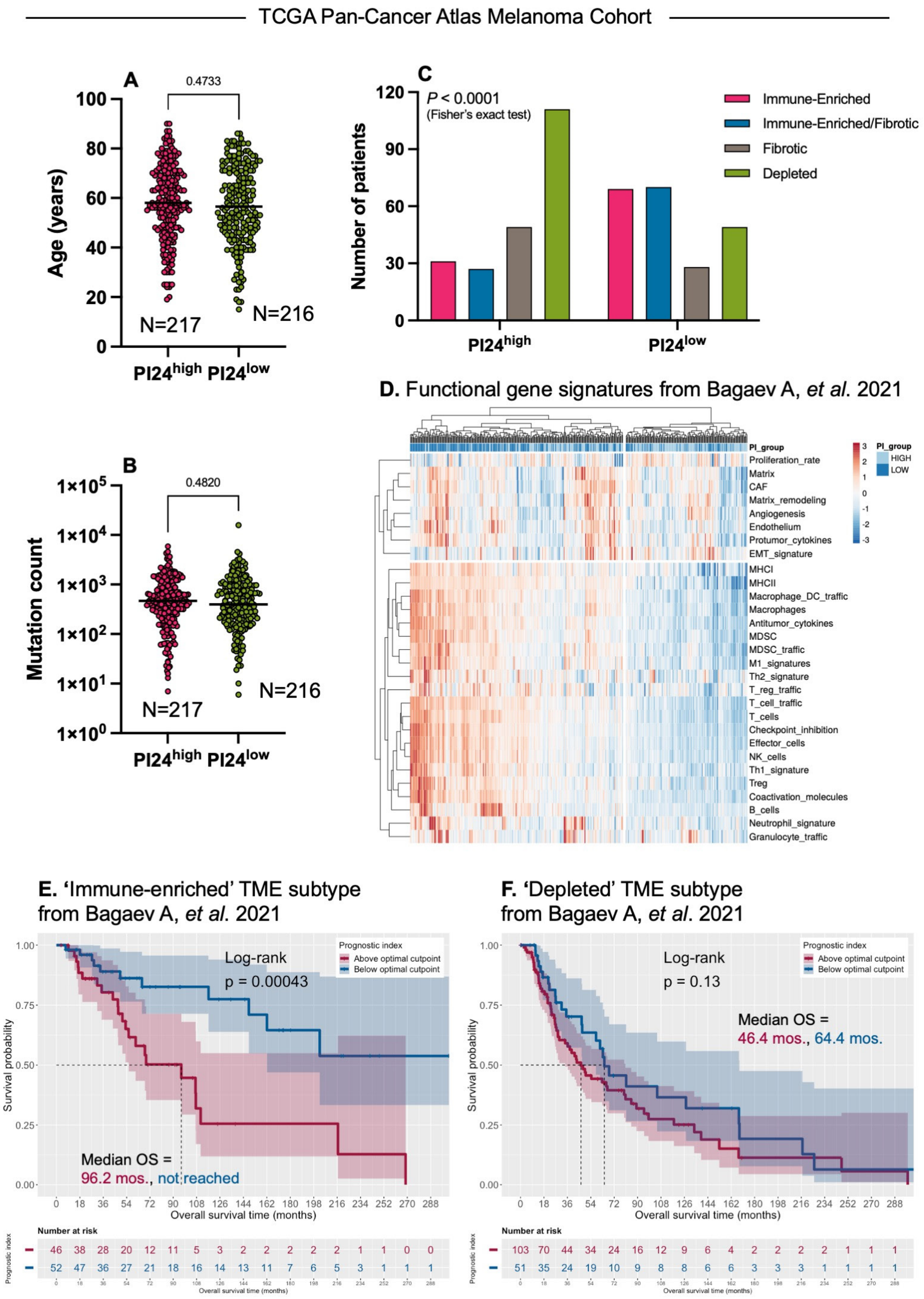

Figure S26 | IED scores in patients with cutaneous melanoma (TCGA series). Related to Figure 10. RNA-sequencing and outcomes data for 441 patients with primary and/or

metastatic cutaneous melanoma (TCGA Pan-Cancer Atlas profiling project) were retrieved through the cBioPortal for Cancer Genomics (https://www.cbioportal.org/) (21).

(A) Age (years) in patients with above and below median prognostic index (PI24). Data were compared using the Mann-Whitney *U* test for unpaired determinations.

(B) Mutation count in patients with above and below median PI24. Data were compared using the Mann-Whitney *U* test for unpaired determinations.

(C) Tumor microenvironment (TME) subtypes, as recently defined (22), in patients with above and below median PI24. Data were compared using the Fisher's exact test. Sample annotation was retrieved through the BostonGene Science Portal (https://science.bostongene.com/tumor-portrait/). (D) Functional gene expression signatures from Bagaev *et al*. (22) in $PI24^{high}$ and $PI24^{low}$ cases. ClustVis, an online tool for clustering of multivariate data (Euclidean distance, complete linkage), was used for data visualization (37). The heatmap annotation track shows median split of PI24 scores.

(E) Overall survival of patients with an immune-enriched TME stratified by PI24 [above (magenta line) and below the optimal cut-point (blue line); *maxstat* package in R]. Survival curves were compared using a log-rank test (*survminer* package in R).

(F) Overall survival of patients with a depleted TME stratified by PI24 [above (magenta line) and below the optimal cut-point (blue line)].

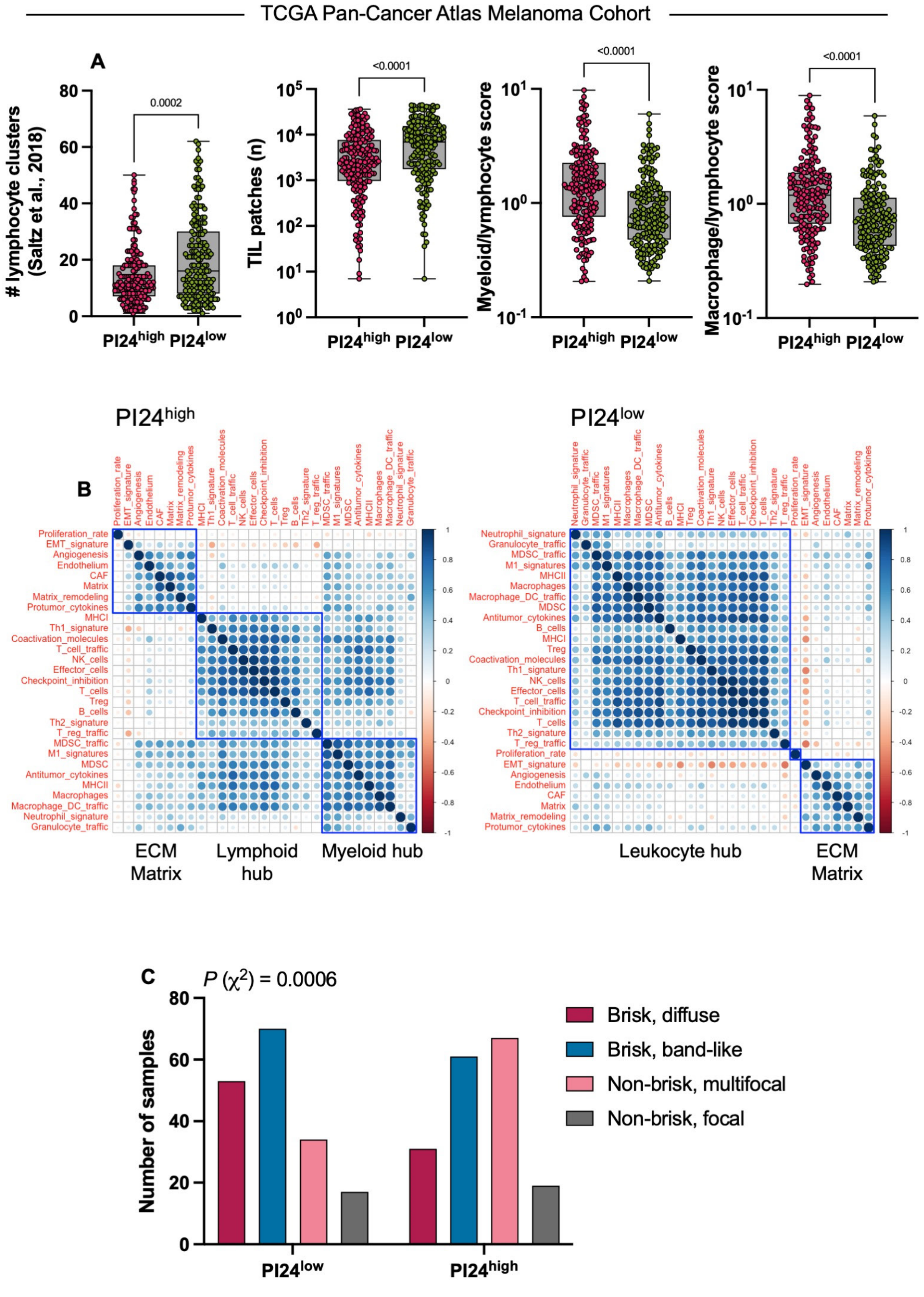

Figure S27 | PI24 scores in patients with cutaneous melanoma (TCGA series). Related to Figure 10.

(A) Box plots showing the number of lymphocyte clusters and tumor infiltrating lymphocyte (TIL) patches (available from Saltz *et al*. (38)), as well as myeloid/lymphocyte RNA scores (available from Bagaev *et al*. (22)), in patients with melanoma in the PI24^{high} and PI24^{low} subgroup. Data were compared using the Mann-Whitney *U* test for unpaired determinations.
(B) Correlograms showing co-expression of functional gene signatures (available from Bagaev *et al*.) in PI24^{high} and PI24^{low} cases. The correlation matrix was re-ordered using the *hclust* function (corrplot package in R). Rectangles ("signature hubs") were drawn based on the results of hierarchical clustering (Euclidean distance, complete linkage).
(C) TIL patterns from deep-learning-derived "computational stain" of melanoma tissues (available from Saltz *et al*. (38)) from patients in the PI24^{high} and PI24^{low} subgroup. Fisher's exact test.

Single-Cell RNA-Sequencing Melanoma Cohort (Tirosh *et al.*, Science 2016)

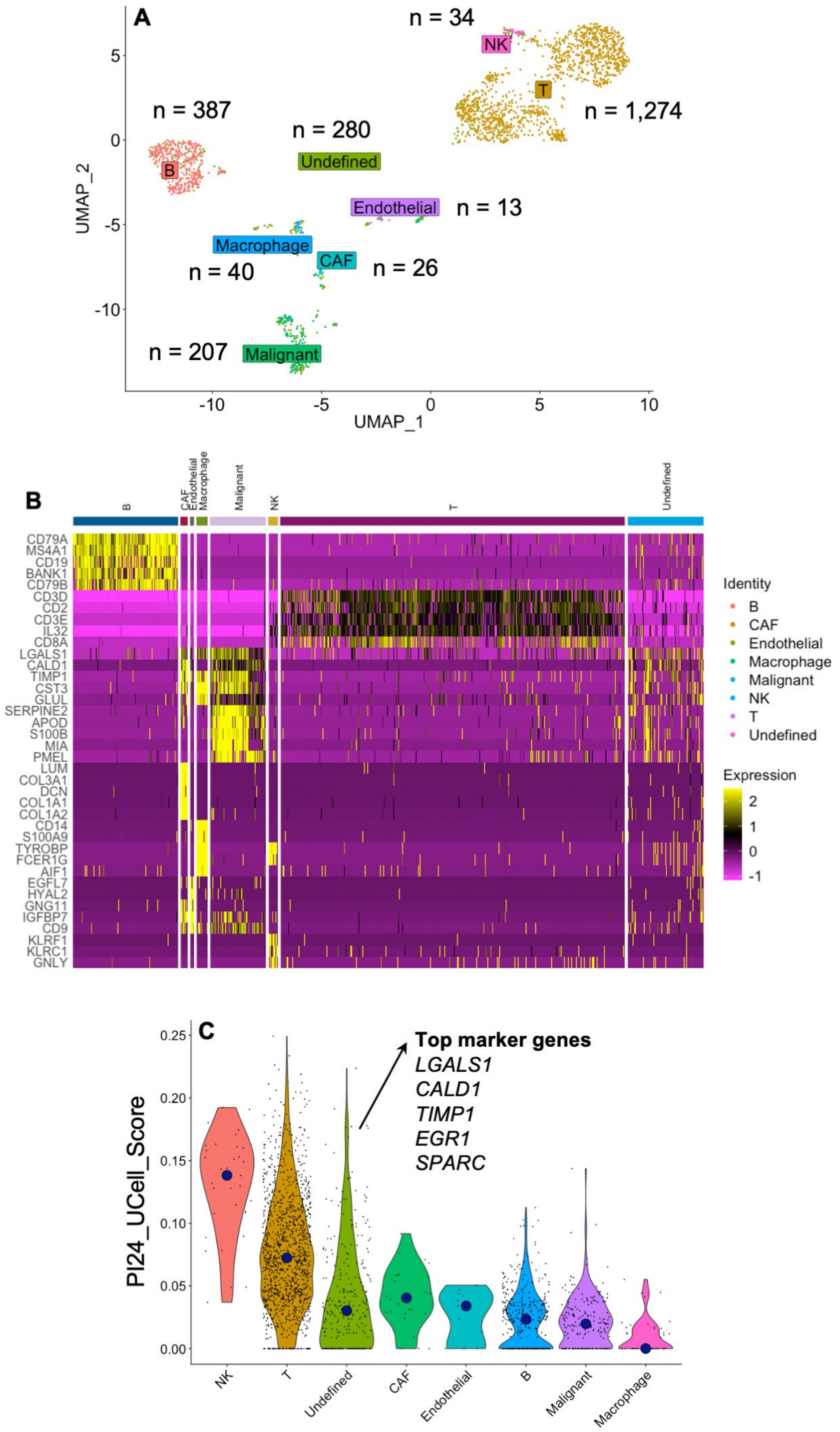

Figure S28 | Expression of PI24 genes in a single-cell RNA-sequencing cohort encompassing 19 human melanoma tumors (Tirosh et al.). Related to Figure 10.

(A) Uniform manifold approximation and projection (UMAP) embedding of PI24 genes in primary melanoma samples from Tirosh *et al*. (28). Cell type annotation as in the original publication.
(B) Heatmap showing expression of the top 5 marker genes which were identified using the *FindAllMarkers* function in Seurat. The MAST package was used to run differential expression testing.
(C) Violin plot of the expression of PI24 genes in primary melanoma tissues. The PI24 score was computed using the *UCell* package in R.

Single-Cell RNA-Sequencing Melanoma Immunotherapy Cohort (Sade-Feldman *et al.*, Cell 2018)

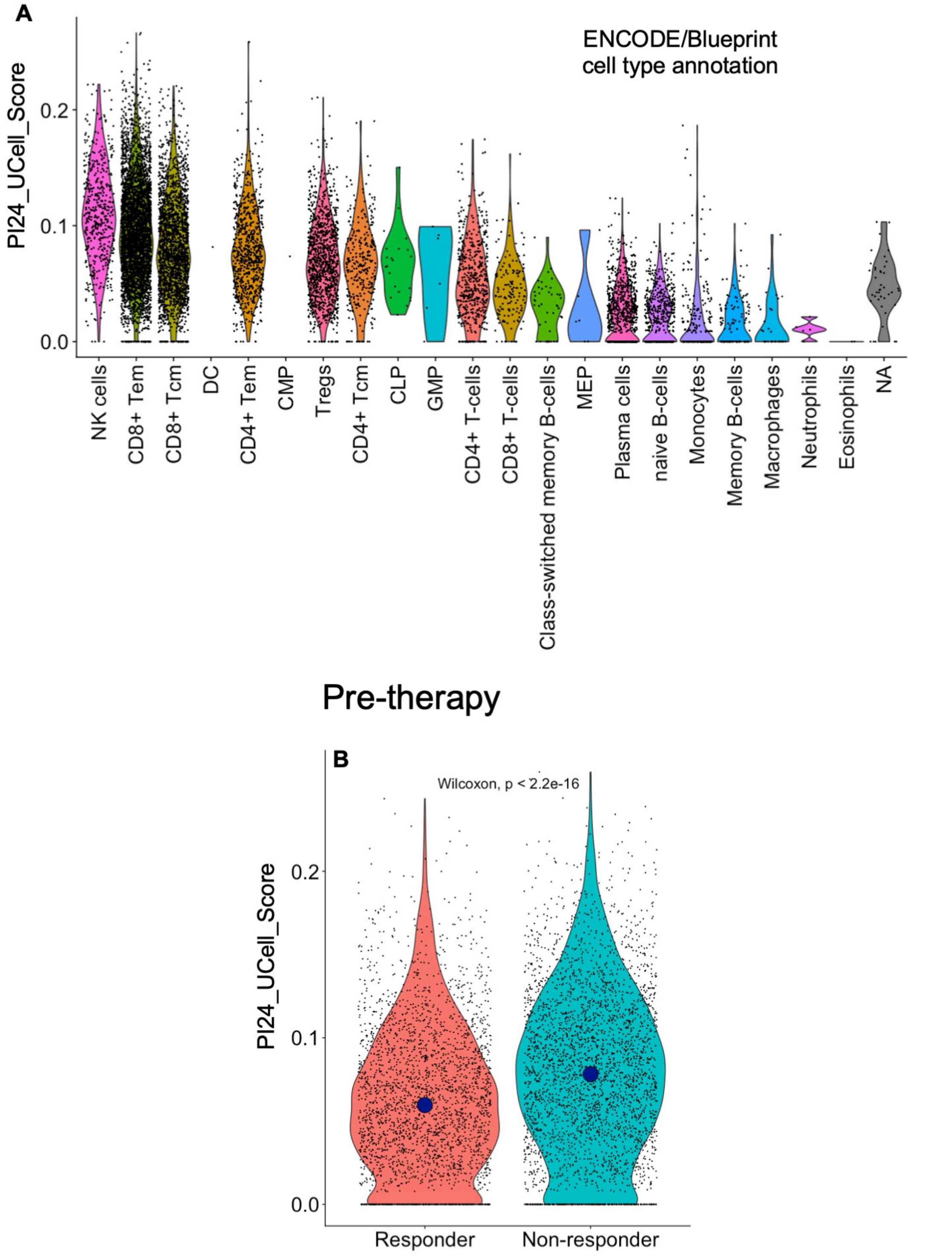

Figure S29 | Expression of PI24 genes in a single-cell RNA-seq cohort encompassing 48 tumor samples of patients with melanoma treated with immune checkpoint blockade (Sade-Feldman et al.). Related to Figure 10.

(A) Violin plot of the expression of PI24 genes in primary melanoma tissues from Sade-Feldman *et al*. (29). The PI24 score was computed using the *UCell* package in R. Cells were

automatically annotated (ENCODE/Blueprint reference map) using the *SingleR* and *celldex* packages in R (30, 31).

(B) Violin plot of the expression of PI24 genes in pre-therapy melanoma tissues from responders and non-responders to immune checkpoint blockade (ICB).

REFERENCES

1. Vadakekolathu J, Minden MD, Hood T, Church SE, Reeder S, Altmann H, et al. Immune landscapes predict chemotherapy resistance and immunotherapy response in acute myeloid leukemia. *Sci Transl Med.* 2020;12(546):eaaz0463.

2. Krupka C, Kufer P, Kischel R, Zugmaier G, Bogeholz J, Kohnke T, et al. CD33 target validation and sustained depletion of AML blasts in long-term cultures by the bispecific T-cell-engaging antibody AMG 330. *Blood.* 2014;123(3):356-65.

3. Ley TJ, Miller C, Ding L, Raphael BJ, Mungall AJ, Robertson A, et al. Genomic and epigenomic landscapes of adult de novo acute myeloid leukemia. *N Engl J Med.* 2013;368(22):2059-74.

4. Tyner JW, Tognon CE, Bottomly D, Wilmot B, Kurtz SE, Savage SL, et al. Functional genomic landscape of acute myeloid leukaemia. *Nature.* 2018;562(7728):526-31.

5. Burd A, Levine RL, Ruppert AS, Mims AS, Borate U, Stein EM, et al. Precision medicine treatment in acute myeloid leukemia using prospective genomic profiling: feasibility and preliminary efficacy of the Beat AML Master Trial. *Nat Med.* 2020;26(12):1852-8.

6. Bolouri H, Farrar JE, Triche T, Jr., Ries RE, Lim EL, Alonzo TA, et al. The molecular landscape of pediatric acute myeloid leukemia reveals recurrent structural alterations and age-specific mutational interactions. *Nat Med.* 2018;24(1):103-12.

7. Farrar JE, Schuback HL, Ries RE, Wai D, Hampton OA, Trevino LR, et al. Genomic profiling of pediatric acute myeloid leukemia reveals a changing mutational landscape from disease diagnosis to relapse. *Cancer Res.* 2016;76(8):2197-205.

8. Gide TN, Quek C, Menzies AM, Tasker AT, Shang P, Holst J, et al. Distinct immune cell populations define response to anti-PD-1 monotherapy and anti-PD-1/anti-CTLA-4 combined therapy. *Cancer Cell.* 2019;35(2):238-55 e6.

9. Jiang P, Gu S, Pan D, Fu J, Sahu A, Hu X, et al. Signatures of T cell dysfunction and exclusion predict cancer immunotherapy response. *Nat Med.* 2018;24(10):1550-8.

10. Danaher P, Warren S, Dennis L, D'Amico L, White A, Disis ML, et al. Gene expression markers of Tumor Infiltrating Leukocytes. *J Immunother Cancer.* 2017;5:18.

11. Vadakekolathu J, Lai C, Reeder S, Church SE, Hood T, Lourdusamy A, et al. TP53 abnormalities correlate with immune infiltration and associate with response to flotetuzumab immunotherapy in AML. *Blood Adv.* 2020;4(20):5011-24.

12. Ng SW, Mitchell A, Kennedy JA, Chen WC, McLeod J, Ibrahimova N, et al. A 17-gene stemness score for rapid determination of risk in acute leukaemia. *Nature.* 2016;540(7633):433-7.

13. Godec J, Tan Y, Liberzon A, Tamayo P, Bhattacharya S, Butte AJ, et al. Compendium of immune signatures identifies conserved and species-specific biology in response to inflammation. *Immunity.* 2016;44(1):194-206.

14. Subramanian A, Tamayo P, Mootha VK, Mukherjee S, Ebert BL, Gillette MA, et al. Gene set enrichment analysis: a knowledge-based approach for interpreting genome-wide expression profiles. *Proc Natl Acad Sci U S A.* 2005;102(43):15545-50.

15. Finotello F, Mayer C, Plattner C, Laschober G, Rieder D, Hackl H, et al. Molecular and pharmacological modulators of the tumor immune contexture revealed by deconvolution of RNA-seq data. *Genome Med.* 2019;11(1):34.

16. Sturm G, Finotello F, Petitprez F, Zhang JD, Baumbach J, Fridman WH, et al. Comprehensive evaluation of transcriptome-based cell-type quantification methods for immuno-oncology. *Bioinformatics.* 2019;35(14):i436-i45.

17. Tibshirani R. The lasso method for variable selection in the Cox model. *Stat Med.* 1997;16(4):385-95.

18. Dufva O, Polonen P, Bruck O, Keranen MAI, Klievink J, Mehtonen J, et al. Immunogenomic landscape of hematological malignancies. *Cancer Cell.* 2020;38(3):380-99 e13.

19. Herold T, Jurinovic V, Batcha AMN, Bamopoulos SA, Rothenberg-Thurley M, Ksienzyk B, et al. A 29-gene and cytogenetic score for the prediction of resistance to induction treatment in acute myeloid leukemia. *Haematologica.* 2018;103(3):456-65.

20. Li Z, Herold T, He C, Valk PJ, Chen P, Jurinovic V, et al. Identification of a 24-gene prognostic signature that improves the European LeukemiaNet risk classification of acute myeloid leukemia: an international collaborative study. *J Clin Oncol.* 2013;31(9):1172-81.

21. Gao J, Aksoy BA, Dogrusoz U, Dresdner G, Gross B, Sumer SO, et al. Integrative analysis of complex cancer genomics and clinical profiles using the cBioPortal. *Sci Signal.* 2013;6(269):pl1.

22. Bagaev A, Kotlov N, Nomie K, Svekolkin V, Gafurov A, Isaeva O, et al. Conserved pan-cancer microenvironment subtypes predict response to immunotherapy. *Cancer Cell.* 2021;39(6):845-65 e7.

23. Hao Y, Hao S, Andersen-Nissen E, Mauck WM, 3rd, Zheng S, Butler A, et al. Integrated analysis of multimodal single-cell data. *Cell.* 2021;184(13):3573-87 e29.

24. Yang C, Siebert JR, Burns R, Gerbec ZJ, Bonacci B, Rymaszewski A, et al. Heterogeneity of human bone marrow and blood natural killer cells defined by single-cell transcriptome. *Nat Commun.* 2019;10(1):3931.

25. van Galen P, Hovestadt V, Wadsworth Ii MH, Hughes TK, Griffin GK, Battaglia S, et al. Single-cell RNA-Seq reveals AML hierarchies relevant to disease progression and immunity. *Cell.* 2019;176(6):1265-81 e24.

26. Hafemeister C, and Satija R. Normalization and variance stabilization of single-cell RNA-seq data using regularized negative binomial regression. *Genome Biol.* 2019;20(1):296.

27. Abbas HA, Hao D, Tomczak K, Barrodia P, Im JS, Reville PK, et al. Single cell T cell landscape and T cell receptor repertoire profiling of AML in context of PD-1 blockade therapy. *Nat Commun.* 2021;12(1):6071.

28. Tirosh I, Izar B, Prakadan SM, Wadsworth MH, 2nd, Treacy D, Trombetta JJ, et al. Dissecting the multicellular ecosystem of metastatic melanoma by single-cell RNA-seq. *Science.* 2016;352(6282):189-96.

29. Sade-Feldman M, Yizhak K, Bjorgaard SL, Ray JP, de Boer CG, Jenkins RW, et al. Defining T Cell States Associated with Response to Checkpoint Immunotherapy in Melanoma. *Cell.* 2018;175(4):998-1013 e20.

30. Aran D, Looney AP, Liu L, Wu E, Fong V, Hsu A, et al. Reference-based analysis of lung single-cell sequencing reveals a transitional profibrotic macrophage. *Nat Immunol.* 2019;20(2):163-72.

31. Martens JH, and Stunnenberg HG. BLUEPRINT: mapping human blood cell epigenomes. *Haematologica.* 2013;98(10):1487-9.

32. Miller BC, Sen DR, Al Abosy R, Bi K, Virkud YV, LaFleur MW, et al. Subsets of exhausted CD8+ T cells differentially mediate tumor control and respond to checkpoint blockade. *Nat Immunol.* 2019;20(3):326-36.

33. Gueguen P, Metoikidou C, Dupic T, Lawand M, Goudot C, Baulande S, et al. Contribution of resident and circulating precursors to tumor-infiltrating CD8+ T cell populations in lung cancer. *Sci Immunol.* 2021;6(55).

34. Guo X, Zhang Y, Zheng L, Zheng C, Song J, Zhang Q, et al. Global characterization of T cells in non-small-cell lung cancer by single-cell sequencing. *Nat Med.* 2018;24(7):978-85.

35. Wagner S, Vadakekolathu J, Tasian SK, Altmann H, Bornhauser M, Pockley AG, et al. A parsimonious 3-gene signature predicts clinical outcomes in an acute myeloid leukemia multicohort study. *Blood Adv.* 2019;3(8):1330-46.

36. Pereira BI, De Maeyer RPH, Covre LP, Nehar-Belaid D, Lanna A, Ward S, et al. Sestrins induce natural killer function in senescent-like CD8+ T cells. *Nat Immunol.* 2020;21(6):684-94.

37. Metsalu T, and Vilo J. ClustVis: a web tool for visualizing clustering of multivariate data using Principal Component Analysis and heatmap. *Nucleic Acids Res.* 2015;43(W1):W566-70.

38. Saltz J, Gupta R, Hou L, Kurc T, Singh P, Nguyen V, et al. Spatial organization and molecular correlation of tumor-infiltrating lymphocytes using deep learning on pathology images. *Cell Rep.* 2018;23(1):181-93 e7.
